# A PDGFRB-CD44-MIF crosstalk promotes cancer-associated cellular phenotypes in ATRT-TYR/MYC cell lines

**DOI:** 10.3389/fonc.2026.1921228

**Published:** 2026-09-09

**Authors:** Yacine A. Choutri, Liming Xu, Fupan Yao, Annie Huang

**Affiliations:** 1 Department of Medical Biophysics, University of Toronto, Toronto, ON, Canada; 2 Research Program in Cell Biology, Hospital for Sick Children, Toronto, ON, Canada; 3 Arthur and Sonia Labatt Brain Tumour Research Centre, Hospital for Sick Children, Toronto, ON, Canada; 4 SickKids Research Institute, Toronto, ON, Canada

**Keywords:** ATRT, CD44, EMT, MIF, PDGFRB

## Abstract

**Objective:**

Atypical teratoid/rhabdoid tumor (ATRT) is the most common brain tumor in children less than one-year-old. Previous studies have identified three epigenetic subgroups of ATRT: ATRT-SHH, ATRT-TYR, and ATRT-MYC. Interestingly, it was found that ATRT-TYR/MYC (mesenchymal) subgroups are sensitive to receptor-tyrosine kinase inhibitors (RTKIs), particularly those that inhibit the platelet-derived growth factor receptor B (PDGFRB), highlighting the importance of PDGF signaling in ATRTs. In addition, the ATRT-TYR/MYC subgroups show upregulation of the macrophage migration inhibitory factor (MIF), with dysregulation of the MIF signaling pathway, which was found to have immunosuppressive effects in other cancers. While PDGF and MIF pathways have been found to promote tumorigenesis of other cancers including glioma, their roles in ATRTs remain unknown. We hypothesized that PDGF and MIF signaling pathways contribute to maintaining malignant phenotypes in ATRT-TYR/MYC subgroups.

**Methods:**

To test this hypothesis, our project aimed to characterize PDGF signaling and investigate PDGF-MIF crosstalk in ATRT-TYR/MYC subgroups, using CRISPR/Cas9 stable knockouts (KOs) of various PDGF receptor and ligands.

**Results:**

We have shown that the PDGF pathway primarily promotes maintenance of malignant phenotypes in ATRT-TYR/MYC via modulation of the cell cycle. Furthermore, our studies reveal a plausible PDGFRB, MIF, and CD44 oncogenic signaling axis, that could modulate invasion and cellular phenotypic plasticity in ATRT-TYR/MYC, via regulation of neural stemness and epithelial-mesenchymal transition (EMT) marker expression.

**Conclusions:**

Our collective study findings point to an important role for PDGFRB-MIF-CD44 signaling in the maintenance of malignant cellular phenotypes in ATRT-TYR/MYC cell lines.

## 1 Introduction

ATRTs are classified as grade IV brain tumors by the World Health Organization (1), and represent only 3-5% of pediatric CNS tumors (2, 3). ATRT is highly malignant, clinically complex (4, 5), and fast-growing tumor (3), that metastasizes in 30-50% of cases (4). There is no definitive treatment for ATRT, and the 5-year survival rate is less than 35%. This makes ATRT one of the most lethal pediatric tumors (6). ATRT has a low somatic mutational burden compared to other pediatric tumors (7). It is characterized by bi-allelic loss of *SMARCB1*, a gene located on chromosome 22 (8), or rarely loss of *SMARCA4*, a gene located on chromosome 19 (9).

The SMARCB1 protein is a component of the multi-protein SWI/SNF, ATP-dependent chromatin remodeling complex (10). *SMARCB1* loss in ATRT has been associated with an altered chromatin structure characterized by increased H3K27me3 repressive marks, which impairs cell differentiation and represses tumor suppressor gene expression (11). DNA methylation analysis identified three epigenetic subgroups of ATRT: Sonic Hedgehog (SHH), Tyrosinase (TYR), and MYC. Notably, ATRT-SHH has neurogenic features, while ATRT-TYR/MYC have varying degrees of mesenchymal features (12–14).

Previous studies showed that cells from mesenchymal ATRT-TYR/MYC subgroup are sensitive to RTKIs (13, 15–17). Several RTKs could be important in ATRT, including PDGFRA, PDGFRB, ErbB2, and FGFR1 (13, 15, 17). Nevertheless, one RTK stands out as being the most differentially expressed between ATRT-TYR/MYC and ATRT-SHH subgroups, with a higher expression in TYR/MYC tumors. This is the platelet-derived growth factor B (PDGFRB) (13). Torchia et al. (13) found phosphorylation of PDGFRB in ATRT primary samples. In addition, ATAC-seq analyses on primary ATRT show that ATRT-MYC tumors have an open chromatin configuration at the *PDGFRB* promoter, but not in primary SHH samples (13). Together, these findings underscore potential importance of PDGFRB in ATRT; however, the mechanism responsible for its activation and its cellular effects on ATRT remain unexplored.

PDGFRB is an RTK that can function as a homodimeric PDGFRB/B or heterodimeric PDGFRA/B receptor complexes (18). PDGFR ligands are PDGF-A, -B, -C, and -D (18). Among the downstream effects of PDGF signaling pathway are cell migration, proliferation, differentiation, and survival (18). PDGF can mediate signaling via both paracrine and autocrine modes (19). The physiologically normal and dominant fate of activated PDGFRB is degradation, proteasome or lysosome-mediated (20, 21). PDGFRB internalization is dependent on ubiquitination by E3 ubiquitin ligases, namely the Casitas b-lineage lymphoma (Cbl) family of E3 ubiquitin ligases: c-Cbl (*CBL*) and Cbl-b (*CBLB*) (20, 21). Under malignant conditions, such as loss-of-function mutations in phosphatases responsible for PDGFR dephosphorylation, PDGFR could be sorted from the early endosome back to the plasma membrane, a process known as PDGFR recycling, which is dependent on Protein Kinase C alpha (PKCα) (22, 23).

The PDGF pathway is essential for normal functioning, including angiogenesis and tissue repair (24, 25). However, dysregulation of the PDGF pathway has been connected to tumorigenesis in several cancer types (24). This includes PDGFR amplification in glioblastoma, activating point mutations in the gastrointestinal stromal tumor, and translocation in chronic myelomonocytic leukemia (24). Oncogenic dysregulation of the PDGF pathway can manifest through mutational activation of PDGFR, ligand-independent activation of PDGFR, or constitutive expression of PDGF ligands (24). Autocrine PDGF signaling has been found to promote the growth and proliferation of tumor cells in leukemia and high-grade glioma (19). Targeting PDGFR has been of interest in cancer therapy, primarily using PDGFR antagonists, which prolonged remission and sensitized tumors to chemotherapy (26).

While the PDGF pathway has been explored in several tumor types, a similar effort has yet to be made in ATRT. RTKIs, such as dasatinib, are fairly well characterized at a mechanistic level and with respect to safety profiles (27). Exploiting this therapeutic sensitivity is of interest in ATRT, as it offers a safer and more efficient treatment option for ATRT-TYR/MYC patients than the widely used chemotherapy/radiotherapy approach that can have long-term side effects on children, including causing brain damage with resulting learning disabilities (28, 29). The absence of an understanding of the role of PDGFRB in ATRT limits the exploitation of this treatment option.

Meanwhile, *SMARCB1* deletions in ATRT-TYR/MYC subgroups have generally been shown to encompass large regions extending beyond the *SMARCB1* gene. These broad deletions have been shown to result in changes in gene expression not only in *SMARCB1*, but also in other genes located near the *SMARCB1* locus on chromosome 22. One specific gene stands out as having the highest expression following a broad *SMARCB1* deletion as indicated by RNA-seq analyses, and this gene is the macrophage migration inhibitory factor (*MIF*) (13, 30). Best known for its role in the adaptive immune response, MIF has emerged as an important cytokine in cancer biology. The MIF signaling pathway has been connected to tumorigenesis in glioblastoma and medulloblastoma, as well as to a dysfunctional anti-tumor immunity in various cancers (31–33). Exceptionally high expression of *MIF* suggests a role for the MIF protein in ATRT pathogenesis. MIF upregulation within ATRT tumor cells and within neighboring cells of the TME remain unexplored.

Several studies highlighted a potential connection between the PDGF and MIF pathways (34, 35). Specifically, a crosstalk between PDGFRB and CD44, MIF’s coreceptor, has been reported in normal and malignant conditions (36, 37). In addition, one specific CD44 isoform, CD44v6, is known to form heteroduplexes with RTKs (VEGFR2, c-MET, and EGFR) enhancing their activation (38, 39).

Using ATRT-TYR/MYC cell lines BT12 and BT16, and their genetically edited sublines, we investigated how disruption of the PDGF signaling pathway impacts ATRT cancer-associated cellular phenotypes. Moreover, we investigated whether there is a connection between the PDGF and MIF pathways, potentially through CD44, that could impact ATRT malignancy.

## 2 Materials and methods

### 2.1 Cell culture

ATRT cell lines BT12 and BT16 were cultured in RPMI-1640 (Wisent, Cat#350–000 RL) supplemented with 10% FBS (Wisent, Cat#090-150- FBS). ATRT cell lines CHLA02, CHLA04, CHLA05, and CHLA06 were cultured in DMEM-F12 (Wisent, Cat# 319–085 CL) supplemented with 20 ng/mL FGF (R&D Systems, Cat#,233-FB), 20 ng/mL EGF (Stem Cell Technologies, Cat#78006) and 1xB27 supplement (Gibco, Cat#17504044). Renal malignant rhabdoid tumor (MRT) cell line G401 was cultured in McCoy’s 5A medium (Gibco, Cat# 16600082) supplemented with 20% FBS (Wisent, Cat#090-150-FBS). Normal human astrocytes (NHA), NIH-3T3, and Human embryonic kidney (HEK)293 cells were cultured in DMEM (Wisent, Cat# 319–205 CL) supplemented with 20% FBS (Wisent, Cat#090-150- FBS). Cells were tested for mycoplasma contamination using a Mycoplasma PCR Detection Kit (Applied Biological Materials, Cat#G238) per the manufacture’s protocol.

Basal expression analysis was performed across multiple cell lines (BT12, BT16, CHLA06, CHLA02, CHLA04, and CHLA05); BT12 and BT16 were selected for subsequent experiments as they had robust expression of pathway components of interest (PDGFRB, MIF, and CD44), are among the most extensively characterized ATRT cell lines in the literature (12), retain certain molecular characteristics consistent with patient-derived tumor data (13), and demonstrated efficient lipofectamine-based transfection suitable for CRISPR/Cas9 editing experiments. (See **Supplementary Table 1** for details about ATRT cell lines).

### 2.2 Western blotting

For all protein extractions, cells were grown in Opti-MEM serum-reduced medium (Gibco, Cat#31985062) overnight to minimize interference of serum or growth factors with PDGF pathway studies. Cells were detached using Versene dissociation reagent (Gibco, Cat#15040066). Whole-cell lysates were prepared using extraction buffer C (EBC) lysis buffer (pH=8.0, Tris-HCL, NaCl, NP-40) supplemented with 1x protease and phosphatase inhibitor cocktail (Thermo Fisher, Cat#78440). Protein concentration was determined using a BCA protein assay (Thermo Scientific, Cat# 23225). For each sample, 25-120 μg/lane of protein, was loaded and separated on a 6-18% SDS-PAGE gel, depending on the abundance and size of protein of interest. Membranes were then developed with SuperSignal West Pico PLUS Chemiluminescent Substrate (Thermo Scientific, Cat#34580) and imaged using a Li-Cor Odyssey imager. When applicable, blots were stripped using Restore PLUS Western Blot Stripping Buffer per manufacturer’s protocol (Thermo Scientific, Cat#46430). Densitometry of blots, when applicable, was performed using ImageJ (https://imagej.net/ij/). Antibodies that were used for immunoblotting are available in **Supplementary Table 3**. The number of technical replicates per biological replicate was one. (See **Supplementary Figure 5** for CD44 antibodies isoform-specificity validation). Original western blot images provided in **Supplementary File 2** (ZIP file).

### 2.3 PDGF stimulation

Cells were serum-starved (BT12 and BT16) or growth factor-deprived (CHLA05, and CHLA06) by growing them in Opti-MEM serum-reduced medium (Gibco, Cat#31985062) overnight (approximately 16 hours). Cells were then stimulated with 10–50 ng/mL PDGF-BB (PeproTech #100-14B), PDGF-CC, or PDGF-DD (StemCell Technologies, Cat#78168, 78222), for 5 minutes to 1 hour, depending on the experiment. PDGFRB phosphorylation was assessed following ligand stimulation at a single optimized timepoint. Stimulation duration was optimized by testing timepoints between 5 and 15 minutes (data not shown); 5 minutes was selected for all subsequent experiments. Receptor recycling and degradation were assessed following ligand stimulation at a single optimized timepoint. Stimulation duration was optimized by testing timepoints of 30, 45, and 60 minutes (data not shown); 60 minutes was selected for all subsequent experiments.

### 2.4 RT-qPCR

Total RNA was extracted using a PuroSPIN Total RNA Purification Kit (Luna Nanotech Cat#NK051-50). cDNA synthesis and qPCR were performed using the Luna Universal One-Step RT-qPCR Kit (New England Biolabs, Cat#E3005). The ΔCq or ΔΔCq method was used to calculate mRNA abundance. Unless otherwise noted, qPCR primers were designed using the Integrated DNA Technologies (IDT) PCR & qPCR primer design tool (https://www.idtdna.com/pages/tools/primerquest). Primer specificity was confirmed *in silico* using the UCSC *In-Silico* PCR online tool (https://genome.ucsc.edu/cgi-bin/hgPcr). The number of technical replicates per biological replicate was 2-3. A list of qRT-PCR primers is available in **Supplementary Table 4**.

### 2.5 siRNA-mediated knockdown

To knockdown *PDGFB*, *CBL*, and *PRKCA*, siRNAs targeting each gene were used from Horizon Discovery, Dharmacon ON-TARGETplus Human siRNA: J-011749-05-0002; J-003004-09-0002; and J-003523-16-0002, respectively. To knockdown *CBLB*, siRNA targeting *CBLB* from Thermo Scientific (Cat# AM16708) was used. siRNAs were transfected at a concentration of 50–100 nM/transfection using Lipofectamine 3000 transfection reagent (Invitrogen, CAT#L3000001). Non-targeting siRNA (Horizon Discovery, ON-TARGETplus Non-targeting Control siRNAs, D-001810-01-05) was used at 50–100 nM/transfection. Transfection was performed according to manufacturer’s protocol in a 6-cm dish format. Cells were serum starved overnight before transfection and harvested 48–72 hours post-transfection for protein or RNA extraction.

### 2.6 Proteasome and lysosome inhibition

Cells were grown in Opti-MEM serum-reduced medium (Gibco, Cat#31985062) overnight (approximately 16 hours), then treated with either 1-10 µM MG132 (Selleckchem, Cat#S2619), 10 µM Bafilomycin A1 (Selleckchem, Cat#S1413), 100 µg/mL cycloheximide (Selleckchem, Cat#S7418), or 0.01-0.1% DMSO (Sigma-Aldrich, Cat#D2650) diluted in Opti-MEM for 2–16 hours. Following treatment, cells were collected for protein extraction.

### 2.7 Cell cycle analysis

Cell cycle analysis was performed on fixed and stained BT12 and BT16 KO cells, derived from the same passage (P15). Briefly, cells were detached using Versene dissociation reagent (Gibco, Cat#15040066). Two million cells were then washed twice with PBS (Wisent, Cat#311–013 CL), filtered through a 40 μm cell strainer (Fisher Scientific, Cat#22-363-547), and fixed with ice-cold 80% ethanol overnight at -20°C. Following fixation, cells were washed with PBS twice, then treated with 500 μL of 200 μg/mL RNase A/DNAse and protease-free (Thermo Scientific, Cat#EN0531) diluted in PBS for 1 hour at 37⁰C. Next, 100 µg/mL Propidium Iodide (PI) (Thermo Scientific, Cat#P3566) diluted in 500 µL PBS was added, to a final concentration of 50 µg/mL PI. Cells were filtered with a 40 μm cell strainer and stained overnight at 4°C. The flow cytometry runs were carried out by Eve Coulter at SickKids-UHN Flow Cytometry Core Facility. Cells were gated to exclude debris and doublets, and cell cycle phases distribution analysis was performed using https://floreada.io/. The number of technical replicates per biological replicate was one. (See **Supplementary Figure 6** for an example of gating steps).

### 2.8 CRISPR/Cas9-mediated generation of stable knockout cell lines

Single-guide RNAs (sgRNA) against genes of interest were designed using the IDT CRISPR Cas9 guide RNA design checker (https://www.idtdna.com/site/order/designtool/index/CRISPR_SEQUENCE). A list of optimized and selected gRNAs used in the successful transfections is provided in **Supplementary Table 5** (including on-target and off-target scores).

gRNAs were cloned into the pSpCas9(BB)-2A-Puro (PX459) V2.0 vector (Addgene, Cat#62988) according to the Zhang lab target sequence cloning protocol (40). Successful cloning was confirmed by Sanger sequencing, using U6 forward primer 5’-GAG GGC CTA TTT CCC ATG ATT CC-3’ immediately upstream of the cloning site. Sequencing was performed by the Centre for Applied Genomics at SickKids.

BT12 and BT16 cells were maintained in RPMI-1640 (Wisent, Cat#350–000 RL) supplemented with 10% FBS (Wisent, Cat#090-150- FBS). Cells (P10) were seeded in 6-well plates and transfected at 70-80% confluency. Prior to transfection, cells were serum-starved for 2–16 hours. Lipofectamine 3000 transfection reagent (Invitrogen, CAT#L3000001) was used according to the manufacturer’s protocol. Seventy-two hours post-transfection, cell culture medium was replaced with fresh medium containing 5 μg/mL of Puromycin (Gibco, Cat# A1113803) for three weeks. Following selection, cells were maintained in 10% FBS RPMI-1640 containing 1-5 μg/mL Puromycin. Pooled cell populations were expanded without clonal selection. All subsequent experiments were therefore performed using heterogeneous pooled KO populations rather than isogenic clonal lines.

Genomic DNA was extracted from cells using a Genomic DNA Mini Kit (Geneaid, Cat#GB300). The regions flanking gRNA target sites were amplified by PCR (New England Biolabs, Cat#M0494), using primers listed in **Supplementary Table 6**. Primers were designed using the Primer Wizard tool (Benchling.com online platform).

PCR products were also subjected to T7 Endonuclease I assays per manufacturer’s protocol (New England Biolabs, Cat#M0302). PCR products were sequenced using Sanger sequencing. Results were analyzed using the TIDE web tool (https://tide.nki.nl/) to assess insertion/deletion (indels) distribution and frequency. Protein lysates from KO cells were analyzed by western blot to confirm the loss of protein expression. For KO validation data, please see **Supplementary Figures 1**-**4** and **Supplementary Table 2**.

#### 2.8.1 CD44 isoform-specific KO generation

To generate CD44s KO, a gRNA against exon 3 of CD44 gene was used. CD44v6 KO was generated as described in Lobo, S. et al. (2020) (41). CD44v6 KO was validated by PCR size reduction, TIDE analysis, and western blot confirmation of CD44v6 protein loss (see **Supplementary Figures 2P–S**, **4P–S**). Zygosity of the deletion was not determined.

### 2.9 Cell invasion/migration assay

BT12 and BT16 cells were serum-starved overnight prior to seeding of 50,000 cells/chamber on Corning FluoroBlok 24-Multiwell Insert Systems PET Membranes (Corning Life Sciences, Cat# 351152) in 400 μL Opti-MEM on the apical side. RPMI-1640 supplemented with 30%FBS was used as chemoattractant for migration assays. For invasion assay, the PET membranes were pre-coated with 100 μL of 200 μg/mL Matrigel (Corning Life Sciences, Cat#354234) for 2 hours at 37 °C prior to the assay. Cells were incubated for 24 hours at 37 °C. Inserts were labeled using 10 μM Calcein AM (diluted in PBS/DMSO) (Thermo Scientific, Cat#C1430) for 1 hour at 37 °C. Fluorescence was then measured using a SpectraMax Gemini EM fluorescence plate reader (Ex/Em 494/517nm). The number of technical replicates per biological replicate was two. (See **Supplementary Figure 9** for a representative validation of the invasion/migration assay using NIH/3T3 cells and **Supplementary Table 7** for RFU values).

### 2.10 Colony formation assay

BT12 and BT16 cells were seeded at 500 cells/well in a 6-well plate and grown for about 2 weeks (or until ~300 colonies were observed in wells under NT gRNA control conditions), with regular medium change every 3–4 days. Cells were washed with PBS, fixed in 1:7 acetic acid:methanol (Fisher Scientific, Cat#A38-500, #A412-4) for 5 minutes, then stained for 1 hour with 0.5% crystal violet (BioShop, Cat#CRY422). Plates were washed 5 times with water, then air-dried for 24–48 hours. Plates were then imaged and colonies counted using the Optronix GelCount Colony Counter. The number of technical replicates per biological replicate was three.

### 2.11 Cell proliferation assay

BT12 and BT16 cells were seeded at 2,000 cells/well in 96-well plates in 100 μL/well RPMI-1640 supplemented with 10%FBS. On days of fluorescence measurements, media was changed to 100 μL OptiMEM/well, prior to adding 10 μL AlamarBlue (Invitrogen, Cat#DAL1025). Cells were incubated for 4 hours at 37 °C, then fluorescence measured using a SpectraMax Gemini EM fluorescence plate reader (Ex/Em 570/585nm). The number of technical replicates per biological replicate was 4-8.

### 2.12 Statistical analysis

All experiments were performed with at least three independent biological replicates, unless otherwise noted. A two-tailed Student’s t-test or a One-way ANOVA (Dunnett’s correction for multiple comparisons) test was used for statistical analysis. The significance cut-off was *p* < 0.05. See Supplementary File 3 (Excel file) for detailed statistical analysis.

## 3 Results

### 3.1 PDGF receptors and ligands are differentially enriched in ATRT cell lines

**Figure 1:**
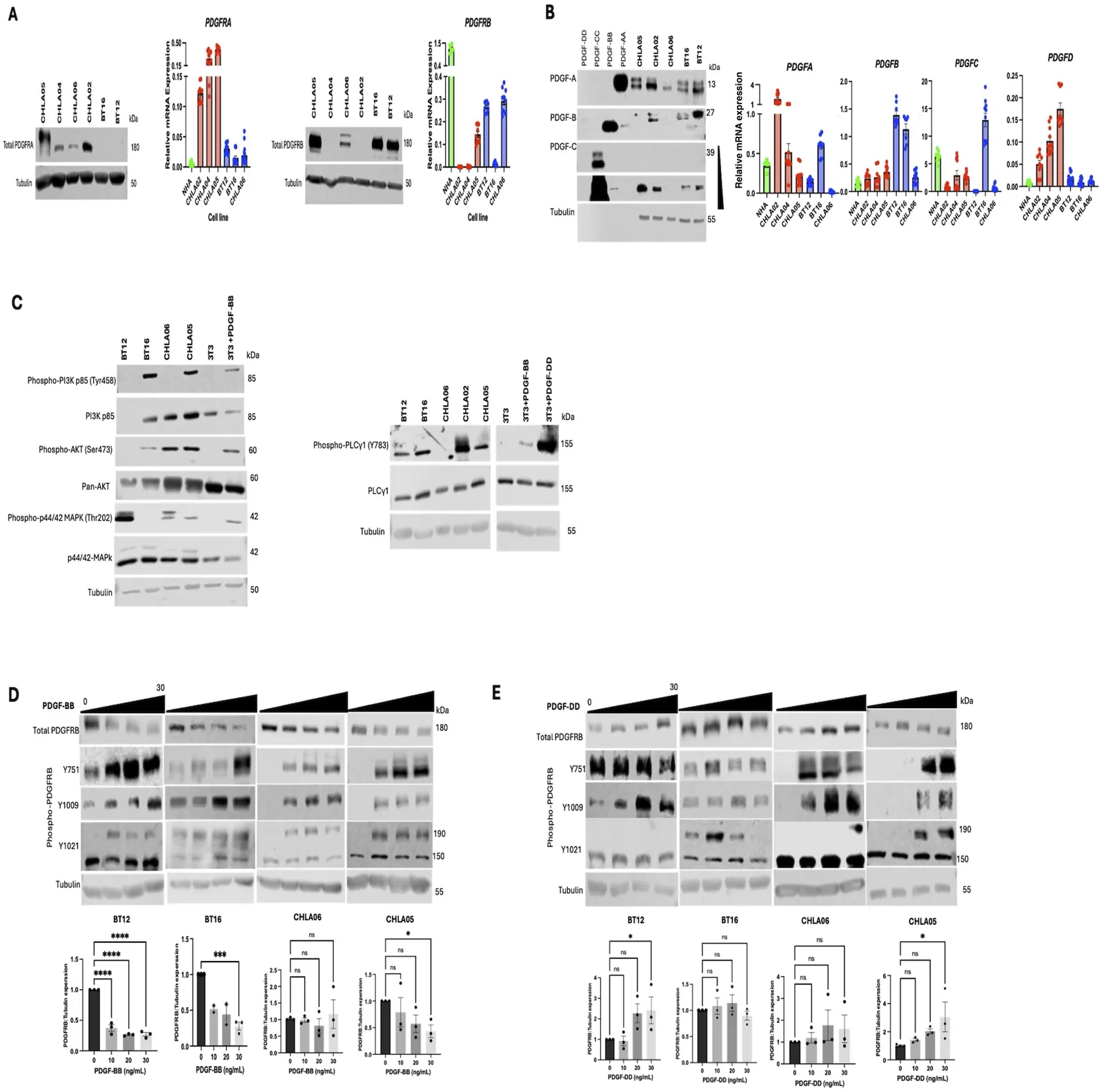
PDGFRB is constitutively active in BT12 and BT16 cells. **(A)** Expression of PDGFRA (left) and PDGFRB (right) protein (by western blot) and mRNA (by qRT-PCR), in ATRT cell lines. ATRT-SHH cell lines are labeled in red; ATRT-TYR/MYC cell lines are labeled in blue; Normal human astrocytes (NHA), labeled in green, is a non-cancer cell line that was included as a negative control for *PDGFRA* and a positive control for *PDGFRB* expression in qRT-PCR. **(B)** Left: Expression of PDGF ligand protein in ATRT cell lines. Human recombinant PDGF ligands were included as a positive control for western blot (10 ng per lane). Right: *PDGFA, PDGFB, PDGFC*, and *PDGFD* mRNA expression (by qRT-PCR in ATRT) cell lines. NHA mRNA was included for comparison. Western blot shown (in **A, B**) is representative of 3 independent biological replicates performed in technical singlets; quantification across replicates was not performed. qRT-PCR data represent the mean ± SEM of n=3 independent biological replicates performed in technical duplicates or triplicates, with a minimum of 6 data points shown per condition. **(C)** PI3K/Akt, MAPK, and PLCγ protein expression. Stimulated (+PDGF-BB/+PDGF-DD) and non-stimulated NIH-3T3 cells were included as a positive and a negative control for phospho-protein expression. Effects of PDGF-BB **(D)** and PDGF-DD **(E)** (0,10, 20, and 30 ng/mL for 5 minutes) on PDGFRB and phospho-PDGFRB expression in BT12, BT16, CHLA06, and CHLA05 by western blot (top). Corresponding densitometry quantification of total PDGFRB (bottom). Western blot shown is representative of 2–3 independent biological replicates. Error bars represent ± SEM (n=2–3 independent biological replicates performed in technical singlets; 2–3 data points per condition). One-way ANOVA with Dunnett’s *post-hoc* test *p< 0.05, **p < 0.01, ***p < 0.001, ****p < 0.0001.

Basal expression of PDGF receptors and ligands was assessed by western blot and qRT-PCR across ATRT cell lines: ATRT-TYR/MYC (BT12, BT16, and CHLA06) and ATRT-SHH (CHLA02, CHLA04, and CHLA05). PDGFRA expression was elevated in ATRT-SHH relative to ATRT-TYR/MYC lines, with CHLA05 showing the highest *PDGFRA* mRNA levels (**Figure 1A**). Conversely, PDGFRB was preferentially expressed in ATRT-TYR/MYC lines, though CHLA05 also expressed notable levels (**Figure 1A**). ATRT cell lines additionally expressed multiple RTKs, including EGFR and ErbB4, as well as several PDGF ligands (**Figure 1B**; **Supplementary Figure 7A**). Among these, *PDGFA* was highly expressed in CHLA02, while *PDGFB*, the primary PDGFRB ligand binding all PDGF receptors, was enriched in BT12 and BT16 (**Figure 1B**). *PDGFC* and *PDGFD* showed preferential expression in BT16 and ATRT-SHH lines, respectively (**Figure 1B**).

### 3.2 PDGFRB is constitutively active in BT12 and BT16 cells

To characterize PDGF pathway activity in ATRT cell lines, phosphorylation status of PDGFRB was assessed. BT12 and BT16 constitutively expressed phospho-PDGFRB without PDGF stimulation; while CHLA05 and CHLA06 only expressed phospho-PDGFRB with external PDGF treatment (**Figures 1D, E**). Furthermore, all ATRT cell lines tested showed activation of downstream PDGF signaling, including active MAPK, PI3K/Akt, and PLCγ pathways (**Figure 1C**). To better understand the effect of PDGF ligand expression on PDGFRB signaling in ATRT cell lines, cells were treated with three different ligands (PDGF-BB, PDGF-CC, and PDGF-DD). PDGF-BB resulted in diminished PDGFRB expression notably in BT12 and BT16, suggesting that PDGF-BB may promote PDGFRB degradation (**Figure 1D**). Similar to PDGF-BB stimulation, PDGF-CC treatment resulted in decreased PDGFRB expression in BT12 and BT16 cells (**Supplementary Figure 7B**). In contrast, PDGF-DD stimulation was associated with an increase (in BT12 and CHLA05) or no significant change in PDGFRB levels (in BT16 and CHLA06), suggesting it may promote PDGFRB accumulation (**Figure 1E**). In summary, the effect of PDGF on PDGFRB expression and phosphorylation appears to be cell line-dependent and ligand-specific.

### 3.3 PDGF-BB drives an autocrine loop leading to PDGFRB degradation in BT12 and BT16 cells

**Figure 2:**
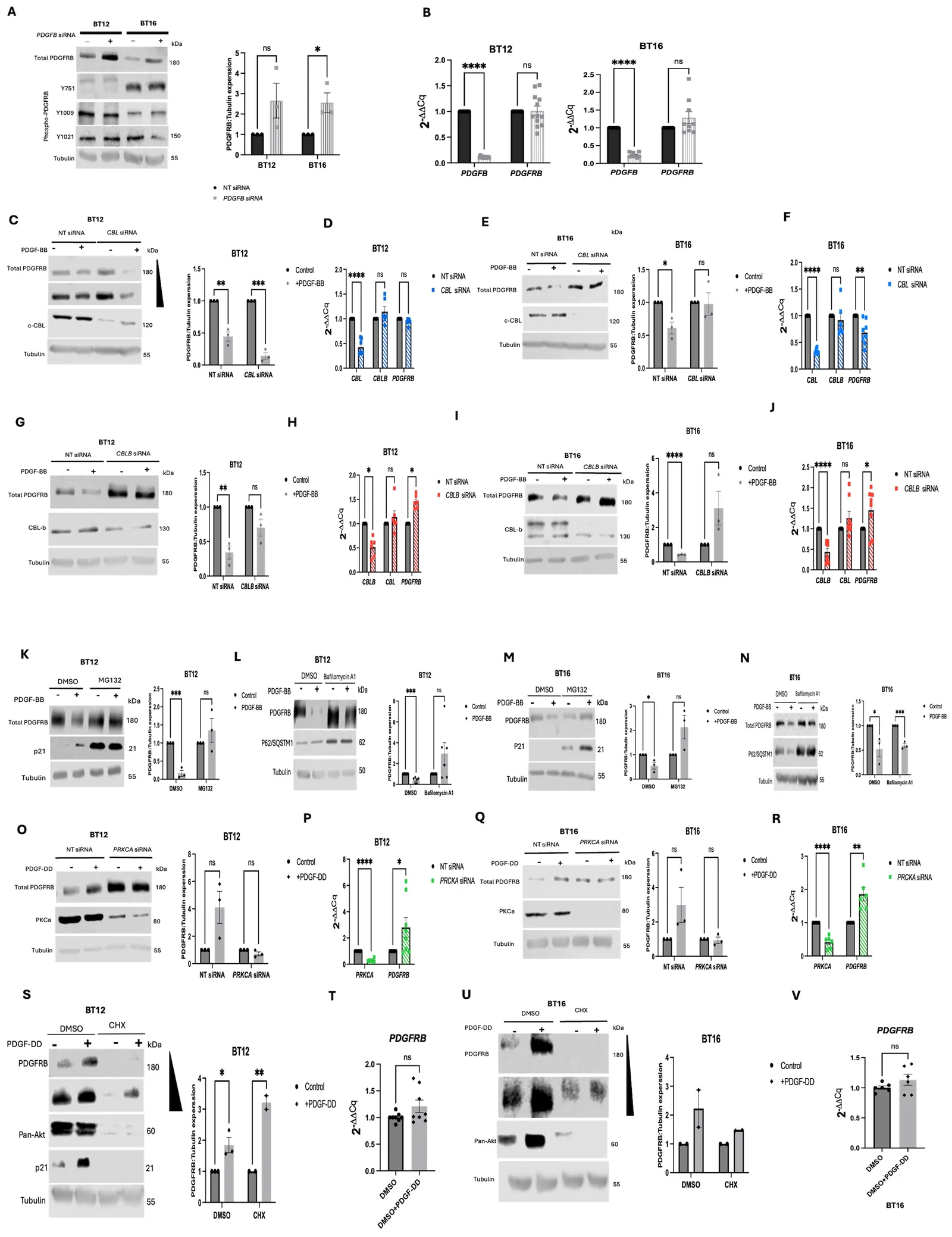
PDGFRB-PDGFB autocrine signaling in BT12 and BT16 cell lines. **(A)** Effects of *PDGFB* knockdown on PDGFRB and phospho-PDGFRB expression in BT12 and BT16 cells by western blot, and corresponding densitometry quantification. *PDGFB* siRNA (–) corresponds to cells transfected with non-targeting (NT) siRNA; *PDGFB* siRNA (+) corresponds to cells transfected with *PDGFB* siRNA. **(B)** qRT-PCR validation of *PDGFB* knockdown efficiency and its effect on *PDGFRB* mRNA levels in BT12 and BT16. Effects of *CBL* knockdown on PDGFRB in BT12 **(C)** and BT16 **(E)** by western blot, and corresponding densitometry quantification. Cells were transfected with NT siRNA or *CBL* siRNA; (+) indicates PDGF-BB stimulation (30 ng/mL for 1 hour) (–); indicates no PDGF-BB stimulation. The gradient on the western blot indicates higher contrast. **(D, F)** qRT-PCR validation of *CBL* knockdown, and its effects on *CBLB* and *PDGFRB* in BT12 **(D)** and BT16 **(F)**. **(G, I)** Effects of *CBLB* knockdown on PDGFRB expression in BT12 **(G)** and BT16 **(I)** by western blot, and corresponding densitometry quantification. Cells were transfected with NT siRNA or *CBLB* siRNA; (+) indicates PDGF-BB stimulation (30 ng/mL for 1 hour) (–); indicates no PDGF-BB stimulation. **(H, J)** qRT-PCR validation of *CBLB* knockdown, and its effects on *CBL* and *PDGFRB* in BT12 **(H)** and BT16 **(J)**. **(K, M)** Effects of MG132 treatment on PDGFRB by western blot, quantified by densitometry in BT12 **(K)** and BT16 **(M)**. Cells were treated with MG132 or with DMSO, followed by PDGF-BB stimulation (30 ng/mL for 1 hour) (+) or no stimulation (–). p21 protein levels were assessed to confirm the efficiency of MG132 treatment. **(L, N)** Effects of Bafilomycin A1 treatment on PDGFRB by western blot, quantified by densitometry in BT12 **(L)** and BT16 **(N)**. Cells were treated with Bafilomycin A1 or with DMSO, followed by PDGF-BB stimulation (30 ng/mL for 1 hour) (+) or no stimulation (–). p62 protein levels were assessed to confirm the efficiency of Bafilomycin A1 treatment. **(O, Q)** Effects of *PRKCA* knockdown on PDGFRB expression in BT12 **(O)** and BT16 **(Q)** cells by western blot, and corresponding densitometry quantification. Cells were transfected with NT siRNA or *PRCKA* siRNA; (+) indicates PDGF-DD stimulation (50 ng/mL for 1 hour) (–); indicates no PDGF-DD stimulation. **(P, R)** qRT-PCR validation of *PRKCA* knockdown, and its effects on *PDGFRB* in BT12 **(P)** and BT16 **(R)**. **(S, U)** PDGFRB protein levels in cycloheximide (CHX)-treated and DMSO-treated BT12 **(S)** and BT16 **(U)** cells by western blot. Cells were pretreated with 100 µg/mL CHX or 0.1% DMSO for 2 hours, followed by PDGF-DD stimulation (50 ng/mL for 1 hour) (+) or no stimulation (–). Right: corresponding densitometry analysis. In BT12, one biological replicate in the CHX condition had ~25.8 relative PDGFRB: Tubulin ratio and was excluded from the analysis using the ROUT method (Q = 1%). Pan-Akt and p21 (short-lived proteins) were assessed to confirm the efficiency of CHX treatment. **(T, V)** *PDGFRB* mRNA levels by qRT-PCR in DMSO-pretreated (0.1% DMSO for 2 hours) BT12 **(T)** and BT16 **(V)** PDGF-DD-stimulated cells (50 ng/mL for 1 hour) relative to non-stimulated cells. Error bars represent ± SEM. Western blot shown is representative of 3 independent biological replicates performed in technical singlets (3 data points per condition, except **(U)**, n=2). qRT-PCR data represent 3 independent biological replicates performed in technical duplicates (minimum 6 data points per condition). Two-tailed Student’s t-test *p< 0.05, **p < 0.01, ***p < 0.001, ****p < 0.0001.

Diminished PDGFRB levels in response to PDGF-BB stimulation, co-expression of PDGF-BB and PDGFRB, as well as constitutively active PDGFRB in BT12 and BT16 cells, suggested PDGFRB-PDGFB autocrine signaling may lead to downregulation and degradation of PDGFRB in ATRTs. Indeed, using siRNA-mediated knockdown, we saw an increase in total PDGFRB protein accumulation, but no change in *PDGFRB* mRNA, in cells transfected with *PDGFB* siRNA (**Figures 2A, B**). Surprisingly, however, there was no considerable change in phospho-PDGFRB levels seen (**Figure 2A**). Together, this indicates that PDGFB can promote degradation of PDGFRB and that PDGFRB phosphorylation in BT12 and BT16 is not solely ligand-dependent.

To confirm the existence of the autocrine loop, we also targeted PDGFRB ubiquitination by knocking down *CBL* and *CBLB*, the two major ubiquitin ligases responsible for PDGFRB ubiquitination. *CBLB* knockdown effects were consistent across both cell lines, where PDGF-BB stimulation failed to cause a decrease in PDGFRB in the *CBLB* knockdown condition (**Figures 2G–J**). This suggests that *CBLB* may be essential for PDGFRB ubiquitination and subsequent degradation. *CBLB* knockdown also caused a significant (*p* = 0.000008; *p* = 0.0284, respectively) increase in *PDGFRB* mRNA in BT12 and BT16 (**Figures 2G–J**). Consequently, the PDGFRB-PDGFB autocrine loop could downregulate PDGFRB protein and mRNA. In BT16 cells, *CBL* knockdown attenuated the effects of PDGF-BB stimulation on PDGFRB, with no significant reduction in PDGFRB levels, in contrast to control condition, where PDGFRB levels were significantly (*p* = 0.0122) reduced post-stimulation (**Figures 2E, F**). However, in BT12 cells, *CBL* knockdown did not affect the ligand-induced decrease in PDGFRB levels indicating these cells could be less dependent on *CBL*-mediated PDGFRB ubiquitination (**Figures 2C, D**).

Next, we tested whether prevention of PDGFRB degradation using MG132, a proteasome inhibitor, and Bafilomycin-A1, a lysosome inhibitor, could disrupt the autocrine loop. MG132 treatment attenuated the PDGF-BB-induced downregulation of PDGFRB in BT12 and BT16, suggesting that proteasome inhibition disrupts autocrine degradation (**Figures 2K, M**). Bafilomycin A-1 treatment was only effective in decreasing PDGFRB degradation in BT12 but not in BT16, suggesting proteasome-mediated degradation is the primary mode of PDGFRB degradation in BT16 cells (**Figures 2L, N**).

Overall, these experiments reveal an autocrine PDGFRB-PDGFB loop in BT12 and BT16 cells that has retained some elements of physiologic control, with negative feedback on PDGFRB protein and mRNA. Taken together, these data indicate that PDGF pathway activation can result from ligand-induced autocrine signaling in BT12 and BT16 cells.

### 3.4 PDGF-DD promotes PKCα-mediated PDGFRB accumulation in BT12 and BT16 cells

**Figure 3:**
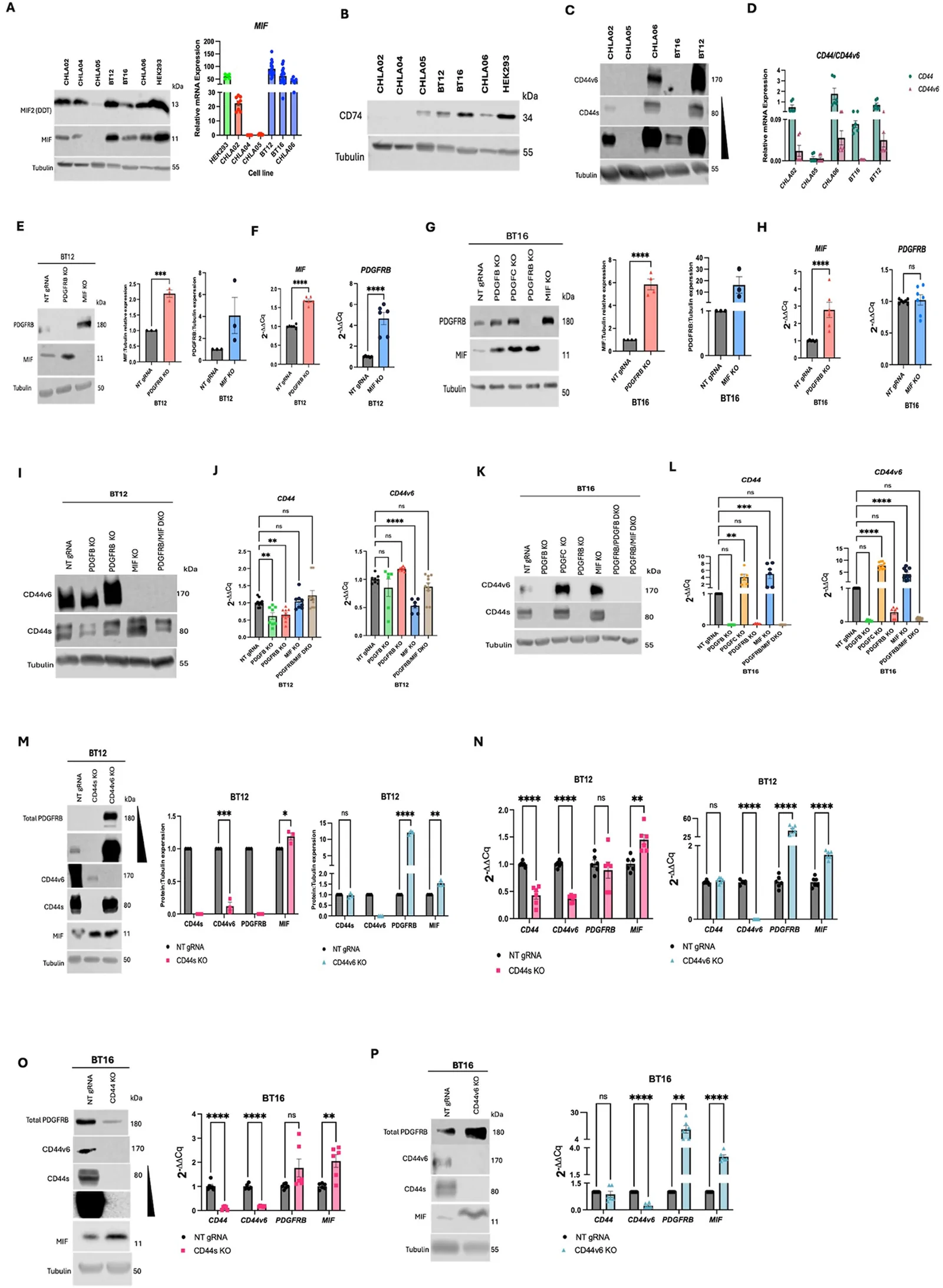
The PDGFRB-CD44-MIF regulatory crosstalk. **(A)** Expression of MIF protein, and its functional homologue MIF2 by western blot (left) and *MIF* mRNA by qRT-PCR (right) in ATRT cell lines. HEK293 was included as a positive control for MIF expression. **(B)** Expression of CD74, MIF’s canonical receptor, by western blot. **(C, D)** Expression of CD44, MIF’s co-receptor, and its isoform CD44v6. For western blot, two different antibodies were used: a standard isoform-specific (CD44s) antibody that does not cross-react with other isoforms; a CD44v6-specific antibody that does not cross-react with CD44s (see **Supplementary Figure 5**). For qRT-PCR, two pairs of primers were used: CD44 primers, which detect all CD44 isoforms, including CD44s and CD44v6 (to our knowledge, it is not possible to design qRT-PCR primers specific to CD44s); CD44v6 primers that detect CD44v6 and CD44v10 isoforms mRNA (see **Supplementary Table 4**). **(E, G)** PDGFRB and MIF protein expression in PDGFRB KO and MIF KO by western blot and corresponding densitometry analysis. **(F, H)** *PDGFRB* mRNA expression in MIF KO, and *MIF* mRNA expression in PDGFRB KO by qRT-PCR. **(I)** Effects of PDGFB KO, PDGFRB KO, MIF KO, and PDGFRB/MIF DKO on CD44s and CD44v6 expression in BT12 by western blot. **(J)** and by qRT-PCR (One-way ANOVA with Dunnett’s *post-hoc* test). **(K, L)** Effects of PDGFB KO, PDGFC KO, PDGFRB KO, MIF KO, and PDGFRB/MIF DKO on CD44s and CD44v6 expression in BT16 by western blot and qRT-PCR (One-way ANOVA with Dunnett’s *post-hoc* test). **(M)** Effects of BT12 CD44s KO and CD44v6 KO on PDGFRB and MIF expression by western blot (and corresponding densitometry), and **(N)** qRT-PCR. **(O, P)** Effects of BT16 CD44s KO and CD44v6 KO on PDGFRB and MIF expression by western blot and qRT-PCR. (Two-tailed Student’s t-test). Western blot shown is representative of 3 independent biological replicates performed in technical singlets (3 data points per condition). qRT-PCR data represent mean ± SEM of 3 independent biological replicates performed in technical duplicates (minimum 6 data points per condition). *p< 0.05, **p < 0.01, ***p < 0.001, ****p < 0.0001.

PDGF-DD stimulation was associated with maintenance or increase of PDGFRB levels despite no change in PDGFRB mRNA (**Figures 2T, V**); suggesting that the effect occurs post-transcriptionally. To determine whether this reflected increased *de novo* protein synthesis, BT12 and BT16 cells were treated with cycloheximide (CHX), which did not abrogate PDGF-DD-induced increase in PDGFRB levels (**Figures 2S, U**); suggesting the increase is not due to translational upregulation. Knockdown of *PRKCA*, which encodes PKCα, essential for PDGFRB recycling, prevented PDGF-DD-induced increase in PDGFRB levels, plausibly implicating receptor recycling in the observed effect (**Figures 2O–R**). In addition, *PRKCA* knockdown caused an increase in *PDGFRB* transcription, suggesting that the cells could compensate for the absence of recycling by producing more PDGFRB receptors (**Figures 3P, R**). Together, these results suggest that PDGF-DD may promote PDGFRB recycling via PKCα, potentially contributing to sustained PDGF signaling.

### 3.5 MIF is highly expressed in ATRT-TYR/MYC cell lines

MIF is the highest expressed gene located near *SMARCB1* in ATRTs with broad *SMARCB1* deletions (13). Interestingly, multiple reports have linked the PDGF and MIF pathways, notably through the intermediate of MIF’s co-receptor CD44, that can also form a complex with PDGFRB (34–37). Given this, we sought to investigate whether PDGF and MIF pathways are connected in ATRT, and whether this connection has any implications for malignancy. All tested lines expressed MIF, except for CHLA05, where MIF protein was below detection limits (**Figure 3A**). ATRT-TYR/MYC cell lines BT12, BT16, and CHLA06 had higher MIF expression relative to ATRT-SHH cell lines, with BT12 having the highest expression (**Figure 3A**). For comparison, MIF expression in these cell lines was similar to or even higher than seen in HEK293, a kidney cell line known to express very high levels of MIF. Additionally, ATRT cell lines also expressed MIF-2 (also known as D-Dopachrome Tautomerase, DDT), a member of the MIF protein family, that is structurally and functionally homologous to MIF, as well as CD74, MIF’s receptor (**Figures 3A, B**).

After confirming MIF expression in ATRT cell lines, we sought to answer the original question about the connection between PDGFRB and MIF. To achieve this, we used stable KOs BT12 and BT16 cells (see **Supplementary Figures 2** and **4** for validation of MIF and CD44 KOs). In BT12 and BT16, we found that PDGFRB KO caused an increase in MIF protein and mRNA levels (**Figures 3E–H**); conversely, MIF KO caused an increase in PDGFRB protein and mRNA expression in BT12 cells, while only PDGFRB protein expression was increased in BT16 cells with MIF KO (**Figures 3E–H**; **Supplementary Figure 7D**). These findings suggest PDGFRB and MIF reciprocally inhibit each other’s expression, potentially as part of a regulatory crosstalk.

### 3.6 PDGFRB and MIF influence CD44 expression

Next, we sought to test whether PDGFRB and MIF signaling impact CD44, which functions on both pathways. Indeed, CD44 is a MIF coreceptor known to form heteroduplexes with PDGFRB. The strongest link between the PDGF and MIF pathways reported in the literature is the PDGFRB-CD44 connection. First, we assessed basal expression of two CD44 isoforms: CD44s which is the ubiquitously expressed isoform, and CD44v6, an isoform known to be upregulated in cancer and to interact with several RTKs. All ATRT cell lines tested (BT12, BT16, CHLA02, and CHLA06) expressed CD44s, except for CHLA05 (**Figures 3C, D**). CD44s expression was highest in BT12 and CHLA06 relative to the other lines. Interestingly, some ATRT cell lines, notably BT12 and CHLA06, expressed high levels of CD44v6, an isoform not expressed in the normal brain (**Figures 3C, D**).

Next, we assessed whether the PDGFRB and MIF regulatory connection described above can influence CD44s and CD44v6 expression in BT12 and BT16 PDGFRB KO and MIF KO cell lines. In both lines, we observed PDGFB and PDGFRB KOs resulted in a substantial reduction of CD44s expression (**Figures 3I–L**). A nearly complete loss of expression was seen in BT16 cells (**Figures 3K, L**). Consequently, PDGFB and PDGFRB may play a role in driving CD44s expression. The effects of PDGFRB KO and MIF KO on CD44v6 expression were more heterogeneous. In BT12, MIF KO led to a significant (*p* < 0.0001) reduction in CD44v6, while PDGFRB KO led to an increase in CD44v6 protein, without a significant change in its mRNA (**Figures 3I, J**). Consequently, MIF can represent an important regulator of CD44v6 expression (**Supplementary Figure 7E**). Surprisingly, in BT16, MIF KO correlated with an increase in both CD44s and CD44v6 (**Figures 3K, L**).

Taking together, these data suggest that PDGFRB and MIF influence CD44 expression in BT12 and BT16. While PDGFRB seems important for driving CD44 transcription in both cell lines, the effect of MIF on CD44 is cell line-dependent, which as a ligand-receptor pair may be subject to differential feedback regulation depending on baseline receptor expression and ligand availability in each cell line.

### 3.7 CD44 influences expression of PDGFRB and MIF

Next, we sought to determine whether CD44 influences expression of PDGFRB and MIF. The observed effects of CD44s and CD44v6 KOs on PDGFRB and MIF were consistent across BT12 and BT16. First, CD44s KO and CD44v6 KO correlated with an increase in MIF (**Figures 3M–P**). Second, CD44s KO led to a reduction in PDGFRB protein, but not mRNA levels (**Figures 3M–O**), indicating that CD44s could promote PDGFRB protein stability. Third, CD44v6 KO caused a significant (*p* < 0.0001; *p* = 0.0057, in BT12 and BT16, respectively) increase in PDGFRB, with almost 25-fold increase in mRNA relative to the NT gRNA control (**Figures 3M, N, P**). Interestingly, in BT12, CD44v6 KO also caused an increase in expression of several other RTKs, including EGFR (**Supplementary Figure 7C**).

In summary, these data indicate that CD44s and CD44v6 influence PDGFRB and MIF expression, and that this effect is consistent across BT12 and BT16. In addition, data show that PDGFRB and MIF influence CD44 expression, with the specific effect being cell line-dependent. Collectively, these data suggest a regulatory PDGFRB-CD44-MIF crosstalk.

### 3.8 The PDGFRB-CD44-MIF crosstalk regulates cell cycle progression and proliferation in BT12 and BT16 cells

The observation that CD44s promotes stability of PDGFRB is an indicator that this crosstalk could well contribute to ATRT malignancy. To further investigate this possibility, we assessed effects of the PDGFRB-CD44-MIF crosstalk on cell proliferation, cell cycle, colony formation, and invasion/migration, using CRISPR/Cas9-mediated knockout (KO) in BT12 and BT16 cell lines.

**Figure 4:**
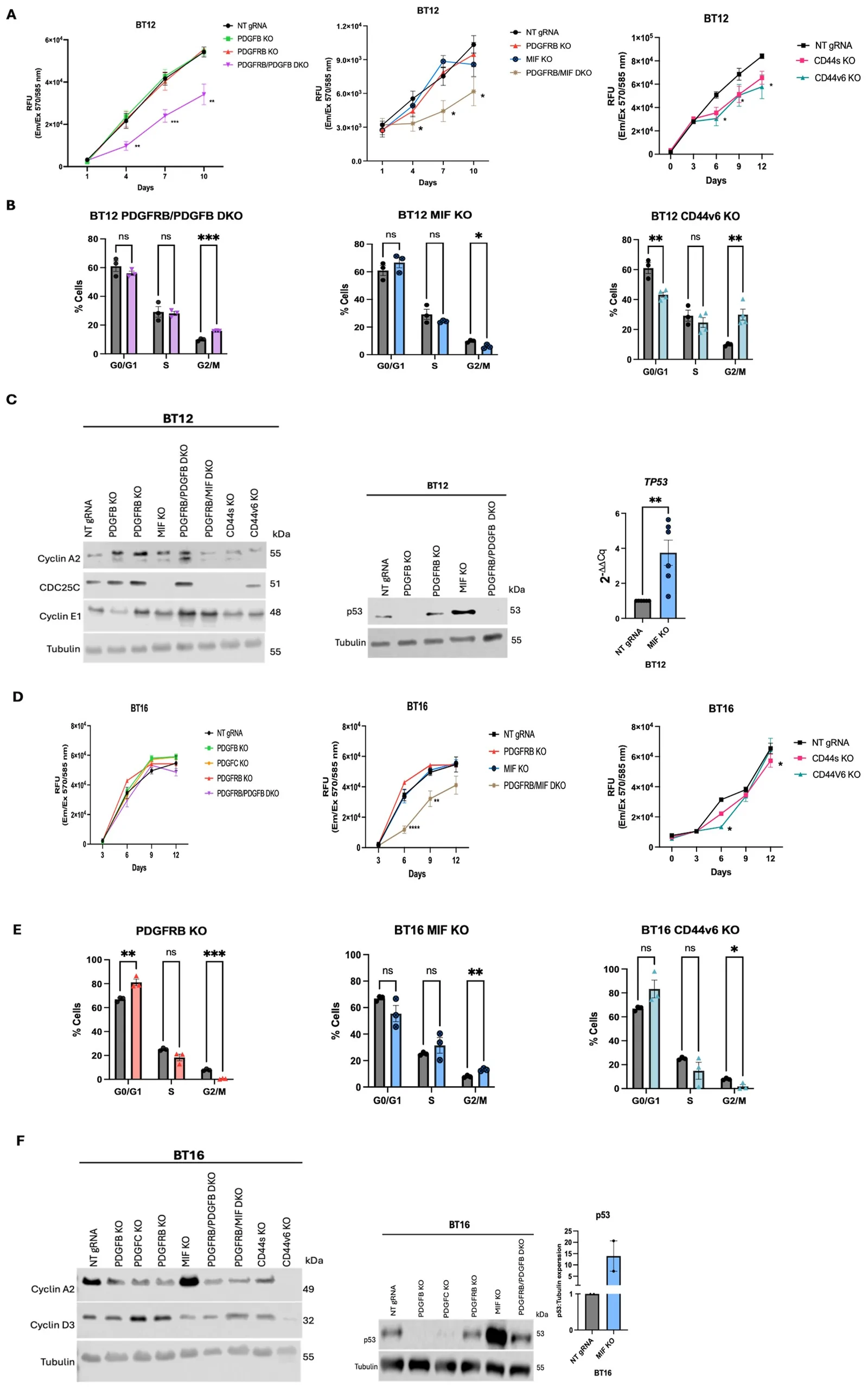
The PDGFRB-CD44-MIF crosstalk promotes cell proliferation in BT12 and BT16 cells. **(A, D)** Cell proliferation in PDGFB KO, PDGFC KO, PDGFRB KO, PDGFRB/PDGFB DKO, MIF KO, PDGFRB/MIF DKO, CD44s KO, and CD44v6 KO relative to NT gRNA in BT12 **(A)** and BT16 **(D)** by measured AlamarBlue assay (Em/Ex 570/585 nm) at indicated timepoints. Cell proliferation data represent mean ± SEM of 3 independent biological replicates performed in technical quadruplicates (12 data points per condition; individual data points omitted for clarity) (One-way ANOVA with Dunnett’s *post-hoc* test). **(B, E)** Cell cycle distribution in PDGFRB KO, PDGFRB/PDGFB DKO, MIF KO, and CD44v6 KO relative to NT gRNA BT12 **(B)** and BT16 **(E)** measured by PI staining by flow cytometry. Cell cycle data represent mean ± SEM of 3 independent biological replicates performed in technical singlets (3 data points per condition) (One-way ANOVA with Dunnett’s *post-hoc* test). **(C, F)** Left: Cell cycle markers protein expression in BT12 **(C)** and BT16 **(F)** NT gRNA, PDGFB KO, PDGFRB KO, MIF KO, PDGFRB/PDGFB DKO, PDGFRB/MIF DKO, CD44s KO, and CD44v6 KO by western blot. Markers were grouped by cell cycle phase/transition. Right: p53 expression in MIF KO relative to NT gRNA. **(C)** qRT-PCR data represent mean ± SEM of 3 independent biological replicates performed in technical duplicates (6 data points) **(F)** Western blot shown is representative of 2 independent biological replicates performed in technical singlets (2 data points) (Two-tailed Student’s t-test).*p< 0.05, **p < 0.01, ***p < 0.001, ****p < 0.0001.

Although either PDGF ligand or receptor KO had no significant effects on BT12 cell growth, PDGFRB/PDGFB double knock-out (DKO) correlated with significantly (*p* = 0.0100; *p* = 0.0004; *p* = 0.0045, on days 4, 7, and 10, respectively) reduced cell proliferation relative to non-targeting gRNA (NT gRNA) controls, indicating that a PDGFRB-PDGFB autocrine loop could be important for cell proliferation (**Figure 4A**). PDGFRB/MIF DKO, CD44s KO, and CD44v6 KO all reduced BT12 proliferation (**Figure 4A**). DKO of PDGFRB and MIF resulted in a robust decrease in cell proliferation, with a significant (*p* = 0.0279) reduction starting as early as day 3 in BT12 PDGFRB/MIF DKO relative to NT gRNA cells (**Figure 4A**). Furthermore, cell cycle analyses showed a modest, but consistent, increase in the percentage of cells in the G2/M phase in BT12 subject to PDGFB KO (15.2±0.5%), PDGFRB KO (15.3±1.8%), and PDGFRB/PDGFB DKO (15.9±0.1%) cells relative to NT gRNA (9.8±0.5%), representing an average 57% increase (**Figure 4B**; **Supplementary Figure 7F**). Consistently, these KO cell lines showed an increase in G2/M protein accumulation (Cyclin A2 and CDC25C) (**Figure 4C**; **Supplementary Figure 7G**). In contrast, BT12 MIF KO (5.8 ± 0.8%) and PDGFRB/MIF DKO (5.4 ± 1.0%) were accompanied by a reduction in the G2/M population (relative to 9.8 ± 0.5% in NT gRNA cells) (**Figure 4B**; **Supplementary Figure 7F**). In addition, MIF KO induced a significant (*p* = 0.0035) increase in *TP53*, a G1/S transition checkpoint activator (**Figure 4C**). KO of CD44v6 in BT12 cells caused a significant (*p* = 0.0043) reduction in the G0/G1 population (43.3 ± 1.6% relative to 60.9 ± 3.6%, which represents ~29% decrease) and increase in the G2/M population (26.4 ± 1.5%; ~2.7-fold increase in the cell population), suggesting G2/M arrest (**Figure 4B**). CD44s KO did not cause a significant change in the cell cycle distribution (**Supplementary Figure 7F**).

In BT16, none of the PDGF KOs significantly affected cell proliferation, while PDGFRB/MIF DKO, CD44s KO, and CD44v6 KO reduced it (**Figure 4D**). Furthermore, PDGFB KO had no effects on the cell cycle in BT16 cells, while both PDGFC KO (6.0±0.2%) and PDGFRB KO (0.5±0.2%) led to a decrease in G2/M population relative to NT gRNA (7.9±0.4%), with the PDGFRB KO also causing an increase in the G0/G1 population (81.1±2.7% relative to 66.9±1.1%) (**Figure 4E**; **Supplementary Figure 7H**). This corresponded to a decrease in Cyclin A2 in PDGFC KO and PDGFRB KO, and an increase in Cyclin D3, a G1 marker, in the same cell lines (**Figure 4F**; **Supplementary Figure 7I**). Interestingly, DKO of PDGFRB/PDGFB in BT16 cells caused a mild increase in the S-phase population (28.0±0.1% relative to 25.1±0.6%) (**Supplementary Figure 7H**). Meanwhile, BT16 MIF KO led to a significant (*p* = 0.0026) increase in G2/M population (13.0 ± 0.5% relative to 7.9 ± 0.4% in NT gRNA cells, which represents ~65% increase); which contrasts with a G2/M decrease seen in PDGFRB KO (**Figure 4E**). Similar to BT12, MIF KO in BT16 also correlated with an increase in p53, despite the phenotype differences in cell cycle distribution between BT12 MIF KO and BT16 MIF KO cells (**Figure 4F**). Interestingly, there were no significant changes in cell cycle distribution in the PDGFRB/MIF DKO (**Supplementary Figure 4H**). BT16 CD44s KO resulted in a significant (*p* = 0.0375) increase in S-phase cells (30.7 ± 1.7% relative to 25.1 ± 0.6%, representing ~22% increase), accompanied by a reduction in the G2/M marker Cyclin A2 (**Figure 4F**; **Supplementary Figure 7H**). BT16 CD44v6 KO showed a significant (*p* = 0.0198) reduction of G2/M cells (1.8 ± 1.5%, representing ~77% decrease), with a simultaneous increase in the G0/G1 population (83.2 ± 7.4% relative to 66.9 ± 1.1%, representing ~25% increase) (**Figure 4E**). The differences between BT12 and BT16 may reflect baseline variation in cell cycle distribution and checkpoint regulatory protein expression.

**Figure 5:**
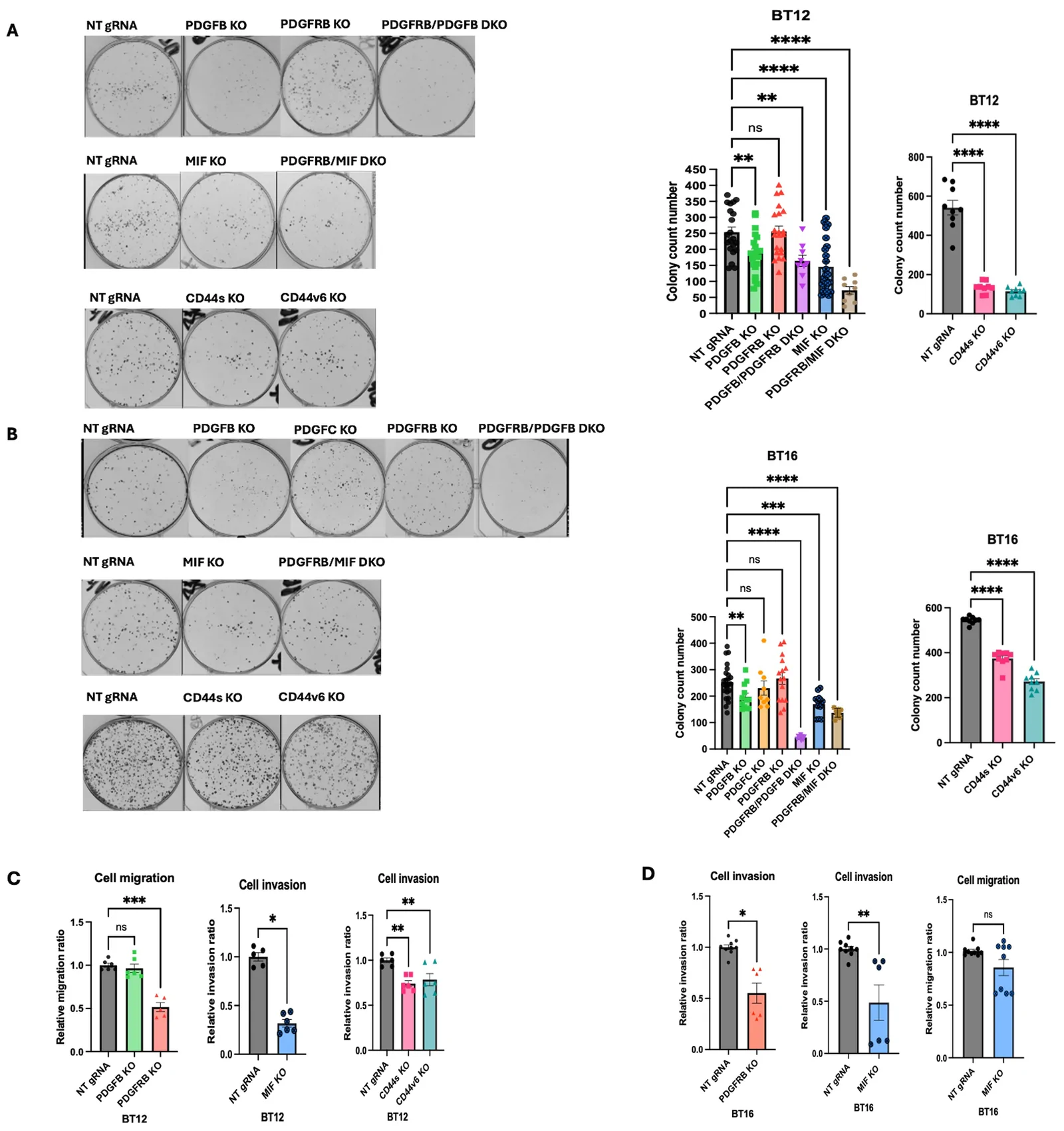
The PDGFRB-CD44-MIF promotes colony formation and cell motility in BT12 and BT16 cells. **(A, B)** Colony count in PDGFB KO, PDGFC KO, PDGFRB KO, PDGFRB/PDGFB DKO, MIF KO, PDGFRB/MIF DKO, CD44s KO, and CD44v6 KO relative to NT gRNA. Colony formation data represent mean ± SEM of 3 independent biological replicates performed in technical triplicates (minimum 9 data points per condition). The images represent one of the replicates (One-way ANOVA with Dunnett’s *post-hoc* test). **(C, D)** Cell invasion/migration in PDGFB KO (BT12), PDGFRB KO, MIF KO, CD44s KO (BT12), and CD44v6 KO (BT12) relative to NT gRNA measured by Matrigel/FluroBlok assay (Em/Ex 494/517 nm). Cell invasion and migration data represent mean ± SEM of 3 independent biological replicates performed in technical duplicates (6 data points per condition; except for BT12 CCD44 KOs migration assay: 2 biological replicates and 4 data points) (**(C)** One-way ANOVA with Dunnett’s *post-hoc* test; **(C, D)** Two-tailed Student’s t-test).*p< 0.05, **p < 0.01, ***p < 0.001, ****p < 0.0001.

We next investigated the effects of PDGFRB-CD44-MIF crosstalk on colony formation as well as cell migration and invasion. We observed that in relation to controls cells with NT gRNA, BT12 and BT16 cells with PDGFB KO, PDGFRB/PDGFB DKO, MIF KO, PDGFRB/MIF DKO, and CD44 KOs, but not PDGFRB KO, exhibited significant (*p* < 0.01) reduction in colony formation, suggesting a predominant ligand-mediated change in BT12 and BT16 clonogenicity (**Figures 5A, B**; **Supplementary Figures 8A, B**). Notably, colony formation in BT16 cells with ligand and receptor DKO was reduced to a much greater extent than that in corresponding BT12 cells (**Figures 5A, B**). Effects on cell migration and invasion showed some variability between BT12 and BT16 cell lines, which may reflect differences in baseline invasive capacity. We found PDGFRB KO caused a significant (*p* = 0.0003) reduction in BT12 cell migration but not invasion (**Figure 5C**; **Supplementary Figure 8C**) while PDGFRB KO reduced (*p* = 0.0245) BT16 cell invasion but not migration (**Figure 5D**; **Supplementary Figure 8C**). In contrast, MIF KO significantly (*p* = 0.0157; *p* = 0.0095, respectively) impaired cell invasion in both BT12 and BT16 (**Figures 5C, D**). Additionally, CD44s KO and CD44v6 KO in BT12 led to a significant (*p* = 0.0011; *p* = 0.0047, respectively) reduction in cell invasion, without affecting cell migration (**Figure 5C**; **Supplementary Figure 8C**).

These observations suggest that KOs in the PDGFRB-CD44-MIF crosstalk components correlated with a decrease in cell proliferation, cell cycle perturbations, reduced clonogenicity, and cell motility.

### 3.9 The PDGFRB-CD44-MIF crosstalk regulates neural stemness and EMT marker expression

Given the effect of KOs in the PDGFRB-CD44-MIF crosstalk components on cellular homeostasis, we tested whether this crosstalk may influence cellular plasticity in ATRT-TYR/MYC. We focused on two major cellular phenotypes that vary across ATRT tumors - neural differentiation and EMT status.

We first investigated the effects of PDGFRB-CD44-MIF crosstalk on neural stemness marker expression in BT12 and BT16 by assessing expression of *SOX2* and *NES*, and early neural differentiation markers (*MAP2* and *TUBB3*) by western blot and qRT-PCR.

**Figure 6:**
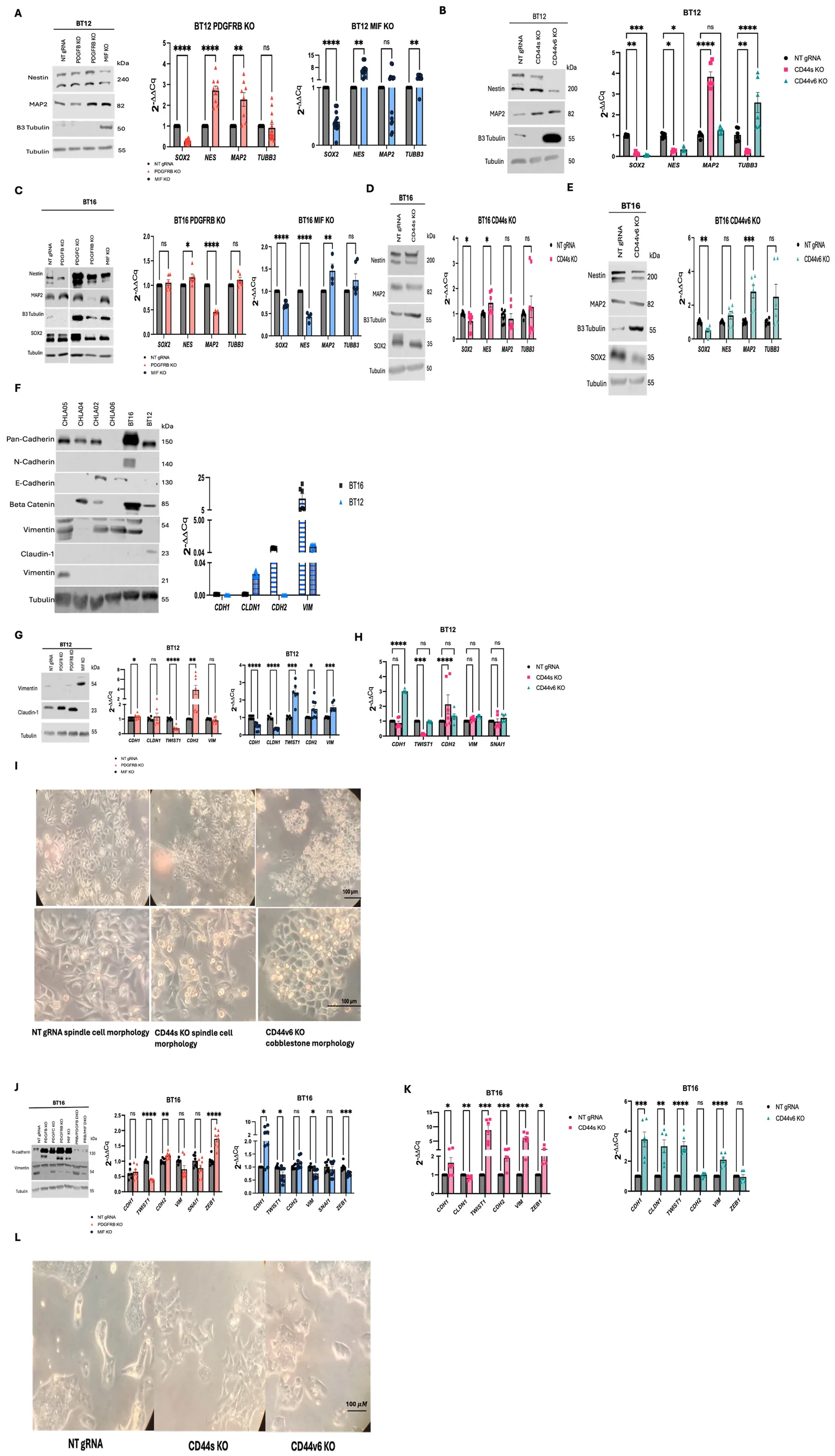
The PDGFRB-CD44-MIF regulates EMT status in BT12 and BT16 cells. **(A–E)** Neural stemness (*SOX2* and *NES*) and differentiation marker (*TUBB3* and *MAP2*) expression in PDGFRB KO, MIF KO, CD44s KO, and CD44v6 KO relative to NT gRNA BT12 **(A, B)** and BT16 **(C–E)** by western blot and qRT-PCR. In BT16, one MIF KO biological replicate values for *MAP2* (131.2 and 131.4) were excluded using the ROUT method (Q = 1%) ((**A, C-E**) Two-tailed Student’s t-test; **(B)** One-way ANOVA with Dunnett’s *post-hoc* test). **(F)** Expression of mesenchymal (N-cadherin, Beta Catenin, Vimentin, and Twist1) and epithelial markers (E-cadherin and Claudin-1) in ATRT cell lines by western blot (left) and by qRT-PCR in BT12 and BT16 cells (right). **(G, J)** EMT marker expression in BT12 **(G)** and BT16 **(J)** PDGFRB KO and MIF KO relative to NT gRNA cells by western blot (left) and qRT-PCR (right). For BT12 western blot, only Vimentin and Claudin-1 had detectable protein expression among the selected markers. For BT16 western blot, only N-cadherin and Vimentin had detectable protein expression among the selected markers (Two-tailed Student’s t-test). **(H, K)** EMT marker expression in BT12 **(H)** and BT16 **(K)** CD44s KO and CD44v6 KO relative to NT gRNA by qRT-PCR. (One-way ANOVA with Dunnett’s *post-hoc* test; **(K)** Two-tailed Student’s t-test). **(I, L)** Representative phase-contrast micrographs of BT12 **(I)** and BT16 **(L)** NT gRNA (left), CD44s KO (center), and CD44v6 KO (right). **(I)** Top panels provide a wide-field view of cell distribution and density at 10x objective magnification. The bottom panels show a high-magnification (20x) view, highlighting individual cell morphology details; **(L)** The panels provide a wide-field view of cell distribution and density at 20x objective magnification. Scale bars represent 100 µm; dimensions estimated based on a mean cell diameter of 12 µm. qRT-PCR data represent mean ± SEM of 3 independent biological replicates performed in technical duplicates (minimum 6 data points per condition). *p< 0.05, **p < 0.01, ***p < 0.001, ****p < 0.0001.

We observed that KOs of PDGFB, PDGFRB, and MIF in BT12 led to a significant (*p* < 0.0001) reduction in *SOX2*, a marker of neural stem cells (**Figure 6A**; **Supplementary Figure 8D**). In addition, PDGFRB KO and MIF KO correlated with a significant (*p* < 0.0001; *p* = 0.0023, respectively) increase in *NES*, which is associated with neural progenitor cells (**Figure 6A**). PDGFRB KO also correlated with an increase (*p* = 0.0060) in *MAP2*, a neuronal lineage commitment marker, while MIF KO induced increased (*p* = 0.0091) expression of *TUBB3*, a neuronal cytoskeleton marker (**Figure 6A**). Similarly, both CD44s KO and CD44v6 KO resulted in a significant (*p* = 0.0136; *p* = 0.0254) reduction in *NES* and *SOX2* (*p* = 0.0013; *p* = 0.0006), with CD44s KO correlating with an increase (*p* < 0.0001) in *MAP2*, and CD44v6 KO correlating with an increase (*p* < 0.0001) in *TUBB3* (**Figure 6B**).

In BT16 KOs of PDGFRB, MIF and, CD44 produced slightly different patterns of neural and stemness markers (**Figure 6C**). PDGFRB KO correlated with increased (*p* = 0.0434) *NES* and reduced (*p* < 0.0001) *MAP2* expression, while MIF KO led to a significant (*p* < 0.0001) reduction in *SOX2* and *NES*, and a significant (*p* = 0.0098) increase in *MAP2* (**Figure 6C**). Both CD44s KO and CD44v6 KO resulted in a reduction in *SOX2*, an increase in *NES*, with CD44v6 KO also causing an increase in *MAP2* (**Figures 6D, E**). These differences seen in BT12 and BT16 KO lines could reflect either differences in PDGF signaling or differences in cell states of the two lines (**Figures 6A, C**).

Overall, results from BT12 and BT16 suggest that PDGFRB-CD44-MIF crosstalk may influence features of stemness and neural plasticity; KOs of components of this axis correlated with changes in neural differentiation indicators, including neural progenitor, neuronal lineage commitment, and neuronal cytoskeleton formation.

### 3.10 Effects of the PDGFRB-CD44-MIF crosstalk on EMT

EMT could be one of the factors driving ATRT inter-tumor heterogeneity. For instance, ATRT-TYR/MYC tumors exhibit mesenchymal features, whereas ATRT-SHH is neurogenic. The mechanism underlying these differences remain unknown. To this end, we assessed effects of PDGFRB-CD44-MIF crosstalk on EMT. We first determined mesenchymal (N-cadherin, CDH2; Beta-catenin; Vimentin, VIM; and Twist-1, TWIST1) and epithelial (E-cadherin, CDH1; and Claudin-1, *CLDN1*) marker expression in ATRT cell lines. BT16 was the only cell line that exhibited a defined mesenchymal phenotype, characterized by expression of N-cadherin, Vimentin, and Beta-catenin, and the absence of E-cadherin and Claudin-1 (**Figure 6F**). The other cell lines had a hybrid epithelial/mesenchymal (EM) phenotype. Notably, BT12, which expressed Claudin-1 and Beta-catenin, lacked detectable expression of other mesenchymal or epithelial markers (**Figure 6F**). CHLA02 and CHLA06 expressed E-cadherin and Vimentin (**Figure 6F**). TWIST-1, a master regulator of EMT, was only detectable at the protein level in CHLA05 (**Figure 6F**). Overall, these data support that EMT status is complex in ATRT, characterized by hybrid expression of both mesenchymal and epithelial markers. Consistent with western blot data, BT16 seems to have a well-defined mesenchymal phenotype, characterized by a high expression of *CDH2* and *VIM* (**Figure 6F**). In contrast, BT12 seems to have a hybrid EM phenotype, with high expression of *CLDN1* and low *CDH2* (**Figure 6F**).

Next, we assessed the effects of genetically manipulating PDGFRB-CD44-MIF crosstalk on EMT status of these cells. In BT12, PDGFRB KO led to a significant (*p* < 0.0001) reduction in TWIST1 and a significant (*p* = 0.0204) increase in CDH1 (**Figure 6G**). Interestingly, PDGFRB KO also correlated with an increase (*p* = 0.0087) in CDH2 (**Figure 6G**). MIF KO in BT12 provoked an opposite effect to the changes induced by PDGFRB KO: it resulted in a decrease (*p* < 0.0001) in epithelial marker expression (CDH1 and CLDN1), with loss of detectable Claudin-1 protein; and it led to increased (*p* = 0.0002; *p* = 0.0201; *p* = 0.0003) accumulation of mesenchymal markers TWIST1, CDH2, and VIM (**Figure 6G**). KO of CD44s KO in BT12 resulted in a significant (*p* = 0.0009) reduction of TWIST1 expression and an increase (*p* < 0.0001) in CDH2, whereas CD44v6 KO led to a significant (*p* < 0.0001) increase in CDH1 (**Figure 6H**; **Supplementary Figure 8E**) and a remarkable change in morphology, transitioning from a spindle cell to a cobblestone morphology, characteristic of epithelial cells (**Figure 6I**).

In BT16, PDGFRB KO led to a reduction (*p* < 0.0001) in TWIST1 and an increase (*p* = 0.0040) in CDH2 (**Figure 6J**). Interestingly, PDGFRB KO also led to an increase *(p* < 0.0001) in ZEB1, a transcription factor involved in EMT. BT16 MIF KO resulted in a significant increase in CDH1, and a reduction in TWIST1, ZEB1, and VIM (*p* = 0.0138; *p* = 0.0001; *p* = 0.0106), which contrasts with the changes observed in BT12 MIF KO (**Figure 6J**). BT16 CD44s KO and CD44v6 KO correlated with a significant (*p* = 0.0005; *p* < 0.0001) increase in TWIST1, with CD44s KO inducing almost a 10-fold increase in TWIST1 mRNA level relative to NT gRNA control (**Figure 6K**). Furthermore, CD44s KO cells appeared to have a spindle-like, elongated morphology, characteristic of mesenchymal cells, and more fibroblastic relative to the NT gRNA (**Figure 6L**). This finding is consistent with the increase in mesenchymal markers observed by qRT-PCR. Whereas CD44v6 KO cells had a rounder, less elongated morphology, indicative of reduced mesenchymal characteristics (**Figure 6L**).

Together, these results indicate that KOs in the PDGFRB-CD44-MIF crosstalk could impact the expression of mesenchymal and epithelial markers, and that the exact effect is gene-specific and cell line-dependent. Baseline expression of EMT-associated markers differed between cell lines (**Figure 6F**), which may contribute to the differential effects of KO conditions on EMT marker expression observed in each line. Notably, across both BT12 and BT16, PDGFRB loss correlated with a decrease in mesenchymal markers.

## 4 Discussion

Previous studies on ATRT established sensitivity to RTKIs, specifically those that inhibit PDGFRB (13, 15–17). However, it remained unclear whether PDGFRB critically contributes to ATRT tumorigenesis, similar to its role in brain tumors with dysregulated PDGF signaling, such as glioblastoma. Our data indicated high-level expression of both PDGFRA and PDGFRB in ATRTs, which differs from other brain tumors where aberrant PDGF pathway activation is generally associated with high expression of only PDGFRA (42). Conversely, PDGFRB expression has been shown to be restricted to stromal cells, such as pericytes (42). Co-expression of PDGFRA and PDGFRB in some ATRT cell lines suggests that they could express heterodimeric PDGFRA-B, which is uncommon for brain tumor cells (23). Our observation of expression of multiple RTKs, including some constitutively active ones, suggest ATRT cell lines heavily depend on RTK signaling. Furthermore, this co-expression could indicate signaling redundancy, where cells could compensate if one signaling pathway is impaired or compromised. Our study also revealed that beyond PDGFs expression in TYR/MYC subgroup cell lines, multiple other RTKs are activated in other ATRT cell lines. CHLA02, for example, is an ATRT-SHH cell line that co-expresses EGFR, ErbB3, and ErbB4, with constitutively active EGFR and ErbB3.

Our study elucidated that the PDGF pathway has two modes of signaling in ATRT. First, autocrine as seen in BT12 and BT16 cells, which induces low-level chronic activation of PDGFRB. This type of signaling exists in other brain tumors, as seen with EGFR autocrine signaling in glioblastoma (43, 44), and has been associated with acquired therapy resistance (45, 46). Notably, our study also revealed all tested ATRT cell lines (BT12, BT16, CHLA05, and CHLA06) exhibited paracrine PDGF signaling, and evidence of a plausible paracrine PDGFD-induced PDGFRB recycling in BT12 and BT16 cells. Indeed, PDGF-DD stimulation in BT12 and BT16 appears to promote PKCα-mediated PDGFRB accumulation, potentially through receptor recycling, a phenomenon that is described for PDGFRA in glioma (47). Thus, use of RTKIs that target PDGFRB, for example, could alleviate a negative feedback loop resulting in enhanced PDGFRB transcription and protein synthesis, and eventually to reactivation of the PDGF pathway in ATRTs. The recycling mechanism represents yet another potential driver of RTKI therapy resistance as receptor recycling could replenish functional PDGFRB to the plasma membrane, overriding the drug’s effect. The proposed autocrine and paracrine signaling models would benefit from additional experimental validation using approaches that provide spatial and temporal resolution of receptor dynamics. Specifically, these findings were assessed at a single optimized timepoint; future time-course analyses would provide more comprehensive insight into receptor degradation kinetics. Moreover, a deeper characterization of these signaling modes *in vivo* is essential for understanding how autocrine or paracrine signaling uncovered in our study may drive RTKI therapy resistance in ATRT.

By using CRISPR/Cas9 stable KO cell lines (PDGFB KO, PDGFRB KO, and PDGFRB/PDGFB DKO), we systematically examined the functional impact of various PDGF signaling pathway components on ATRT cellular phenotypes. Similar to studies of PDGF in other tumors, including glioblastoma (48), our data revealed that PDGF signaling contributes to proliferative signaling in BT12 and BT16. The strongest effect of PDGF signaling was observed on ATRT cell growth in clonogenic assays, suggesting a role in sustaining proliferative potential. In addition to enhanced clonogenicity, our study revealed that PDGFRB is important for cell motility in BT12 and BT16, which is consistent with what was reported for certain other tumors, including mesothelioma (49).

**Figure 7:**
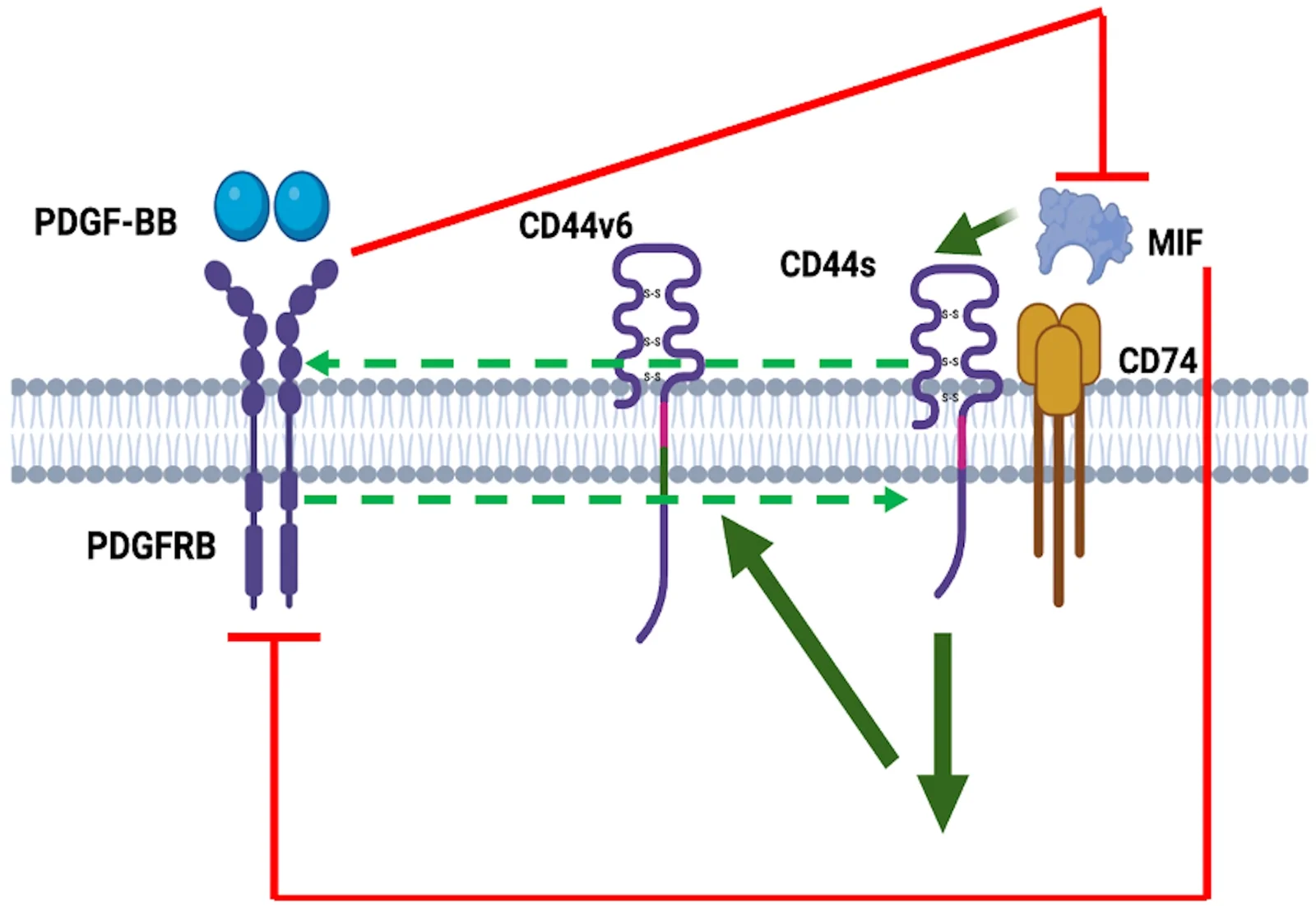
A proposed model of the PDGFRB-CD44-MIF regulatory crosstalk in BT12 and BT16. Red arrows represent the co-inhibitory regulation between PDGFRB and MIF. Green arrows represent positive regulation. This is a hypothesized model and does not represent definitive interactions.

Previous studies indicated that MIF can be upregulated in ATRT, specifically ATRT-TYR/MYC (13). However, the role of MIF in ATRT has remained unknown, and our project has helped to elucidate its functions. MIF is highly expressed in BT12, BT16, and could be connected to the PDGF pathway via the intermediate of CD44 (**Figure 7**). While the precise mechanism by which PDGFRB, CD44, and MIF interact is cell line-dependent and will require further experimentation to define across ATRT subtypes/cell lines, a consistent effect that was shared by both BT12 and BT16 is that PDGFRB appears to drive CD44s expression, which in turn is essential for MIF signal transduction. This further highlights the potential role of PDGFRB as a primary oncogenic driver of this crosstalk. Reciprocally, CD44s could promote PDGFRB protein stability, which is consistent with what was reported about PDGFRB-CD44 interaction in other cancer cells (37, 50). In contrast, CD44v6 loss correlate with increased PDGFRB and MIF expression, which could be a compensatory mechanism. The reciprocal downregulation between PDGFRB and MIF could serve as a regulatory mechanism to maintain balanced expression of CD44v6; very high CD44v6 levels may be toxic to cells, by inducing replication or metabolic stress, as reported in a study of esophageal squamous cell carcinoma (51). Nevertheless, this requires further experimental validation in ATRT. As this proposed PDGFRB-MIF crosstalk is inferred solely from expression changes in individual KO contexts, the role of CD44 as a mediator remains to be more robustly tested. Formal establishment of this relationship would require combinatorial KOs (such as PDGFRB/CD44 DKO) and rescue experiments to distinguish direct mediation from parallel regulatory responses. Moreover, these findings should be interpreted in the context of the pooled non-clonal experimental system employed (for example, the residual CD44v6 isoform expression observed in BT12 CD44s KO). Furthermore, the use of only two cell lines, BT12 and BT16, represents a limitation of the current model, as findings may not fully capture subgroup-specific biology across all ATRT subtypes. Validation in additional cell lines representing ATRT-SHH and primary patient-derived cultures would strengthen the generalizability of this proposed crosstalk.

KOs in the PDGFRB-CD44-MIF crosstalk components significantly impaired cell proliferation, cell cycle, colony formation, and invasion/migration, suggesting that this crosstalk can be malignant. Furthermore, our data suggest that the PDGFRB-CD44-MIF crosstalk regulates cell states in BT12 and BT16 cells. The PDGF pathway could promote EMT in BT12 and BT16 cells, in line with the findings from other tumors (52, 53). PDGFRB appears to drive mesenchymal markers expression, nevertheless, its loss also led to a significant increase in *CDH2*. Although *CDH2* is a mesenchymal marker in carcinomas, brain tumors, such as glioblastoma, commonly maintain high levels of *CDH2* while reversing the mesenchymal phenotype, and rarely express *CDH1 (*54). Consequently, an increase in *CDH2* in PDGFRB KO does not contradict the partial loss of mesenchymal phenotype. MIF plays a distinct role, where it can promote MET in BT12, and EMT in BT16. MIF’s effect on EMT is context dependent: in glioblastoma, where MIF expression is acute and induced by hypoxia, MIF has been found to promote EMT (55); in gastrointestinal cancer, where MIF expression is chronic, it promotes MET, specifically through the CD74 intracellular domain (CD74-ICD), which is formed upon MIF binding (56). The TME could be important in determining whether MIF signaling resembles that of glioblastoma or gastrointestinal tumors, and it may explain differences in MIF’s impact on EMT in BT12 and BT16. Specifically, this may reflect differences in MIF signaling modes, analogous to the distinct PDGF signaling modes previously described in these cells. Like MIF, CD44s also seems to play a dual role, in BT12, it can promote EMT; in BT16, CD44s can inhibit mesenchymal marker gene expression, especially *TWIST1*. One possible explanation is that because BT16 has a pronounced basal mesenchymal phenotype, CD44s could prevent the cells from undergoing further EMT, thereby reducing the risk of acquiring an extreme mesenchymal phenotype. Cells that undergo extreme EMT lose invasive capacities and can even become quiescent (57). Finally, CD44v6 KO stood out for its considerable effect on cell morphology, prompting both BT12 and BT16 to acquire a more epithelial phenotype, and thus CD44v6 loss can be central in driving MET in these cells. However, the markers assessed represent a limited subset of the EMT program, and a more comprehensive characterization incorporating additional regulators as well as brain tumor-relevant mediators including YAP/TAZ and STAT3, would be required to better define EMT status in this context.

Overall, the findings suggest that PDGFRB-CD44-MIF crosstalk is associated with the expression of neural stemness-associated markers and a less differentiated cell state. In the absence of MIF, CD44s, and CD44v6 in BT12 and BT16 cells, a partial neural differentiation is seen. This is consistent with a reported role for CD44 as a stemness marker in cancer cells (58). Though, stemness assessment was limited to a select panel of markers, and a more comprehensive characterization incorporating *in vivo* limiting dilution assays will be needed to determine whether the observed changes in stemness-associated markers correspond to an altered tumor-initiating capacity. Furthermore, a limitation of using pooled, non-clonal KO cell populations rather than isogenic clonal lines is that it introduces heterogeneity in editing efficiency within the population. As a result, observed phenotypes may reflect a mixture of fully edited, partially edited, and potentially unedited cells, which could influence the magnitude of the observed effects. Future studies using validated isogenic clonal lines would strengthen the mechanistic interpretation of the findings presented here. In addition, lack of rescue experiments cannot allow us to exclude the possibility of secondary or generic effects caused by CRISPR/Cas9 gene editing process rather than the specific gene KO. For example, cDNA rescue experiments of PDGFRB, CD44, and MIF would confirm the gene-specificity of our findings.

In summary, the PDGFRB-CD44-MIF crosstalk appears to facilitate cellular maintenance of an optimal EM and stemness phenotype associated with growth and invasion. It should also be noted that EMT and stemness represent highly plastic and reversible cell states that exist along a spectrum rather than as discrete categories. The expression changes observed here could reflect a partial or hybrid state transition, and bulk expression analysis (western blot, qRT-PCR, and RNA-seq) cannot resolve the heterogeneity of cell states within a cell population. Single-cell RNA-seq analysis would be required to deconvolute cellular heterogeneity and to model the dynamic and continuous nature of these cell states.

The observed variability in responses between BT12 and BT16 cell lines highlights both the complexity of ATRT biology and the inherent limitation of drawing broad conclusions from a restricted number of models. Accordingly, the findings presented in this paper should be interpreted as hypothesis-generating observations that provide preliminary evidence for the biological relevance of the studied PDGF signaling modes and PDGFRB-CD44-MIF crosstalk in ATRT, rather than definitive mechanistic conclusions. Indeed, two cell lines are insufficient to fully capture the molecular and phenotypic inter- and intra-tumoral heterogeneity of ATRT. Future studies incorporating additional ATRT cell lines representing all three molecular subgroups, as well as primary patient-derived cultures, would provide a more comprehensive assessment of the study’s findings relevance and generalizability across the heterogeneous ATRT landscape.

## Data Availability

The original contributions presented in the study are included in the article/**Supplementary Material**. Further inquiries can be directed to the corresponding author.

## Glossary

*ACTB*: Actin cytoskeletal beta
*AKT*: AKR Thymoma/Protein kinase B
ANOVA: Analysis of variance
ATAC-seq: Assay for transposase-accessible chromatin with high-throughput sequencing
ATP: Adenosine triphosphate
ATRT-MYC: Atypical teratoid rhabdoid tumor-myelocytomatosis
ATRT-SHH: Atypical teratoid rhabdoid tumor-sonic hedgehog
ATRT-TYR: Atypical teratoid rhabdoid tumor-tyrosinase
ATRT: Atypical teratoid rhabdoid tumor
*BAF47*: Brahma-related gene-1-associated factor 47
BT12: Brain tumor 12
BT16: Brain tumor 16
*CBL*: Casitas B-lineage lymphoma
*CBLB*: Casitas B-lineage lymphoma proto-oncogene B
*CD44*: Cluster of differentiation 44
*CD44s*: Standard cluster of differentiation 44
*CD44v6*: Cluster of differentiation 44 variable exon 6
*CD74*: Cluster of differentiation 74
*CDC*: Cell division cycle
*CDH*: Calcium-dependent adhesion
*CDK*: Cyclin-dependent kinase
*CDKN*: Cyclin-dependent kinase inhibitor
ChIP-seq: Chromatin immunoprecipitation sequencing
CHLA: Children’s Hospital Los Angeles
*CLDN1*: Claudin-1
CNS: Central nervous system
CRISPR/Cas9: Clustered regularly interspaced short palindromic repeats-associated protein 9
*CXCR*: C-X-C Motif chemokine receptor
*DDT*: D-dopachrome tautomerase
DKO: Double knockout
DMSO: Dimethyl sulfoxide
DNA: Deoxyribonucleic acid
EBTs: Embryonal brain tumors
ECM: Extracellular matrix
*EGFR*: Epidermal growth factor receptor
EMT: Epithelial-mesenchymal transition
*ERBB*: Erythroblastic oncogene B receptor
*ESRP*: Epithelial splicing regulatory protein
ETMRs: Embryonal tumors with multilayered rosettes
FACS: Fluorescence-activated cell sorting
FBS: Fetal bovine serum
*FGFR1*: Fibroblast growth factor receptor 1
G0, G1, G2: Gap phase 0, 1, 2
*GAPDH*: Glyceraldehyde-3-phosphate dehydrogenase
GFP: Green fluorescent protein
gRNA: Guide RNA
H3K27me3: Histone H3 lysine 27 trimethylation
HEK293: Human embryonic kidney 293
ICD: Intracellular domain
IHC: Immunohistochemistry
*INI1*: Integrase interactor 1
KO: Knockout
M (as in G2/M): Mitosis
*MAP2*: Microtubule associated protein 2
*MAPK*: Mitogen-activated protein kinase
MET: Mesenchymal-epithelial transition
*MIF*: Macrophage migration inhibitory factor
mRNA: Messenger ribonucleic acid
MT: Mesenchymal transition
*MYC*: Myelocytomatosis gene
*NES*: Nestin
NHA: Normal human astrocytes
NT: Non-targeting
*PDGF*: Platelet-derived growth factor subunit A
*PDGFR*: Platelet-derived growth factor receptor
*PI3K*: Phosphoinositide 3-kinase
PLC-γ: Phospholipase C, gamma 1
PRC2: Polycomb repressive complex 2
*PRKCA*: Protein kinase C alpha
qRT-PCR: Quantitative reverse transcription polymerase chain reaction
*Rab4a*: Ras-related protein 4a
RFU: Relative fluorescence units
RNA-seq: Ribonucleic acid sequencing
RNA: Ribonucleic acid
RTK: Receptor tyrosine kinase
RTKI: Receptor tyrosine kinase inhibitor
scRNA-seq: Single-cell RNA sequencing
SEM: Standard error of the mean
*SMARCA4*: SWI/SNF related, matrix associated, actin dependent regulator of chromatin, subfamily A, member 4
*SMARCB1*: SWI/SNF related, matrix associated, actin dependent regulator of chromatin, subfamily B, member 1
*SNAI1/2*: Snail family transcriptional repressor 1/2
*SNF5*: Sucrose nonfermenting, yeast, factor 5
*SOX2*: SRY-box transcription factor 2
SWI/SNF: SWItch/Sucrose non-fermentable
*TGFB*: Transforming growth factor beta
TIDE: Tracking of insertions and deletions
TME: Tumor microenvironment
*TUBB3*: Tubulin beta class III
*VIM*: Vimentin
WHO: World health organization
*ZEB1/2*: Zinc finger E-box-binding homeobox 1/2.
