# Correction: A PDGFRB-CD44-MIF crosstalk promotes cancer-associated cellular phenotypes in ATRT-TYR/MYC cell lines

**DOI:** 10.3389/fonc.2026.1999768

**Published:** 2026-10-22

**Authors:** Yacine A. Choutri, Liming Xu, Fupan Yao, Annie Huang

**Affiliations:** 1 Department of Medical Biophysics, University of Toronto, Toronto, ON, Canada; 2 Research Program in Cell Biology, Hospital for Sick Children, Toronto, ON, Canada; 3 Arthur and Sonia Labatt Brain Tumour Research Centre, Hospital for Sick Children, Toronto, ON, Canada; 4 SickKids Research Institute, Toronto, ON, Canada

**Keywords:** ATRT, CD44, EMT, MIF, PDGFRB

There was a mistake in **Figure 3** as published. In **Figure 3I**, the band labels on the western blot were swapped, affecting both the protein name and molecular weight: the band corresponding to “CD44v6 170 kDa” was labeled as “CD44s, 80 kDa,” and the band corresponding to “CD44s 80 kDa” was labeled as “CD44v6, 170 kDa.” The original blot images are correctly labeled, and all quantitative analyses and interpretations presented in the article were performed using the correct band identities. This correction is limited to the figure labels in **Figure 3I** only, and does not affect the underlying data, analyses, results, or conclusions of the study. The corrected **Figure 3** appears below.

**Figure 3:**
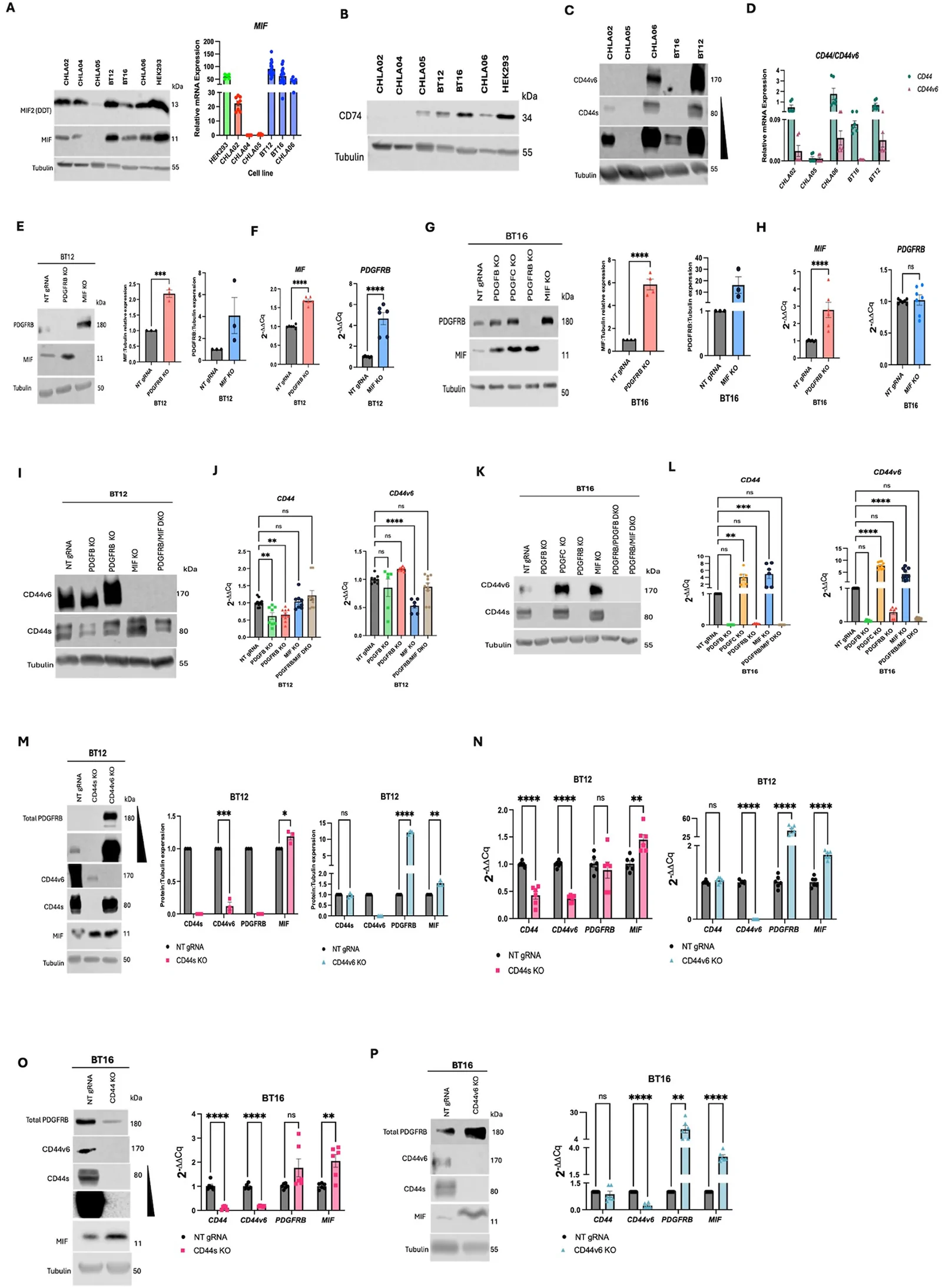
The PDGFRB-CD44-MIF regulatory crosstalk. **(A)** Expression of MIF protein, and its functional homologue MIF2 by western blot (left) and *MIF* mRNA by qRT-PCR (right) in ATRT cell lines. HEK293 was included as a positive control for MIF expression. **(B)** Expression of CD74, MIF’s canonical receptor, by western blot. **(C, D)** Expression of CD44, MIF’s co-receptor, and its isoform CD44v6. For western blot, two different antibodies were used: a standard isoform-specific (CD44s) antibody that does not cross-react with other isoforms; a CD44v6-specific antibody that does not cross-react with CD44s (see Supplementary Figure 5). For qRT-PCR, two pairs of primers were used: CD44 primers, which detect all CD44 isoforms, including CD44s and CD44v6 (to our knowledge, it is not possible to design qRT-PCR primers specific to CD44s); CD44v6 primers that detect CD44v6 and CD44v10 isoforms mRNA (see Supplementary Table 4). **(E, G)** PDGFRB and MIF protein expression in PDGFRB KO and MIF KO by western blot and corresponding densitometry analysis. **(F, H)** *PDGFRB* mRNA expression in MIF KO, and *MIF* mRNA expression in PDGFRB KO by qRT-PCR. **(I)** Effects of PDGFB KO, PDGFRB KO, MIF KO, and PDGFRB/MIF DKO on CD44s and CD44v6 expression in BT12 by western blot. **(J)** and by qRT-PCR (One-way ANOVA with Dunnett’s post-hoc test). **(K, L)** Effects of PDGFB KO, PDGFC KO, PDGFRB KO, MIF KO, and PDGFRB/MIF DKO on CD44s and CD44v6 expression in BT16 by western blot and qRT-PCR (One-way ANOVA with Dunnett’s post-hoc test). **(M)** Effects of BT12 CD44s KO and CD44v6 KO on PDGFRB and MIF expression by western blot (and corresponding densitometry), and **(N)** qRT-PCR. **(O, P)** Effects of BT16 CD44s KO and CD44v6 KO on PDGFRB and MIF expression by western blot and qRT-PCR. (Two-tailed Student’s t-test). Western blot shown is representative of 3 independent biological replicates performed in technical singlets (3 data points per condition). qRT-PCR data represent mean ± SEM of 3 independent biological replicates performed in technical duplicates (minimum 6 data points per condition). *p<0.05, **p<0.01, ***p<0.001, ****p<0.0001.

The original version of this article has been updated.

