# Supplementary material for: A PDGFRB-CD44-MIF crosstalk promotes cancer-associated cellular phenotypes in ATRT-TYR/MYC cell lines
Source: Front Oncol. 2026 Sep 9;16:1921228. doi: 10.3389/fonc.2026.1921228 (PMC13597457; doi:10.3389/fonc.2026.1921228)
Supplement: Supplementary file 1 [file DataSheet1.pdf]

Supplementary Table 1 Details of ATRT cell lines

Molecular details

Cell line	Culture type	Group	Location	*SMARCB1* status
**CHLA-05-ATRT**	**Suspension**	**SHH**	Supratentorial	HMZ Del x6
**CHLA-02-ATRT**	**Suspension**	**SHH**	Infratentorial	HMZ Del x1-9
**CHLA-04-ATRT**	**Suspension**	**SHH**	Supratentorial	HMZp.Gln257X
**CHLA-06-ATRT**	**Suspension**	**TYR/MYC**	Infratentorial	HMZ Del x6
**BT12**	**Adherent**	**TYR/MYC**	Infratentorial	HMZp.Met27Argfs*28
**BT16**	**Adherent**	**TYR/MYC**	Infratentorial	HMZp.Arg60Glufs*10

HMZ Del: homozygous deletion; x: exon; X: stop codon; HMZp: homozygous point mutation; fs: frameshift (Adapted from Torchia *et al*., 2016(1)).

Clinical details

Cell line	Age (Months)	Gender	Derivation	Originally from
**CHLA-05-ATRT**	**34.0**	**Male**	Isolated from the resected brain tumor at the time of diagnosis	Children's Hospital Los Angeles; USA
**CHLA-02-ATRT**	**20.4**	**Male**	Isolated from the resected brain tumor at the time of diagnosis	Children's Hospital Los Angeles; USA
**CHLA-04-ATRT**	**20.0**	**Male**	Isolated from the brain tumor at the time of autopsy	Children's Hospital Los Angeles; USA
**CHLA-06-ATRT**	**3.0**	**Female**	Unknown	Children's Hospital Los Angeles; USA
**BT12**	**24.0**	**Female**	Isolated from the resected brain tumor at the time of diagnosis	Children's Hospital of Philadelphia; USA
**BT16**	**1.5**	**Male**	Unknown	Children's Hospital of Philadelphia; USA

Cell lines clinical details retrieved from https://www.cellosaurus.org/ and https://www.atcc.org/

Supplementary Table 2 KOs Genomic validation results summary

Cell line	Gene targeted	Exon targeted	Indel type	Indel percentage	Total efficiency
BT12	*CD44*	Exon 3	-3bp deletion	93.1%	97%
	MIF	Exon 1	+1bp insertion	66%	96.1%
			-7bp deletion	32.1%	
	PDGFB	Exon 2	+1bp insertion	95.2%	96.1%
	PDGFRB	Exon 4	+1bp insertion	3.4%	93.9%
			+3bp insertion	47%	
			-3bp deletion	1.9%	
			-4bp deletion	41.8%	

	PDGFB (in PDGFRB/PDGFB DKO)	Exon 2	-9bp deletion	60.4%	74.7%
	PDGFRB (in PDGFRB/PDGFB DKO)	Exon 4	+1bp insertion	17.4%	96.5%
			-1bp deletion	73.2%	
			-3bp deletion	1.3%	
	PDGFRB (in PDGFRB/MIF DKO)	Exon 4	+1bp insertion	25.5%	60.7%
			-1bp insertion	21.2%	
	MIF (in PDGFRB/MIF DKO)	Exon 1	+3bp insertion	3.8%	94.3%
			-1bp deletion	85.3%	
BT16	*CD44*	Exon 3	-2bp deletion	37.7%	78%
			-3bp deletion	6.1%	
			-4bp deletion	24%	
	MIF	Exon 1	+2bp insertion	79.1%	93.3%
			+1bp insertion	14.2%	
	PDGFB	Exon 2	-9bp deletion	42.5%	54%
			+1bp insertion	4.3%	
	PDGFC	Exon 4	+1bp insertion	94.1%	96.7%
	PDGFRB	Exon 4	-2bp deletion	45.9%	82.5%
			-1bp deletion	27.4%	
	PDGFB (in PDGFRB/PDGFB DKO)	Exon 2	-8bp deletion	79%	84.8%
	PDGFRB (in PDGFRB/PDGFB DKO)	Exon 4	-7bp deletion	1.4%	72.2%
			-4bp deletion	1.4%	
			+1bp insertion	66.4%	
	PDGFRB (in PDGFRB/MIF DKO)	Exon 4	+1bp insertion	74.5%	75.8%
	MIF (in PDGFRB/MIF DKO)	Exon 1	-10bp deletion	94.4%	95.3%

Supplementary Table 3 Western blotting antibodies list

**Antibody**	**Catalog number**	**Concentration**
**Primary antibodies**		
α-Tubulin (monoclonal mouse)	Cell Signaling Technology (CST) #3873	1:10,000
Beta 3 Tubulin (monoclonal rabbit)	CST#5568	1:1,000
c-CBL (monoclonal rabbit)	CST#2179	1:1,000
Cas9 (*S. pyogenes*) (monoclonal rabbit)	CST#65832	1:1,000
CBL-b (polyclonal rabbit)	Bethyl laboratories # A302-903A	1:1,000

CD44 (monoclonal rabbit)	CST #37259	1:1,000
CD44v6 (monoclonal mouse)	Invitrogen #33-6700	1:1,000
CD74 (monoclonal mouse)	Invitrogen #14-0747-82	1:1,000
CDC25C (monoclonal rabbit)	CST#4688	1:1,000
CDK2 (monoclonal rabbit)	CST#2546	1:1,000
CDK4 (polyclonal rabbit)	Santa Cruz Biotechnology #sc-260	1:500
CDK6 (polyclonal rabbit)	Santa Cruz Biotechnology #sc-177	1:500
Claudin-1 (polyclonal rabbit)	Invitrogen #51-9000	1:1,000
CSF-1R (polyclonal rabbit)	CST#3152	1:1,000
Cyclin A2 (monoclonal mouse)	CST#4656	1:1,000
Cyclin D2 (monoclonal rabbit)	Santa Cruz Biotechnology #sc-452	1:1,000
Cyclin D3 (monoclonal rabbit)	Santa Cruz Biotechnology #sc-182	1:1,000
Cyclin E1 (monoclonal rabbit)	CST#20808	1:1,000
E-cadherin (monoclonal mouse)	CST#14472	1:500
EFGR (monoclonal rabbit)	CST#8339	1:1,000
ErbB2 (monoclonal rabbit)	CST#4290	1:1,000
ErbB3 (monoclonal rabbit)	CST#12708	1:1,000
MAP2 (monoclonal rabbit)	CST#8707	1:1,000
MIF (monoclonal rabbit)	CST #87501	1:500-1:1,000
MIF2 (DDT) (polyclonal rabbit)	CUSABIO #CSB-PA221047-50UG	1:1,000
N-cadherin (monoclonal rabbit)	CST#13116	1:1,000

Nestin (polyclonal rabbit)	MilliporeSigma #ABD69	1:10,000
p16 (polyclonal rabbit)	Proteintech #10883-1-AP	1:1000
p21(monoclonal mouse)	BD Biosciences #556430	1:500
p27 (monoclonal mouse)	BD Biosciences #610241	1:1,000
p44/42 MAPK (Erk1/2) (monoclonal mouse)	CST#4696	1:1,000
p53 (polyclonal rabbit)	Active Motif #61657	1:500
p62 (polyclonal mouse)	Proteintech #66184-1-Ig	1:1,000
Pan-Cadherin (polyclonal rabbit)	CST#4068	1:1,000
PDGFA (monoclonal mouse)	Abcam #ab51868	1:1,000
PDGFB (polyclonal rabbit)	Abcam #ab23914	1:1,000
PDGFC (polyclonal goat)	R&D Systems#AF1560	1:1,000
Phospho-EGFR Tyr1018 (monoclonal rabbit)	CST#4547	1:1,000
Phospho-ErbB3 Tyr1289 (monoclonal rabbit)	CST#2842	1:1,000
Phospho-p44/42 MAPK (Erk1/2) Thr202/Tyr204 (monoclonal rabbit)	CST#9101	1:1,000
Phospho-PDGFRB Tyr1009 (monoclonal rabbit)	CST#3124	1:1,500-1:1,000
Phospho-PDGFRB Tyr1021 (polyclonal rabbit)	R&D Systems #AF2316	1:400
Phospho-PDGFRB Tyr751 (monoclonal rabbit)	CST#4549	1:500-1:1,000
Phospho-PI3 Kinase p85 Tyr458 (monoclonal rabbit)	CST#4228	1:1,000
Phospho-Rb Ser807/811 (monoclonal rabbit)	CST#8516	1:1,000
PI3 Kinase p85 (monoclonal rabbit)	CST#4292	1:1,000

SOX2 (monoclonal rabbit)	CST#3579	1:1,000
Total PDGFRA (monoclonal rabbit)	CST#5241	1:1,000
Total PDGFRB (monoclonal rabbit)	CST#3169	1:500-1:1,000
Total Rb (monoclonal mouse)	CST#9309	1:1,000
TWIST1 (monoclonal rabbit)	CST#69366	1:500-1:1,000
Vimentin (monoclonal rabbit)	CST#5741	1:1,000
**Secondary antibodies**		
Horseradish peroxidase (HRP)-conjugated donkey anti-goat IgG	Invitrogen#A15999	1:10,000
HRP-conjugated goat anti-rabbit IgG	CST#7074	1:3,000-1:5,000
HRP-conjugated horse anti-mouse IgG	CST#7076	1:3,000-1:5,000

Supplementary Table 4 qRT-PCR primers list

**Gene**	**Forward primer (5' to 3')**	**Reverse primer (5' to 3')**
ACTB	ACCTTCTACAATGAGCTGCGT	ATAGCACAGCCTGGATAGCAA
CBL	CGC CAT GTT CCT TCC ACT AA	GAG AAG CTG CCT GGT CTA TTA C
CBLB	AGA GAC CCA GTA GAG GAA GAT G	CAA GAC CGA ACA GGA GGT TT
*CD44**	AAG ACA TCT ACC CCA GCA	GGT AGC AGG GAT TCT GTC
*CD44v6**	TCC AGG CAA CTC CTA GTA GTA C	CAG CTG TCC CTG TTG TCG AAT G

CD74	CAG ACT TGA CTG TTC CCA TAC A	AGA GCC GTA AGT CCC ATA GA
CDH1	GTC ATT GAG CCT GGC AAT TTA G	GTT GAG ACT CCT CCA TTC CTT C
CDH2	GGA TGA AAC GCC GGG ATA AA	TCT TCT TCT CCT CCA CCT TCT T
CLDN1	CCA GTT AGA AGA GGT AGT GTG AAT	CAG CCA GCT GAG CAA ATA AAG
GAPDH	TTCTCCATGGTGGTGAAGACG	ATTCCATGGCACCGTCAAGG
MAP2	GAT AGA AGG GCA ACA GAG CTA AA	GCC AGA CTC AAC ACC CAT AAA
MIF	CGGACAGGGTCTACATCAACTA	TCTTAGGCGAAGGTGGAGTT
NES	CCA TAG AGG GCA AAG TGG TAA G	CCA GAG ACT TCA GGG TTT CTT T
PDGFA	GGACAGTGCGACGGTATTT	GGAGAAACACAAAGCCAGAAAC
PDGFB	TGTAGATGGTGACCTGGGTATC	GGGTGGAGGTAGAGAGATGAAA
PDGFC	GCCACTGGACCTGCTTAAT	CTAAGTCCAACTGCCATCTCTC
PDGFD	CCCTGCTGTCCTACTGTTTAAT	CACAAAGGAGGCAGAGAGAAA
PDGFRA	CAGGTATTT CTG GGA GGT TCT G	CAT GGC AAG ACT CCA TCT CTA C
PDGFRB	GCTCACCATCATCTCCCTTATC	CTCACAGACTCAATCACCTTCC
PRKCA	CCA TCC GCT CCA CAC TAA AT	GAT CCC AGT CCC AGA TTT CTA C
SNAI1	CCA CGA GGT GTG ACT AAC TAT G	ACC AAA CAG GAG GCT GAA ATA
SOX2	GCC CTG CAG TAC AAC TCC AT	GAC TTG ACC ACC GAA CCC AT
TUBB3	CGA AGC CAG CAG TGT CTA AA	GGA GGA CGA GGC CAT AAA TAC
TWIST1	AGG CAT CAC TAT GGA CTT TCT C	GGC CAG TTT GAT CCC AGT AT

VIM	CAG CTT TCA AGT GCC TTT CTG	CTT GTA GGA GTG TCG GTT GTT
ZEB1	CTT CTC ACA CTC TGG GTC TTA TTC	CGT TCT TCC GCT TCT CTC TTA C

**CD44* and *CD44v6* primers were adapted from Yamao *et al.* (1998)(2).

Supplementary Table 5 CRISPR gRNAs list

Gene	Sense oligonucleotide (5' to 3')	Complementary oligonucleotide (5' to 3')	On-target score	Off-target score
***CD44* for CD44v6 KO**	gRNA1: TGATATTCTTCTCACAGTCC<br>gRNA2: AGGAATTGTCACGAGATGTT	gRNA1: GG ACT GTG AGA AGA ATA TCA<br>gRNA2: AA CAT CTC GTG ACA ATT CCT	gRNA 1: 56<br>gRNA 2: 54	gRNA 1: 0<br>gRNA 2: 64
CD44	gRNA1: GAATACACCTGCAAAGCGGC<br>gRNA2: AAGGGCACGTGGTGATTCCC	gRNA1: GCCGCTTTGCAGGTGTATTC<br>gRNA2: GGGAATCACCACGTGCCCTT	gRNA 1: 65<br>gRNA 2: 66	gRNA 1: 78<br>gRNA 2: 70
MIF	TTGGTGTTTACGATGAACAT	ATGTTCATCGTAAACACCAA	69	62
**Non-targeting gRNA (NT gRNA)**	GTATTACTGATATTGGTGGG	CCCACCAATATCAGTAATAC	41	0
PDGFB	CATCAAAGGAGCGGATCGAG	CTCGATCCGCTCCTTTGATG	68	88
PDGFC	CTGGTTCAAGATATCGAATA	TATTCGATATCTTGAACCAG	61	76
PDGFRB	GTCCCCTATGATCACCAACG	CGTTGGTGATCATAGGGGAC	73	87

On-target and off-target scores obtained from IDT CRISPR-Cas9 guide RNA design checker (https://www.idtdna.com/site/order/designtool/index/CRISPR_SEQUENCE)
On-target score: indicates the predicted editing performance of the gRNA at the intended target site. A higher value is better.
Off-target score: a higher value indicates lower off-target risk. Score is from 0-100.

**CD44* isoform-specific KO gRNAs were adapted from Lobo, S. *et al.* (2020)(3)

Supplementary Table 6 TIDE analysis primers list

Gene	Forward primer (5' to 3')	Reverse primer (5' to 3')

CD44 for CD44v6*	ATC AGT GGC CTG TTT CCT TG	TTT GGC TCT GTG TGA ACT GC
*CD44**	TCC CCA CTT AAG CTG AGC TCC A	TGC AAT GCT CAG GAG GCA GTG T
MIF	ACA TTG GCC GCG TTC ATG TCG T	GGG TCT CCT GGT CCT TCT GCC A
PDGFB	TGA CCA GGG CTC CAG GGA ACA G	CTG CCT GTC CGT CTC CCT GTG A
PDGFC	GTA TTG AAT ATA TCA CTG ATG A	TAT TGA CCA TTC AAG CTT TTT A
PDGFRB	CCA CCG TGG GCT TCC TCC CTA A	GAA GCT TGT GCC CTC ACC GAC C

**CD44* isoform-specific KO primers were adapted from Lobo, S. *et al.* (2020)(3)

Supplementary Table 7 RFU values of BT12 KO cells invasion/migration assay measured at Ex/Em 494/517nm

**1st Replicate**			
	Matrigel	No Matrigel	Serum-free medium
BT12 NT gRNA	66.823	186.373	35.948
	65.301	218.89	32.28
BT12 PDGFB KO	121.801	115.308	74.793
	124.364	137.522	78.993
BT12 PDGFRB KO	48.855	29.23	72.014
	50.137	49.214	96.31
BT12 MIF KO	52.936	58.453	55.95
	53.536	117.278	54.885
DAOY	495.737	1497.668	50.747
	499.408	1519.125	53.743
NIH/3T3	64.483	1093.508	48.676
	69.282	1048.102	55.727
EMPTY	9.652	15.407	6.008
	13.184	18.875	6.02
**2nd Replicate**			
	Matrigel	No Matrigel	Serum-free medium
BT12 NT gRNA	133.99	49.911	33.143
	128.068	51.583	33.083
	106.391	54.574	31.088

BT12 PDGFB KO	94.358	35.267	58.324
	102.506	35.308	60.122
	92.423	35.455	59.855
BT12 PDGFRB KO	45.012	55.072	29.331
	54.152	58.627	32.667
	57.455	59.029	29.719
BT12 MIF KO	22.441	66.666	31.032
	19.963	60.153	32.789
	14.671	61.692	33.496
**3rd Replicate**			
	Matrigel	No Matrigel	Serum-free medium
BT12 NT gRNA	31.503	63.147	20.14
	23.333	58.382	30.11
BT12 PDGFB KO	21.408	93.882	23.858
	25.81	44.686	26.513
BT12 PDGFRB KO	39.09	44.282	34.078
	17.699	26.362	34.678
BT12 MIF KO	29.828	151.399	19.277
	19.784	180.342	16.645
NIH/3T3	7.757	469.968	11.46
	17.984	787.588	67.558

RFU values of BT16 KO cells invasion/migration assay measured at Ex/Em 494/517nm

**1st Replicate**			
	Matrigel	No Matrigel	Serum-free media
BT16 NT gRNA	40.166	154.885	47.544
	82.422	243.278	36.314
	75.536	239.408	39.186
BT16 PDGFRB KO	140.888	192.979	49.654
	193.229	206.272	29.954
	190.821	146.391	28.783
BT16 MIF KO	5.983	44.438	56.58
	5.571	43.933	17.528

	5.769	43.68	23.563
**2nd Replicate**			
	Matrigel	No Matrigel	SFM
BT16 NT gRNA	45.535	162.366	54.192
	45.562	164.243	56.717
	45.562	164.243	56.717
BT16 PDGFRB KO	47.175	229.113	44.742
	51.986	237.202	45.664
	51.986	237.202	45.664
BT16 MIF KO	46.333	202.071	242.429
	44.376	180.436	216.517
	44.376	180.436	216.517
**3rd Replicate**			
	Matrigel	No Matrigel	SFM
BT16 NT gRNA	133.99	49.911	33.143
	128.068	51.583	33.083
	106.391	54.574	31.088
BT16 PDGFRB KO	45.012	55.072	29.331
	54.152	58.627	32.667
	57.455	59.029	29.719
BT16 MIF KO	22.441	66.666	31.032
	19.963	60.153	32.789
	14.671	61.692	33.496

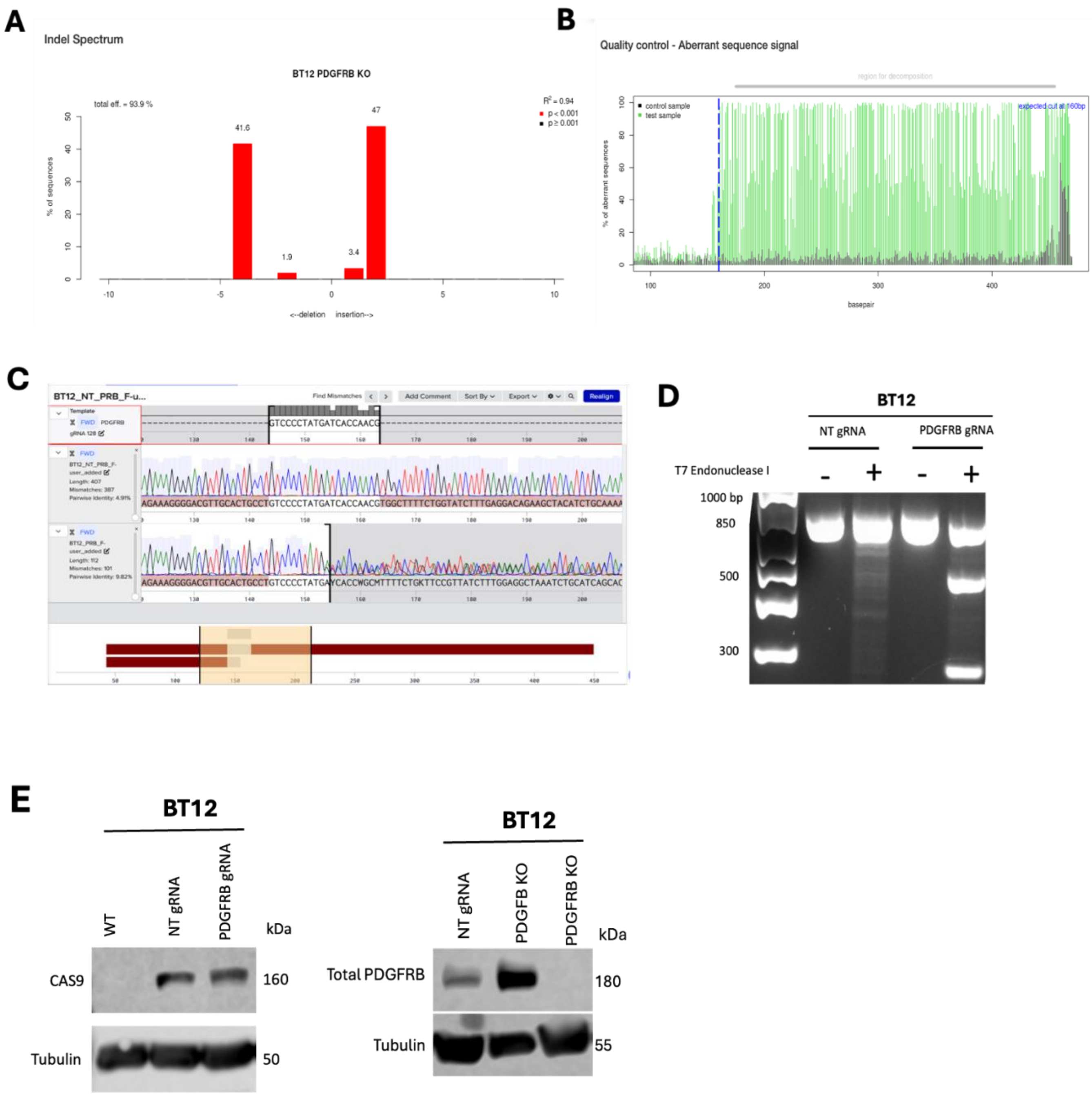

A
Indel Spectrum
BT12 PDGFRB KO
total eff. = 93.9 %
R^2 = 0.94
p < 0.001
p ≥ 0.001
% of sequences
41.6
1.9
3.4
47
<--deletion insertion-->
B
Quality control - Aberrant sequence signal
region for decomposition
control sample
test sample
expected cut at 160bp
% of aberrant sequences
basepair
C
BT12_NT_PRB_F-u...
Find Mismatches
Add Comment
Sort By
Export
Realign
Template
FWD PDGFRB gRNA 128
GTCCCCTATGATCACCAACG
AGAAAGGGGACGTTGCACTGCCTGTCCCCTATGATCACCAACGTGGCTTTTCTGGTATCTTTGAGGACAGAAGCTACATCTGCAAAA
AGAAAGGGGACGTTGCACTGCCTGTCCCCTATGAYCACCWGCMTTTTCTGKTTCCGTTATCTTTGGAGGCTAAATCTGCATCAGCAC
D
BT12
NT gRNA
PDGFRB gRNA
T7 Endonuclease I
-
+
-
+
1000 bp
850
500
300
E
BT12
WT
NT gRNA
PDGFRB gRNA
kDa
CAS9
160
Tubulin
50
BT12
NT gRNA
PDGFB KO
PDGFRB KO
kDa
Total PDGFRB
180
Tubulin
55

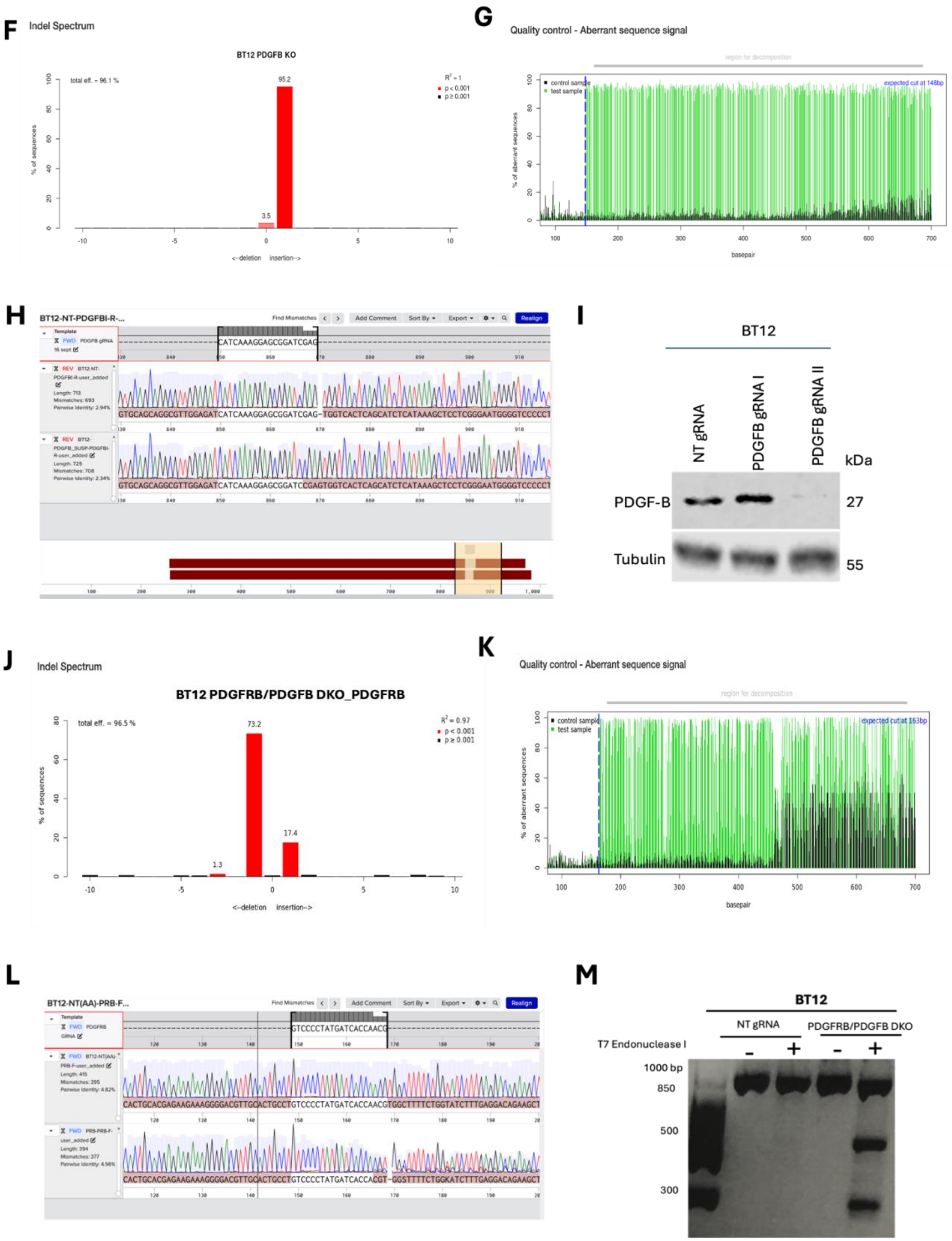
F
Indel Spectrum
BT12 PDGFB KO
total eff. = 96.1 %
95.2
3.5
% of sequences
<--deletion insertion-->
G
Quality control - Aberrant sequence signal
region for decomposition
control sample
test sample
expected cut at 148bp
% of aberrant sequences
basepair
H
CATCAAAGGAGCGGATCGAG
I
BT12
NT gRNA
PDGFB gRNA I
PDGFB gRNA II
kDa
PDGF-B
27
Tubulin
55
J
Indel Spectrum
BT12 PDGFRB/PDGFB DKO_PDGFRB
total eff. = 96.5 %
R^2 = 0.97
p < 0.001
p ≥ 0.001
73.2
17.4
1.3
% of sequences
<--deletion insertion-->
K
Quality control - Aberrant sequence signal
region for decomposition
control sample
test sample
expected cut at 163bp
% of aberrant sequences
basepair
L
GTCCCCTATGATCACCAACG
M
BT12
NT gRNA
PDGFRB/PDGFB DKO
T7 Endonuclease I
-
+
-
+
1000 bp
850
500
300

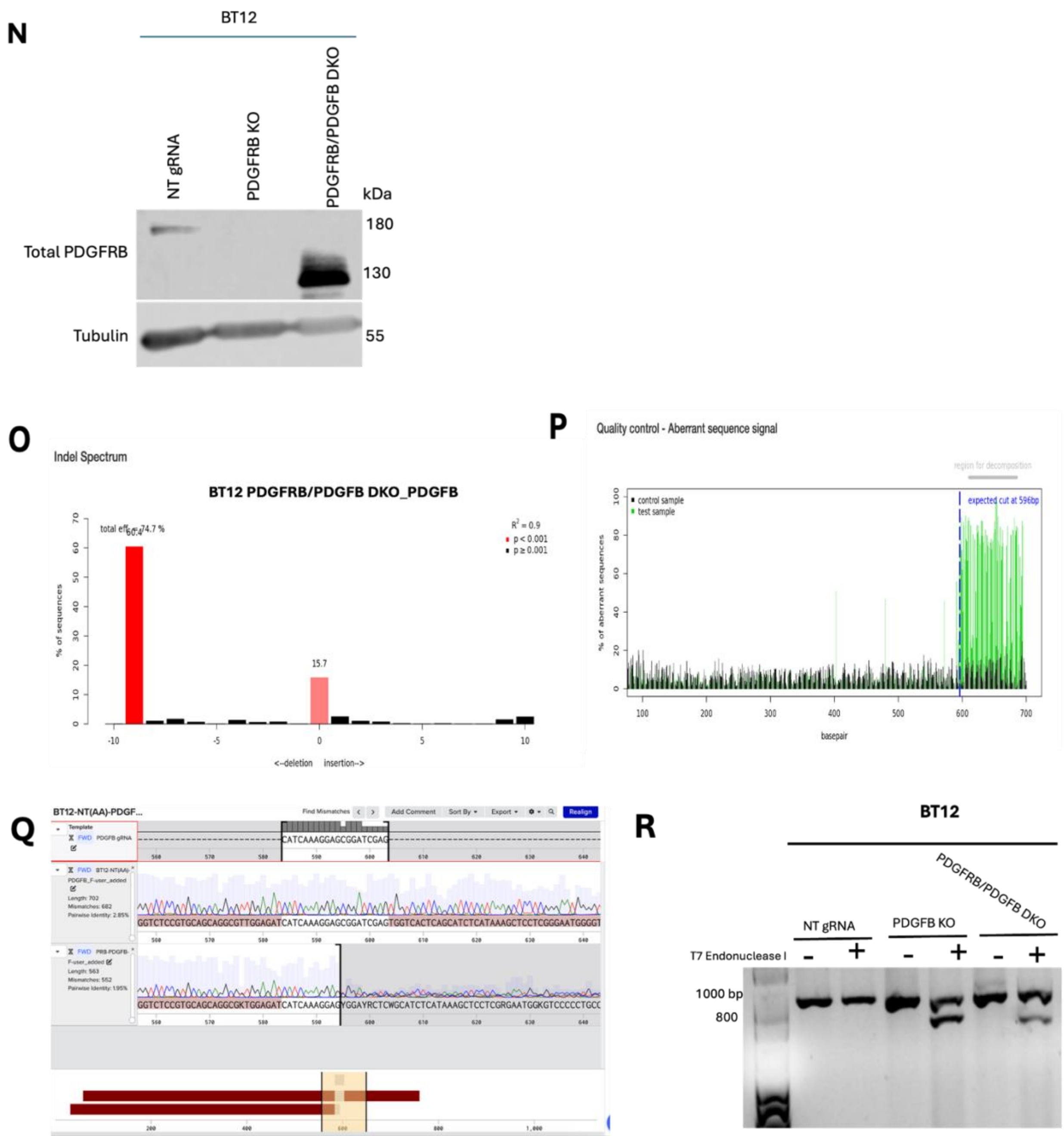

Supplementary Fig. 1. Validation of BT12 PDGFB KO, PDGFRB KO, and PDGFRB/PDGFB DKO

- **A.** Tracking of Indels by Decomposition (TIDE) analysis of BT12 PDGFRB KO relative to BT12 NT gRNA. Analysis was conducted using TIDE web tool https://tide.nki.nl/
- **B.** Corresponding TIDE analysis quality control. Analysis was conducted using TIDE web tool https://tide.nki.nl/

- **C.** Sanger sequences alignment of PDGFRB gRNA, BT12 NT gRNA, and BT12 PDGFRB KO. Alignment was conducted using Benchling web tool https://www.benchling.com/
- **D.** T7 Endonuclease I assay results of BT12 NT gRNA and BT12 PDGFRB KO. PCR products were amplified using primers flanking the PDGFRB gRNA site. +/- indicate presence and absence of T7 Endonuclease I, respectively. BT12 PDGFRB KO PCR product treated with T7 Endonuclease I displays three fragments, indicative of indels presence.
- **E.** Left: Western blot validation of the expression of Cas9 protein in BT12 NT gRNA and BT12 PDGFRB KO. BT12 wild-type (WT) cells were used as a negative control for Cas9 expression. Right: Western blot validation of the loss of PDGFRB protein expression in BT12 PDGFRB KO cells.
- **F-H.** TIDE analysis and Sanger sequences alignment of BT12 PDGFB KO relative to BT12 NT gRNA.
- **I.** Western blot validation of the loss of PDGFB protein expression in BT12 PDGFB KO cells. Two different gRNAs were optimized; gRNA I-transfected cells maintained PDGFB protein expression. gRNA II was selected for downstream experiments.
- **J-M.** Genomic validation of PDGFRB KO in BT12 PDGFRB/PDGFB DKO cells.
- **N.** Western blot analysis of PDGFRB expression in BT12 PDGFRB/PDGFB DKO. BT12 PDGFRB KO showed loss of PDGFRB protein expression. BT12 PDGFRB/PDGFB DKO expressed a smaller band detected around 130 kDa.
- **O-Q.** Genomic validation of PDGFB KO in BT12 PDGFRB/PDGFB DKO cells.
- **R.** T7 Endonuclease I assay showing cleavage of PDGFB PCR product from BT12 PDGFB KO and BT12 PDGFRB/PDGFB DKO, indicative of presence of indels.

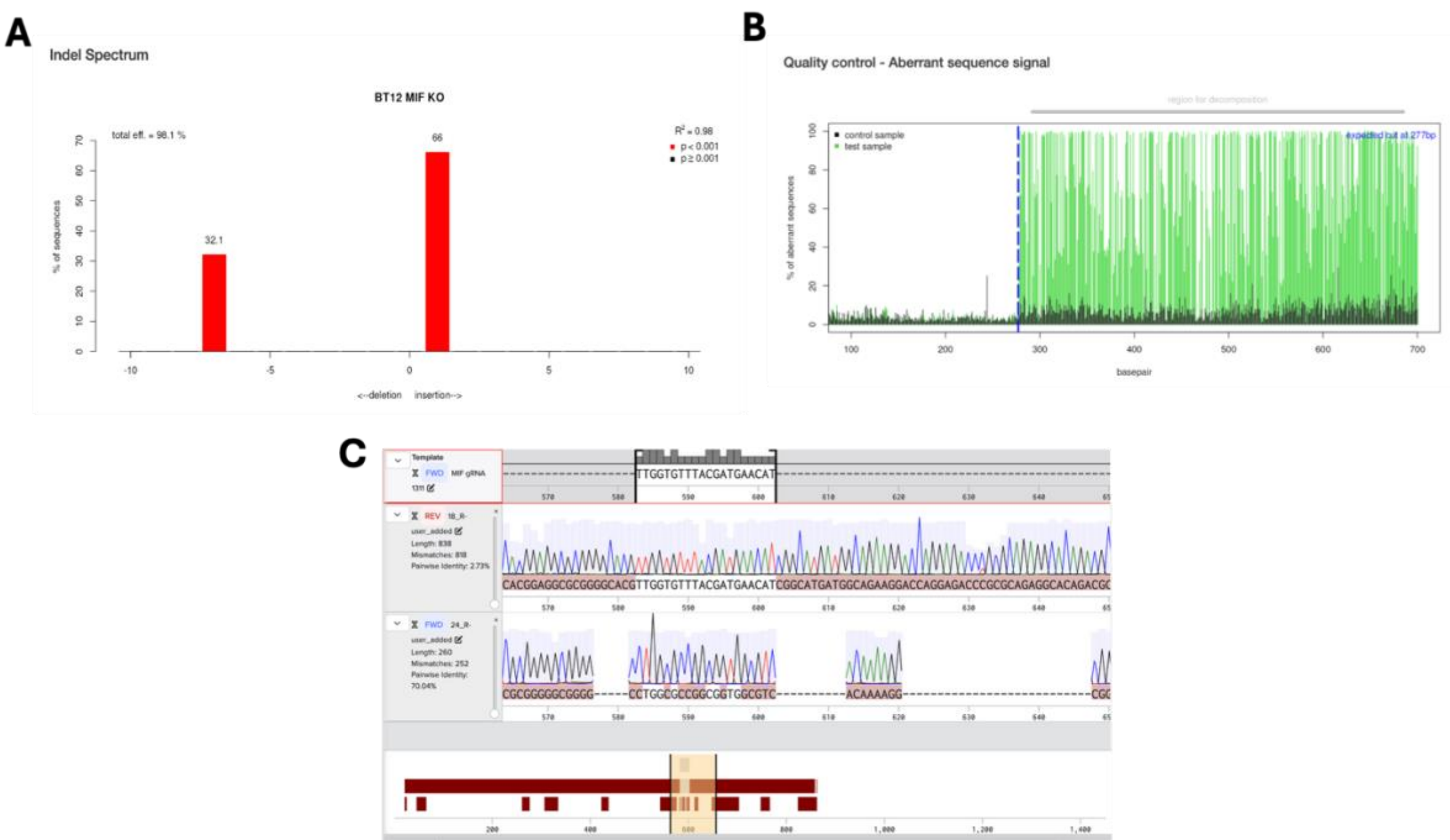

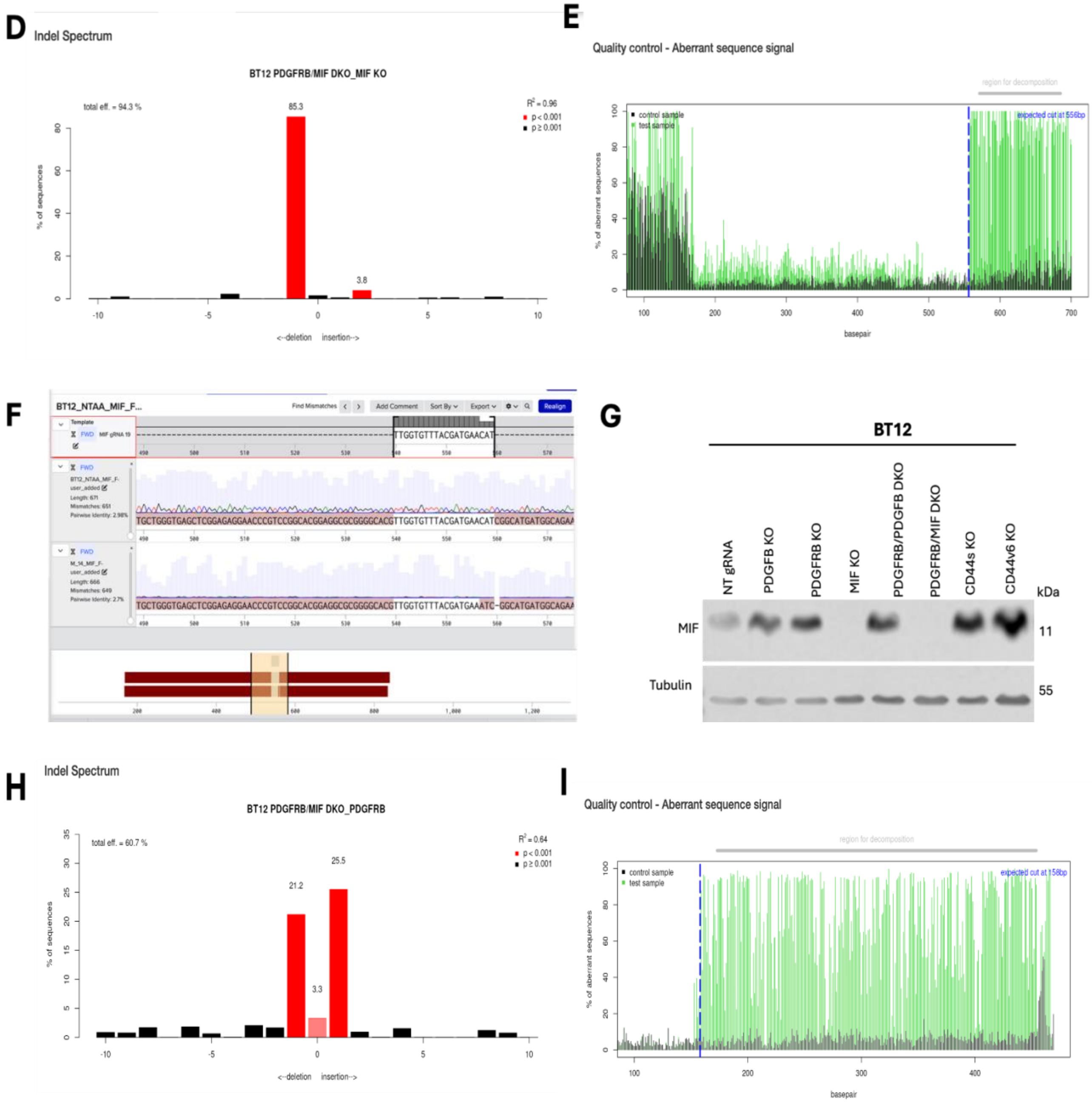
D
Indel Spectrum
BT12 PDGFRB/MIF DKO_MIF KO
total eff. = 94.3 %
85.3
3.8
% of sequences
<--deletion insertion-->
E
Quality control - Aberrant sequence signal
region for decomposition
control sample
test sample
expected cut at 556bp
% of aberrant sequences
basepair
F
BT12_NTAA_MIF_F...
Find Mismatches
Add Comment
Sort By
Export
Realign
Template
TTGGTGTTTACGATGAACAT
TGCTGGGTGAGCTCGGAGAGGAACCCGTCCGGCACGGAGGCGCGGGGCACGTTGGTGTTTACGATGAACATCGGCATGATGGCAGAA
TGCTGGGTGAGCTCGGAGAGGAACCCGTCCGGCACGGAGGCGCGGGGCACGTTGGTGTTTACGATGAAATC-GGCATGATGGCAGAA
G
BT12
NT gRNA
PDGFB KO
PDGFRB KO
MIF KO
PDGFRB/PDGFB DKO
PDGFRB/MIF DKO
CD44s KO
CD44v6 KO
kDa
MIF
11
Tubulin
55
H
Indel Spectrum
BT12 PDGFRB/MIF DKO_PDGFRB
total eff. = 60.7 %
21.2
25.5
3.3
% of sequences
<--deletion insertion-->
I
Quality control - Aberrant sequence signal
region for decomposition
control sample
test sample
expected cut at 158bp
% of aberrant sequences
basepair

J

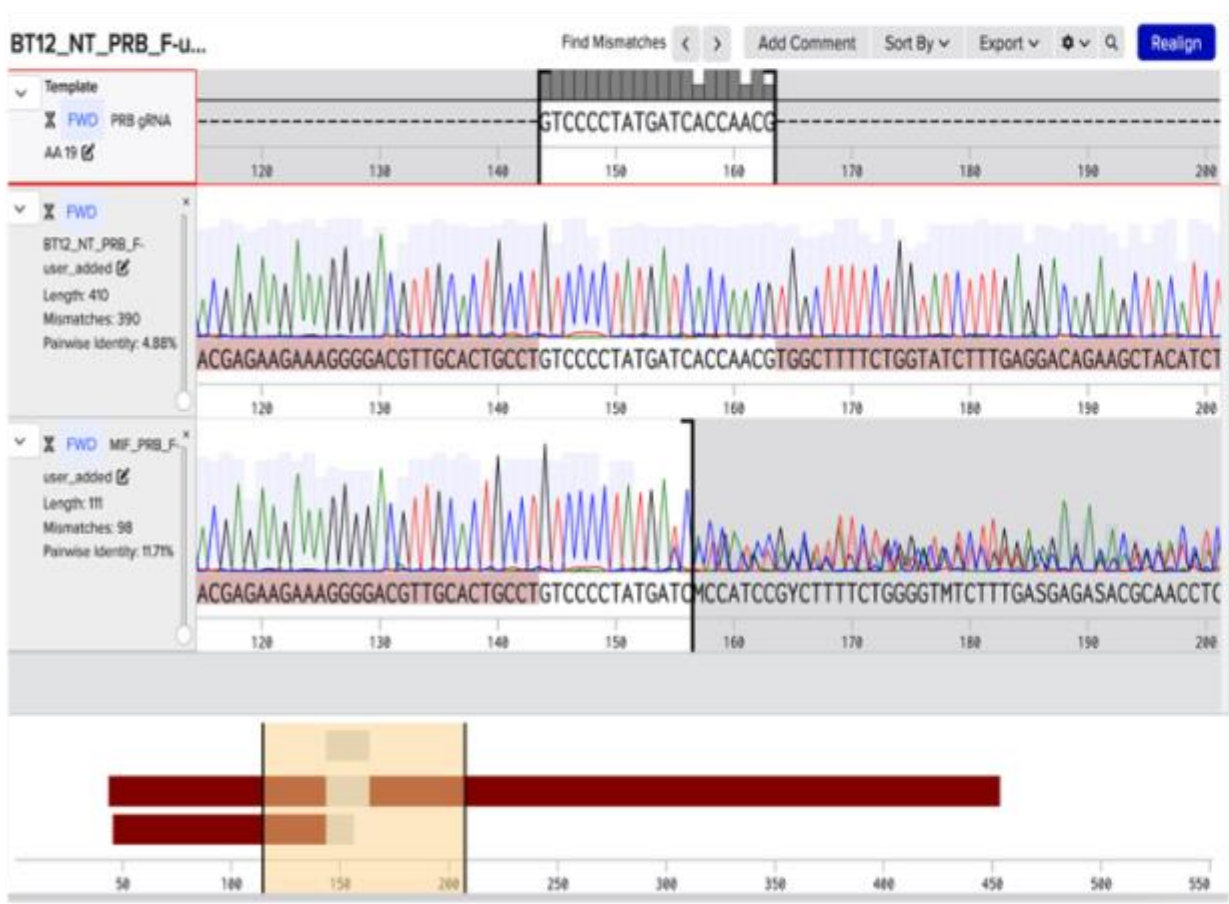
BT12_NT_PRB_F-u...
Find Mismatches
Add Comment
Sort By
Export
Realign
Template
FWD PRB gRNA
GTCCCCTATGATCACCAACG
FWD
ACGAGAAGAAAGGGGACGTTGCACTGCCTGTCCCCTATGATCACCAACGTGGCTTTTCTGGTATCTTTGAGGACAGAAGCTACATCT
FWD
ACGAGAAGAAAGGGGACGTTGCACTGCCTGTCCCCTATGATCMCCATCCGYCTTTTCTGGGGTMTCTTTGASGAGASACGCAACCTC

K

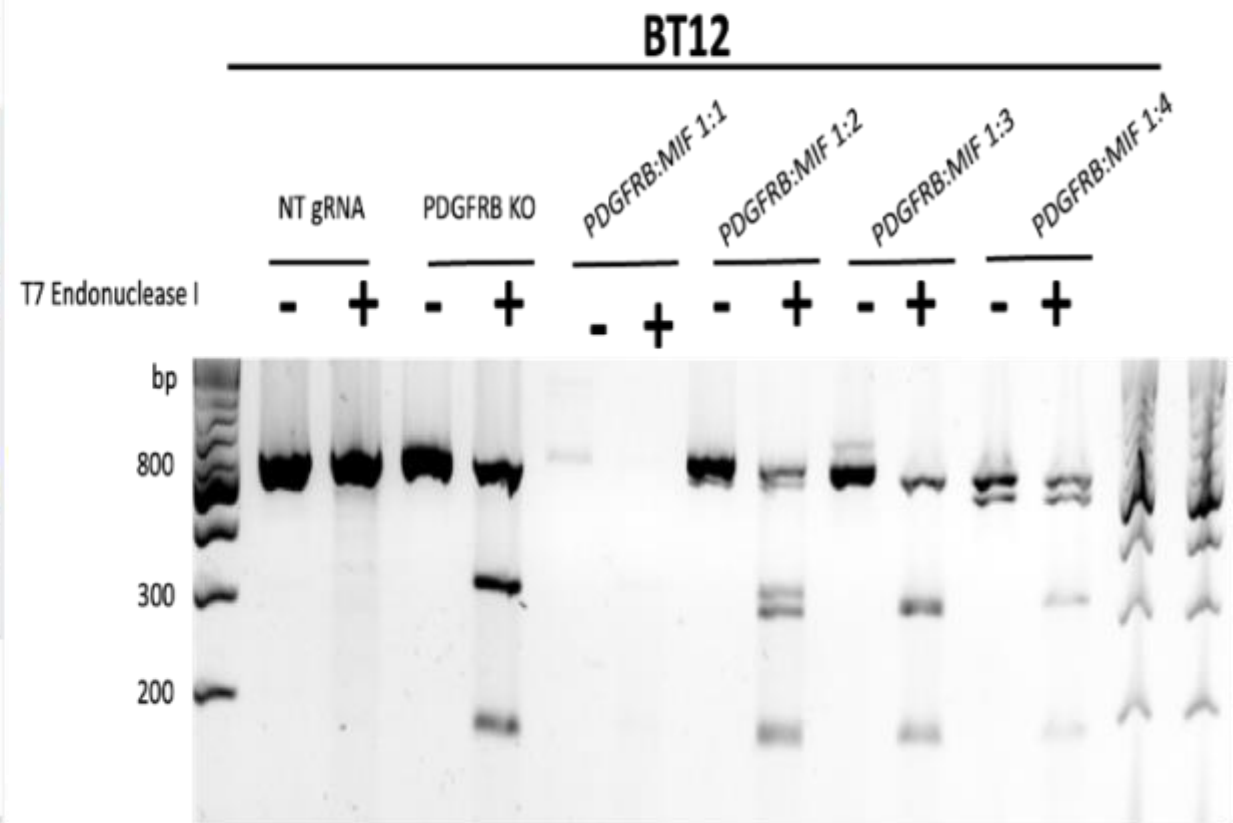
BT12
NT gRNA
PDGFRB KO
PDGFRB:MIF 1:1
PDGFRB:MIF 1:2
PDGFRB:MIF 1:3
PDGFRB:MIF 1:4
T7 Endonuclease I
- + - + - + - + - + - +
bp
800
300
200

L

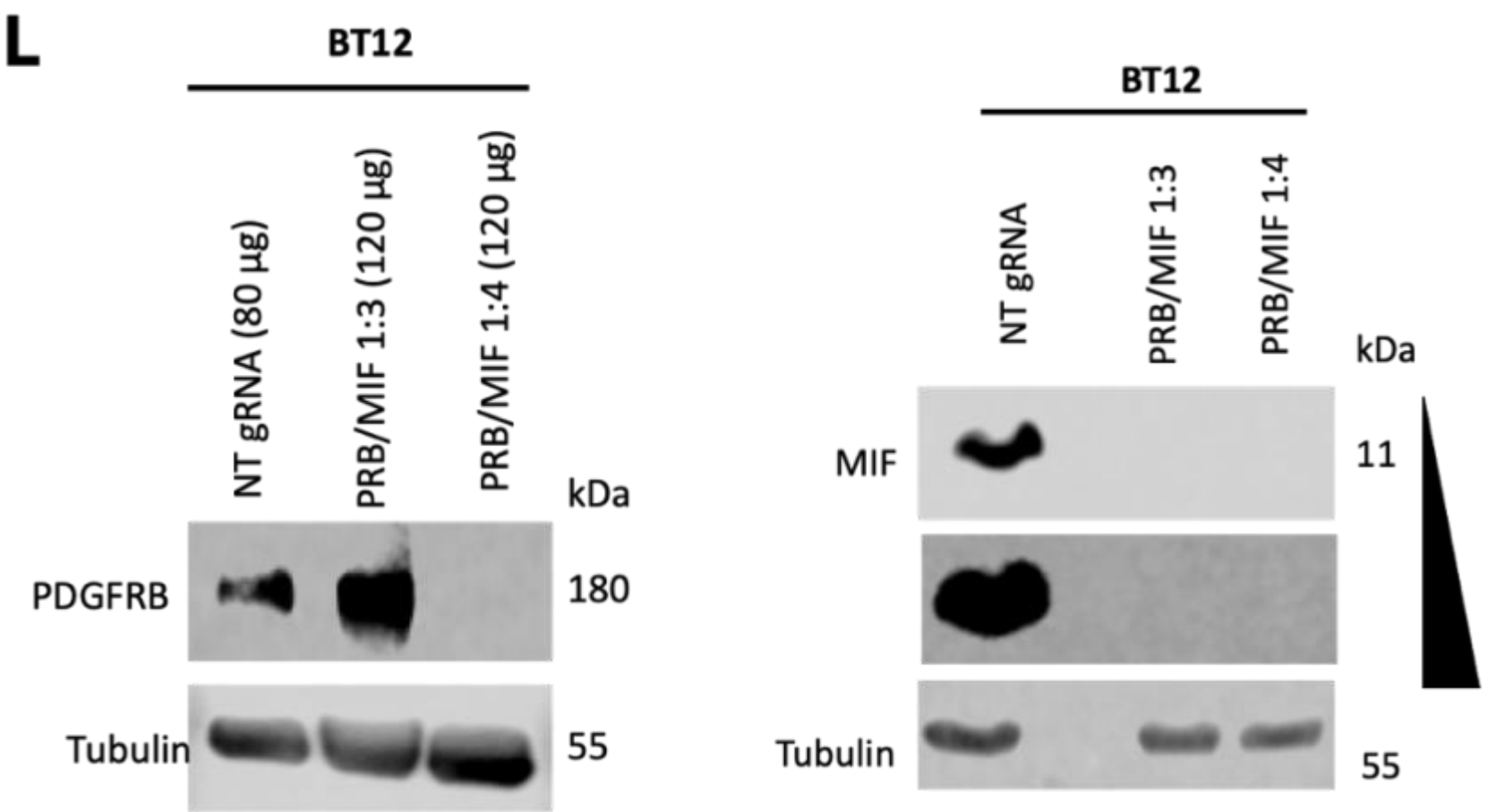
BT12
NT gRNA (80 µg)
PRB/MIF 1:3 (120 µg)
PRB/MIF 1:4 (120 µg)
kDa
PDGFRB
180
Tubulin
55
BT12
NT gRNA
PRB/MIF 1:3
PRB/MIF 1:4
kDa
MIF
11
Tubulin
55

M

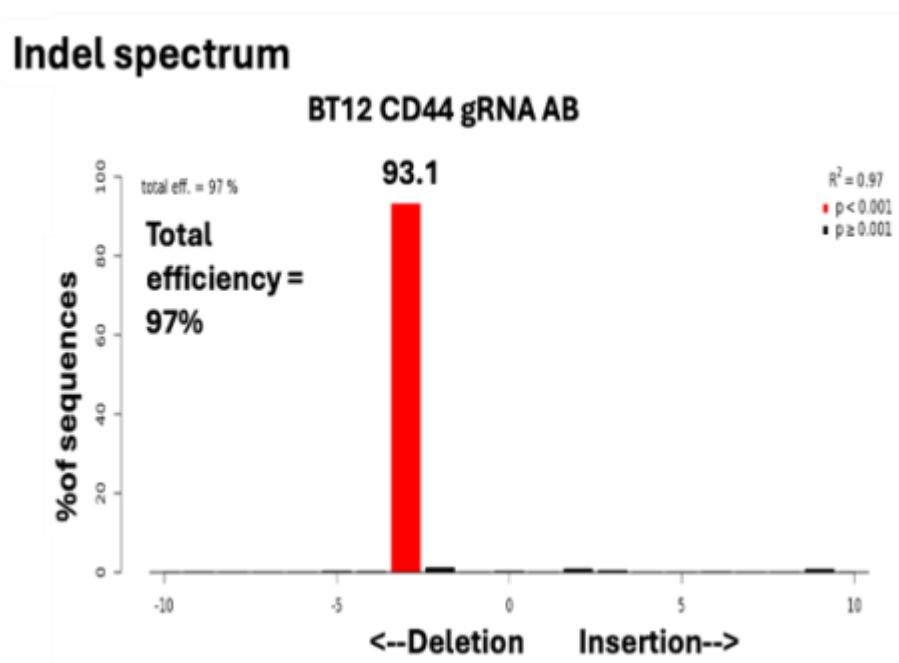
Indel spectrum
BT12 CD44 gRNA AB
93.1
Total efficiency = 97%
%of sequences
<--Deletion
Insertion-->

N

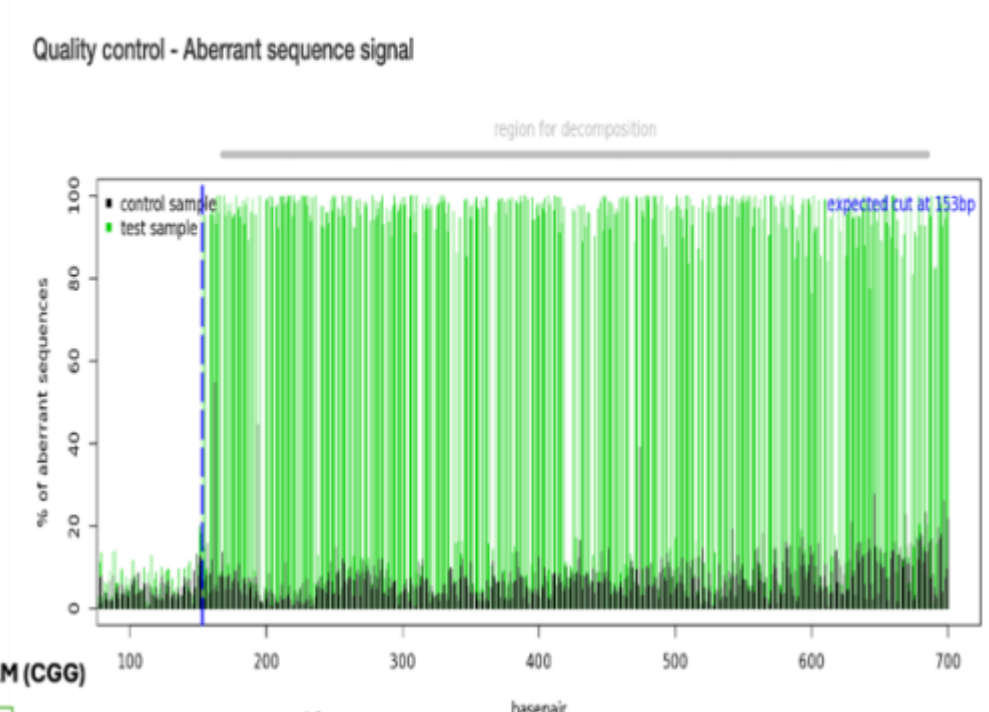
Quality control - Aberrant sequence signal
region for decomposition
control sample
test sample
expected cut at 153bp
% of aberrant sequences
basepair

O

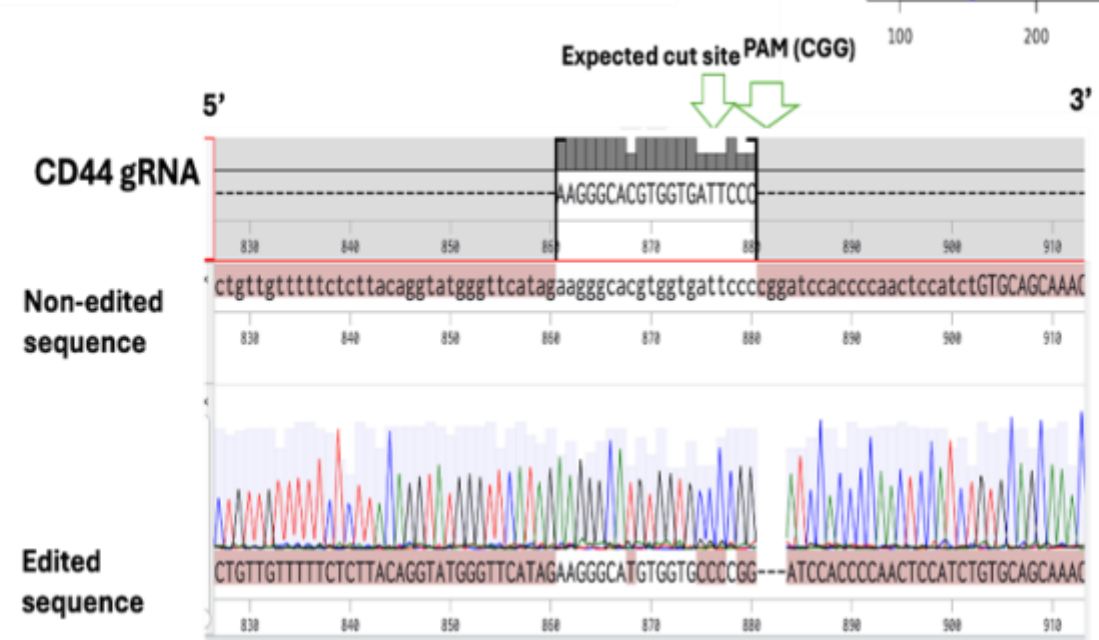
Expected cut site
PAM (CGG)
5'
3'
CD44 gRNA
AAGGGCACGTGGTGATTCCC
Non-edited sequence
Edited sequence

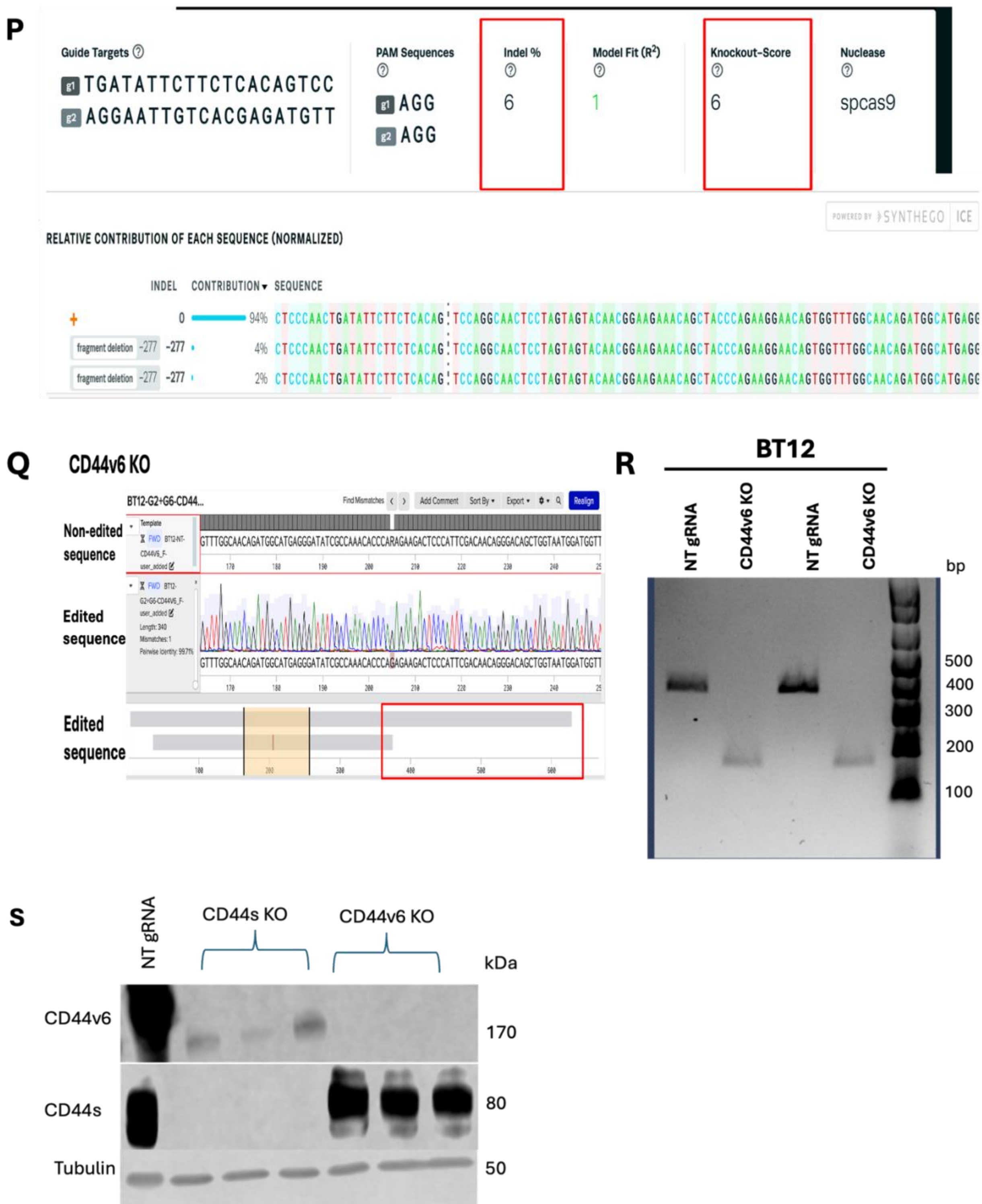

Supplementary Fig. 2. Validation of BT12 MIF KO, PDGFRB/MIF DKO, CD44s KO, and CD44v6 KO

- **A-C.** Genomic validation of BT12 MIF KO
- **D-F.** Genomic validation of MIF KO in BT12 PDGFRB/MIF DKO
- **G.** Western blot validation of loss of MIF protein in BT12 MIF KO and BT12 PDGFRB/MIF DKO

- **H-J.** Genomic validation of PDGFRB KO in BT12 PDGFRB/MIF DKO
- **K.** T7 Endonuclease I assay validation of PDGFRB KO in BT12 PDGFRB/MIF DKO. PDGFRB KO was included as a positive control; PDGFRB/MIF 1:1, 1:2, 1:3, and 1:4 represent different conditions for DKO optimization, PDGFRB gRNA:MIF gRNA ratio. PDGFRB/MIF DKO 1:4 was selected for subsequent experiments.
- **L.** Western blot validation of PDGFRB and MIF protein loss in BT12 PDGFRB/MIF DKO. BT12 PDGFRB/MIF DKO 1:4 was selected for subsequent experiments based on the dual loss of PDGFRB and MIF expression. PRB indicates PDGFRB.
- **M-O.** Genomic validation of BT12 CD44s KO.
- **P-Q.** Genomic validation of BT12 CD44v6 KO. Interference of CRISPR edits (ICE) analysis of Sanger sequences was achieved using Synthego ICE analysis tool (ice.synthego.com). The analysis shows ~277 bp fragment deletion, corresponding approximately to the region around CD44 exon 11 (or variable exon 6), as described by Lobo, *et al. (3)*.
- **R.** PCR product analysis of BT12 NT gRNA and BT12 CD44v6 KO, using primers flanking the region including intron v5, exon v6, and intron v6, as described by Lobo, *et al. (3)*. Two biological replicates were used. BT12 CD44v6 product is smaller relative to that of BT12 NT gRNA, consistent with the fragment deletion.
- **S.** Western blot validation of CD44s loss in BT12 CD44s KO, and CD44v6 loss in BT12 CD44v6 KO. Three biological replicates of CD44s KO and CD44v6 KO were used, grouped under a brace. BT12 CD44s KO shows loss of CD44s and a decrease in CD44v6. BT12 CD44v6 KO shows loss of CD44v6 and no change in CD44s.

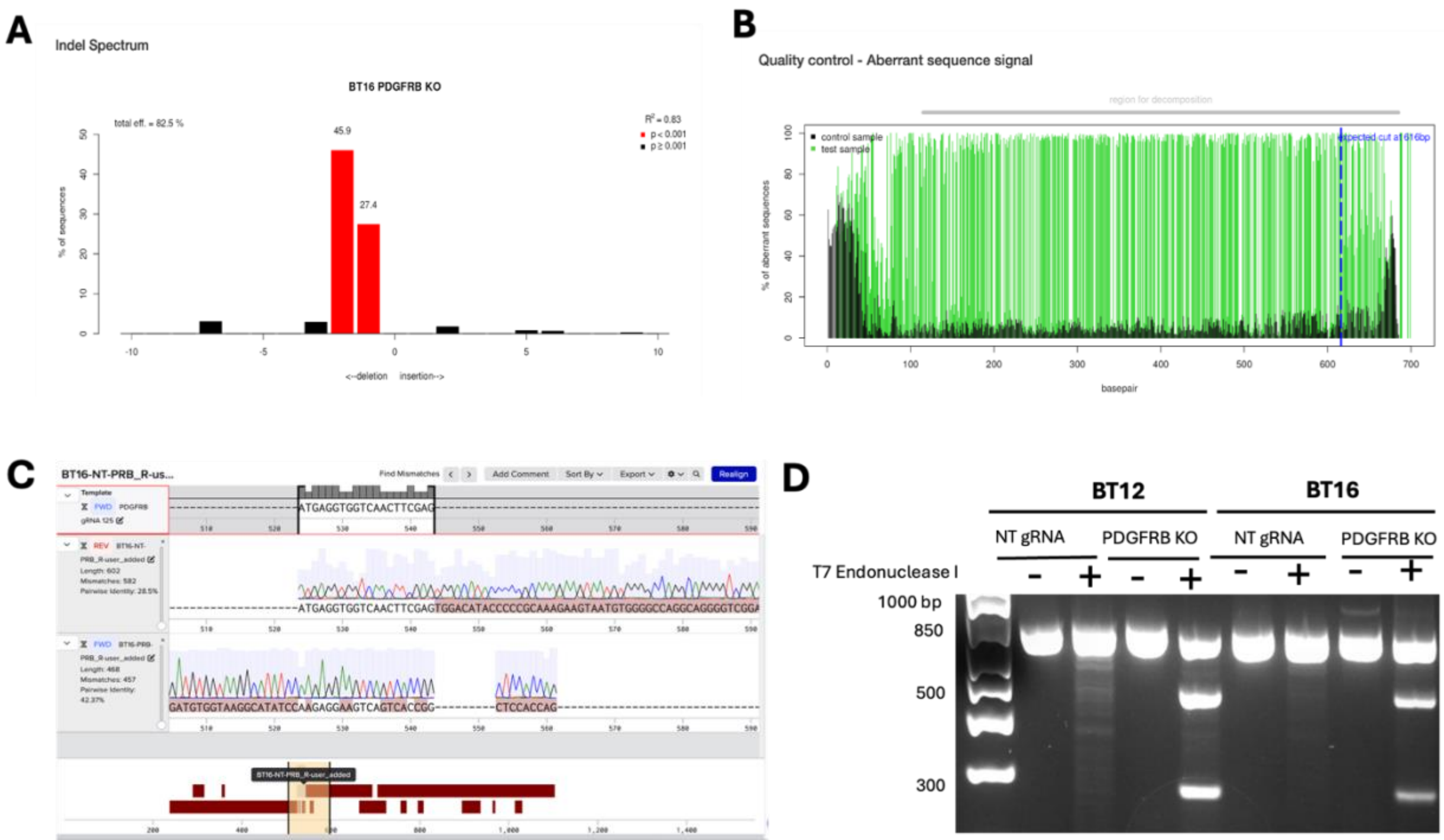

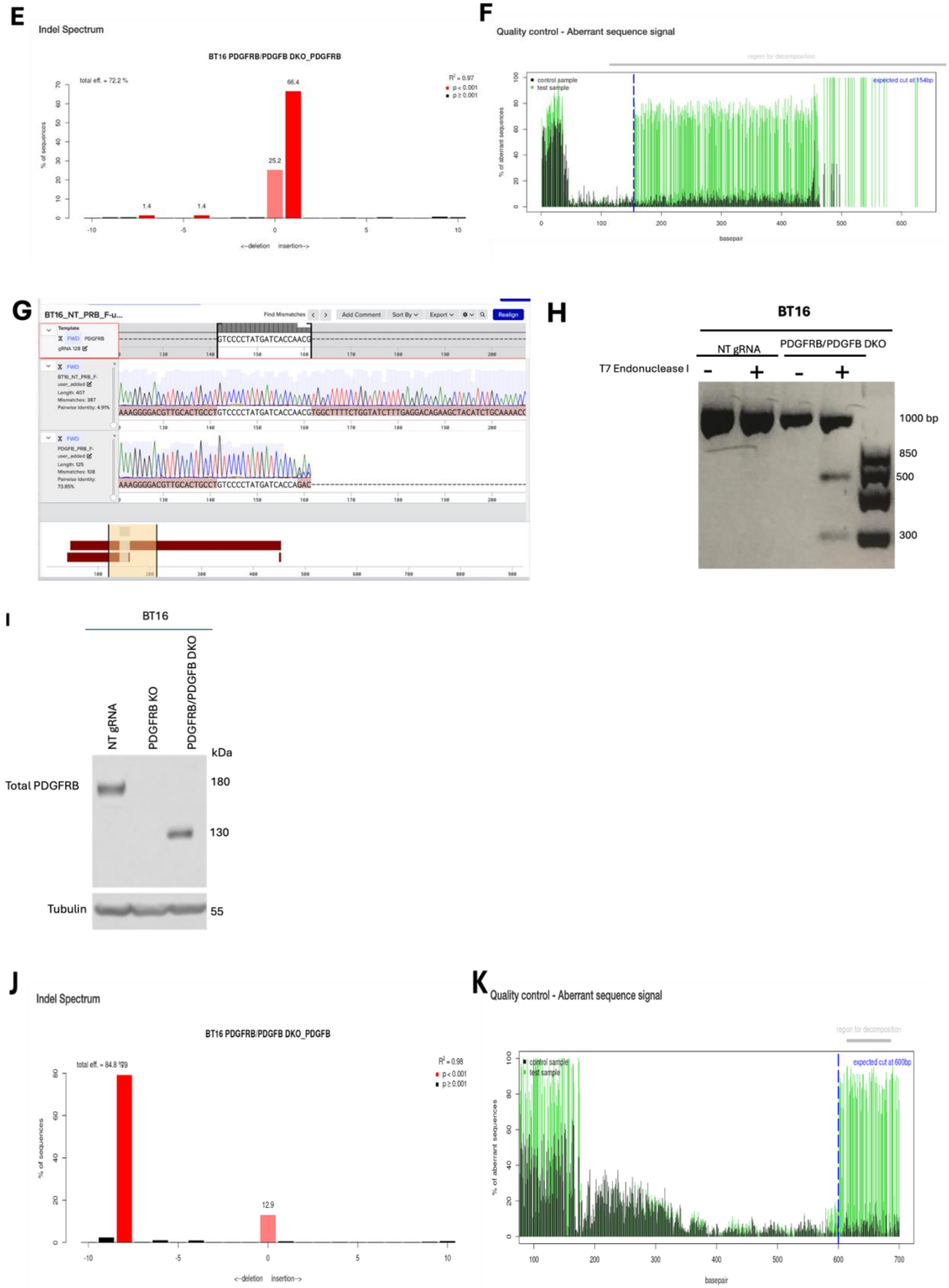
E
Indel Spectrum
BT16 PDGFRB/PDGFB DKO_PDGFRB
total eff. = 72.2 %
R² = 0.97
p < 0.001
p ≥ 0.001
% of sequences
66.4
25.2
1.4
1.4
<--deletion insertion-->
F
Quality control - Aberrant sequence signal
region for decomposition
control sample
test sample
expected cut at 154bp
% of aberrant sequences
basepair
G
BT16_NT_PRB_F-u...
Find Mismatches
Add Comment
Sort By
Export
Realign
Template
FWD PDGFRB
gRNA 128
GTCCCCTATGATCACCAACG
BT16_NT_PRB_F-
user_added
Length: 407
Mismatches: 387
Pairwise Identity: 4.91%
AAAGGGGACGTTGCACTGCCTGTCCCCTATGATCACCAACGTGGCTTTTCTGGTATCTTTGAGGACAGAAGCTACATCTGCAAAACC
PDGFB_PRB_F-
user_added
Length: 125
Mismatches: 108
Pairwise Identity: 73.85%
AAAGGGGACGTTGCACTGCCTGTCCCCTATGATCACCAGAC
H
BT16
NT gRNA
PDGFRB/PDGFB DKO
T7 Endonuclease I
-
+
-
+
1000 bp
850
500
300
I
BT16
NT gRNA
PDGFRB KO
PDGFRB/PDGFB DKO
kDa
Total PDGFRB
180
130
Tubulin
55
J
Indel Spectrum
BT16 PDGFRB/PDGFB DKO_PDGFB
total eff. = 84.8 %
79
R² = 0.98
p < 0.001
p ≥ 0.001
% of sequences
12.9
<--deletion insertion-->
K
Quality control - Aberrant sequence signal
region for decomposition
control sample
test sample
expected cut at 600bp
% of aberrant sequences
basepair

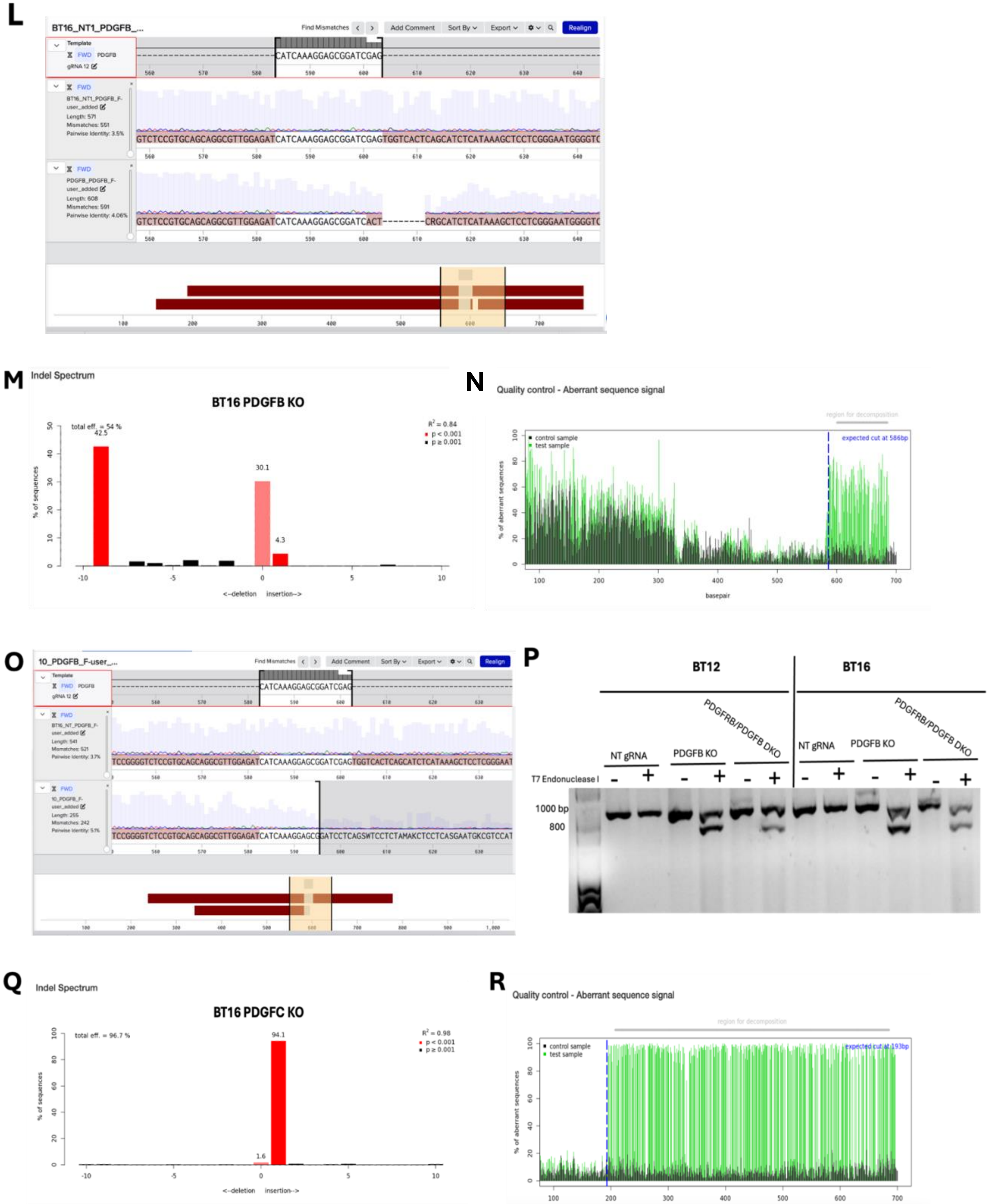
L
BT16_NT1_PDGFB_...
Template
FWD PDGFB
gRNA 12
CATCAAAGGAGCGGATCGAG
BT16_NT1_PDGFB_F-user_added
Length: 571
Mismatches: 551
Pairwise Identity: 3.5%
GTCTCCGTGCAGCAGGCGTTGGAGATCATCAAAGGAGCGGATCGAGTGGTCACTCAGCATCTCATAAAGCTCCTCGGGAATGGGGTC
PDGFB_PDGFB_F-user_added
Length: 608
Mismatches: 591
Pairwise Identity: 4.06%
GTCTCCGTGCAGCAGGCGTTGGAGATCATCAAAGGAGCGGATCACT--------CRGCATCTCATAAAGCTCCTCGGGAATGGGGTC
M
Indel Spectrum
BT16 PDGFB KO
total eff. = 54 %
R^2 = 0.84
p < 0.001
p ≥ 0.001
42.5
30.1
4.3
% of sequences
<--deletion insertion-->
N
Quality control - Aberrant sequence signal
region for decomposition
control sample
test sample
expected cut at 586bp
% of aberrant sequences
basepair
O
10_PDGFB_F-user_...
Template
FWD PDGFB
gRNA 12
CATCAAAGGAGCGGATCGAG
BT16_NT_PDGFB_F-user_added
Length: 541
Mismatches: 521
Pairwise Identity: 3.7%
TCCGGGGTCTCCGTGCAGCAGGCGTTGGAGATCATCAAAGGAGCGGATCGAGTGGTCACTCAGCATCTCATAAAGCTCCTCGGGAAT
10_PDGFB_F-user_added
Length: 255
Mismatches: 242
Pairwise Identity: 5.1%
TCCGGGGTCTCCGTGCAGCAGGCGTTGGAGATCATCAAAGGAGCGGATCCTCAGSWTCCTCTAMAKCTCCTCASGAATGKCGTCCAT
P
BT12
BT16
NT gRNA
PDGFB KO
PDGFRB/PDGFB DKO
T7 Endonuclease I
- + - + - + - + - + - +
1000 bp
800
Q
Indel Spectrum
BT16 PDGFC KO
total eff. = 96.7 %
R^2 = 0.98
p < 0.001
p ≥ 0.001
94.1
1.6
% of sequences
<--deletion insertion-->
R
Quality control - Aberrant sequence signal
region for decomposition
control sample
test sample
expected cut at 193bp
% of aberrant sequences

S

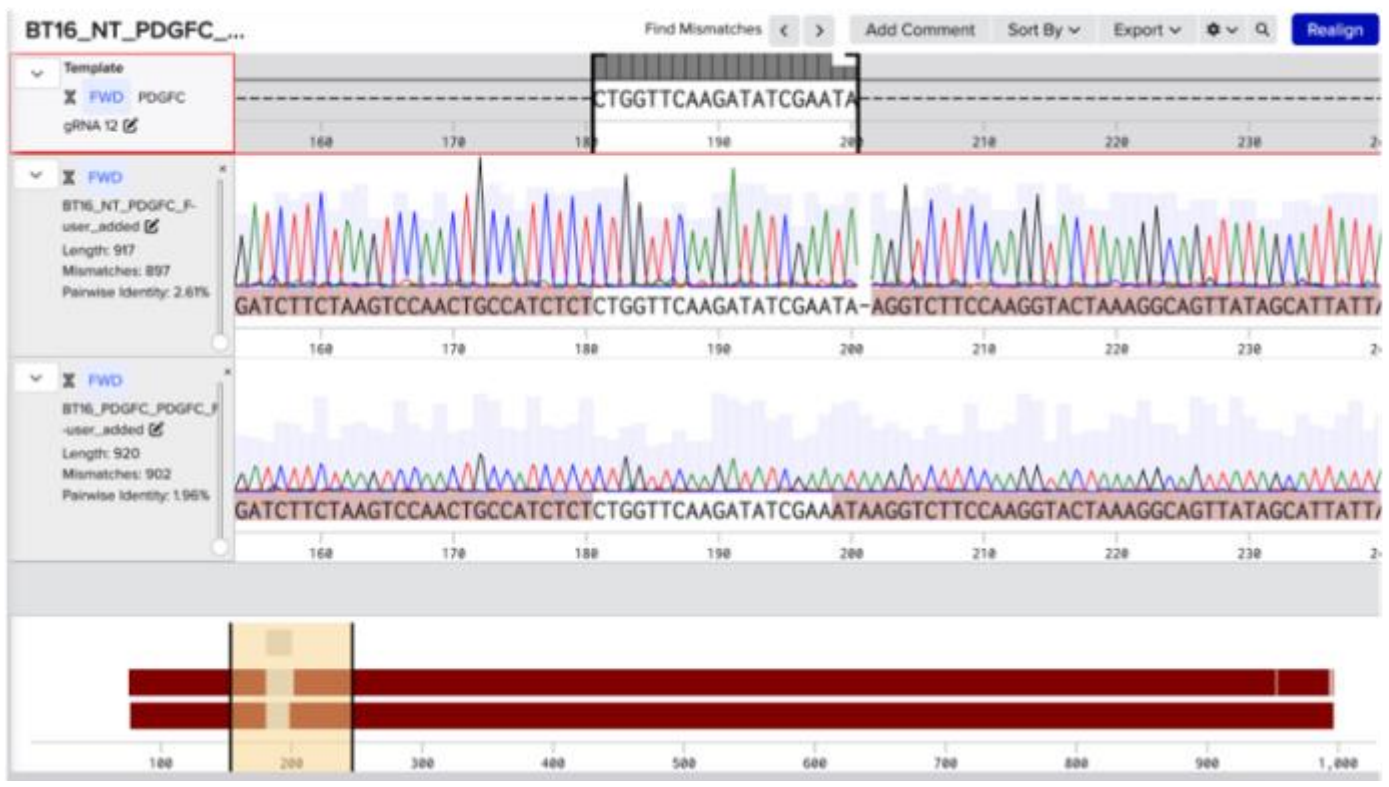

T

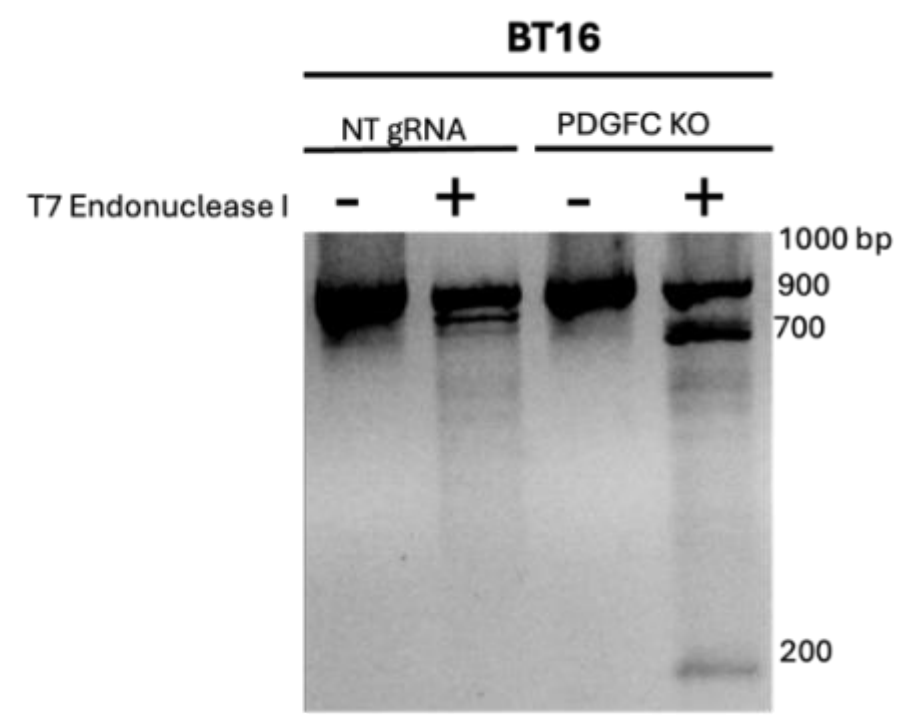

U

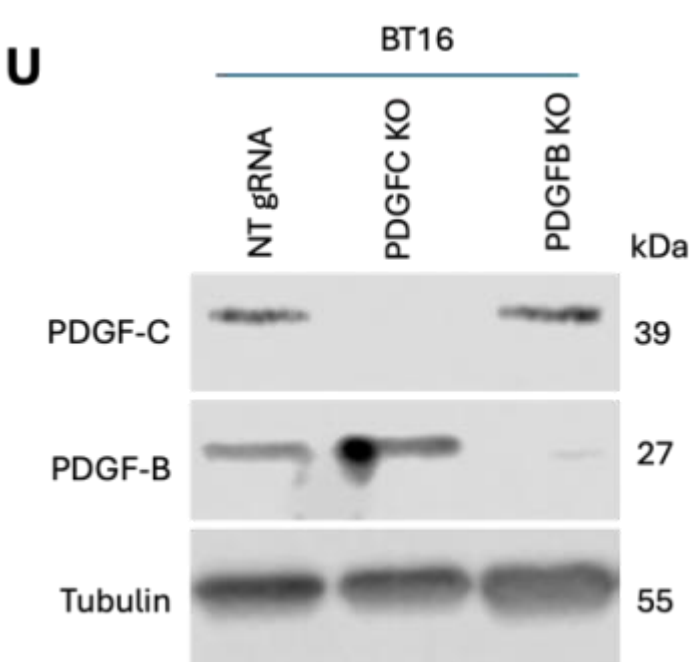

Supplementary Fig. 3. Validation of BT16 PDGFB KO, PDGFC KO, PDGFRB KO, and PDGFRB/PDGFB DKO

- **A.** Tracking of Indels by Decomposition (TIDE) analysis of BT16 PDGFRB KO relative to BT16 NT gRNA. Analysis was conducted using TIDE web tool https://tide.nki.nl/
- **B.** Corresponding TIDE analysis quality control. Analysis was conducted using TIDE web tool https://tide.nki.nl/
- **C.** Sanger sequences alignment of PDGFRB gRNA, BT16 NT gRNA, and BT16 PDGFRB KO. Alignment was conducted using Benchling web tool https://www.benchling.com/
- **D.** T7 Endonuclease I assay results of BT16 NT gRNA and BT16 PDGFRB KO. PCR products were amplified using primers flanking the PDGFRB gRNA site. +/- indicate presence and absence of T7 Endonuclease I, respectively. BT16 PDGFRB KO PCR product treated with T7 Endonuclease I displays three fragments, indicative of indels presence.
- **E-H.** Genomic validation of PDGFRB KO in BT16 PDGFRB/PDGFB DKO.
- **I.** Western blot analysis of PDGFRB expression in BT16 PDGFRB KO and BT16 PDGFRB/PDGFB DKO. BT16 PDGFRB KO showed loss of PDGFRB protein expression. BT16 PDGFRB/PDGFB DKO showed a smaller band detected around 130 kDa, similar to that observed in BT12 PDGFRB/PDGFB DKO.
- **J-L.** Genomic validation of PDGFB KO in BT16 PDGFRB/PDGFB DKO.
- **M-O.** genomic validation of PDGFB KO in BT16 PDGFB KO.
- **P.** T7 Endonuclease I assay showing cleavage of PDGFB PCR product from BT16 PDGFB KO and BT16 PDGFRB/PDGFB DKO, indicative of presence of indels.
- **Q-T.** Genomic validation of PDGFC KO in BT16 PDGFC KO.
- **U.** Western blot analysis showing loss of PDGFB and PDGFC protein expression in BT16 PDGFB KO and BT16 PDGFC KO, respectively.

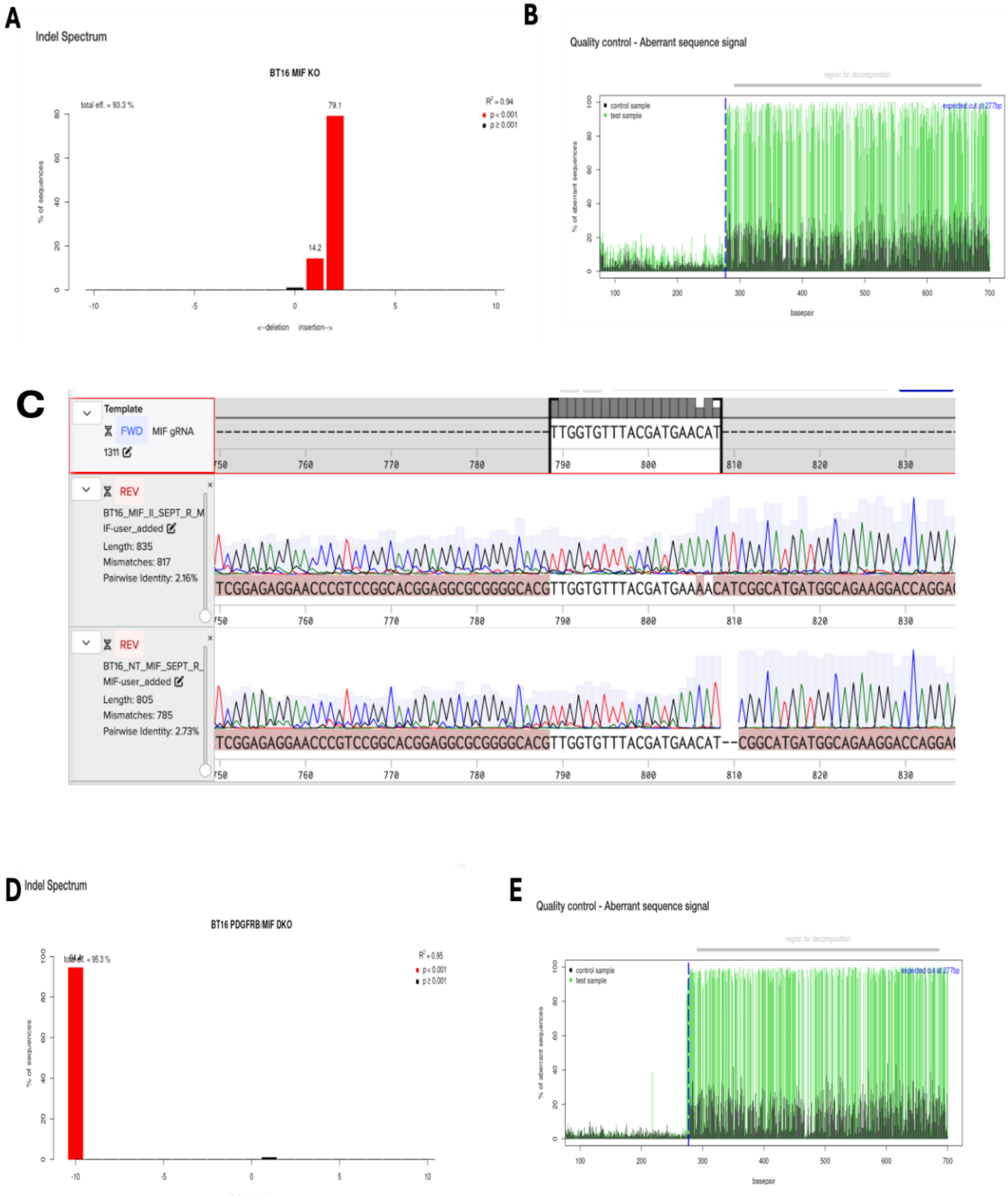
A
Indel Spectrum
BT16 MIF KO
total eff. = 93.3 %
R² = 0.94
p < 0.001
p ≥ 0.001
79.1
14.2
% of sequences
<--deletion insertion-->
B
Quality control - Aberrant sequence signal
region for decomposition
control sample
test sample
expected cut at 277bp
% of aberrant sequences
basepair
C
Template
FWD MIF gRNA
1311
TTGGTGTTTACGATGAACAT
REV
BT16_MIF_II_SEPT_R_MIF-user_added
Length: 835
Mismatches: 817
Pairwise Identity: 2.16%
TCGGAGAGGAACCCGTCCGGCACGGAGGCGCGGGGCACGTTGGTGTTTACGATGAAAACATCGGCATGATGGCAGAAGGACCAGGA
REV
BT16_NT_MIF_SEPT_R_MIF-user_added
Length: 805
Mismatches: 785
Pairwise Identity: 2.73%
TCGGAGAGGAACCCGTCCGGCACGGAGGCGCGGGGCACGTTGGTGTTTACGATGAACAT--CGGCATGATGGCAGAAGGACCAGGA
D
Indel Spectrum
BT16 PDGFRB/MIF DKO
total eff. = 95.3 %
R² = 0.95
p < 0.001
p ≥ 0.001
% of sequences
<--deletion insertion-->
E
Quality control - Aberrant sequence signal
region for decomposition
control sample
test sample
expected cut at 277bp
% of aberrant sequences
basepair

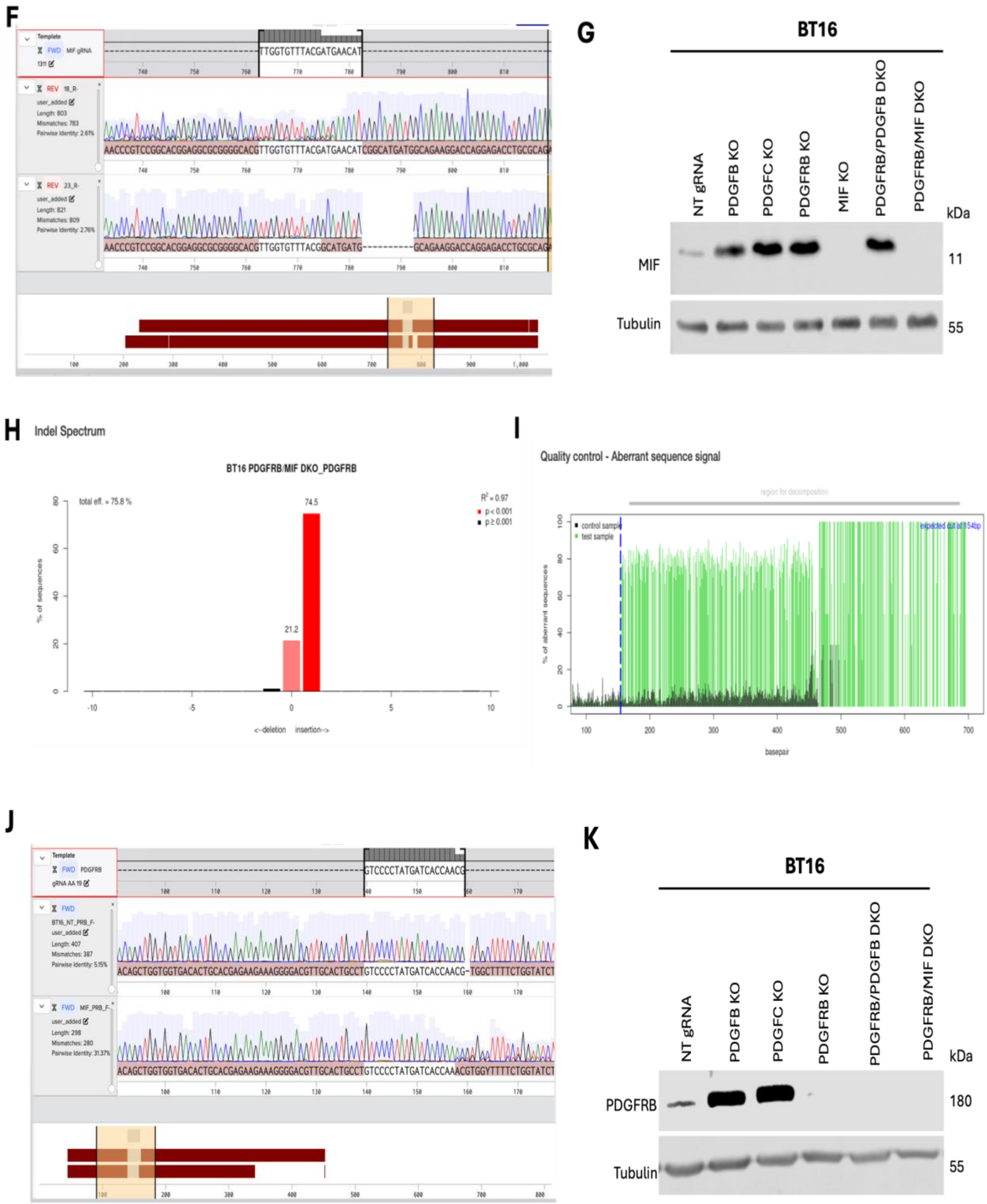
F
G
BT16
NT gRNA
PDGFB KO
PDGFC KO
PDGFRB KO
MIF KO
PDGFRB/PDGFB DKO
PDGFRB/MIF DKO
kDa
MIF
11
Tubulin
55
H
Indel Spectrum
BT16 PDGFRB/MIF DKO_PDGFRB
total eff. = 75.8 %
74.5
21.2
<--deletion insertion-->
% of sequences
I
Quality control - Aberrant sequence signal
region for decomposition
control sample
test sample
% of aberrant sequences
basepair
J
K
BT16
NT gRNA
PDGFB KO
PDGFC KO
PDGFRB KO
PDGFRB/PDGFB DKO
PDGFRB/MIF DKO
kDa
PDGFRB
180
Tubulin
55

L

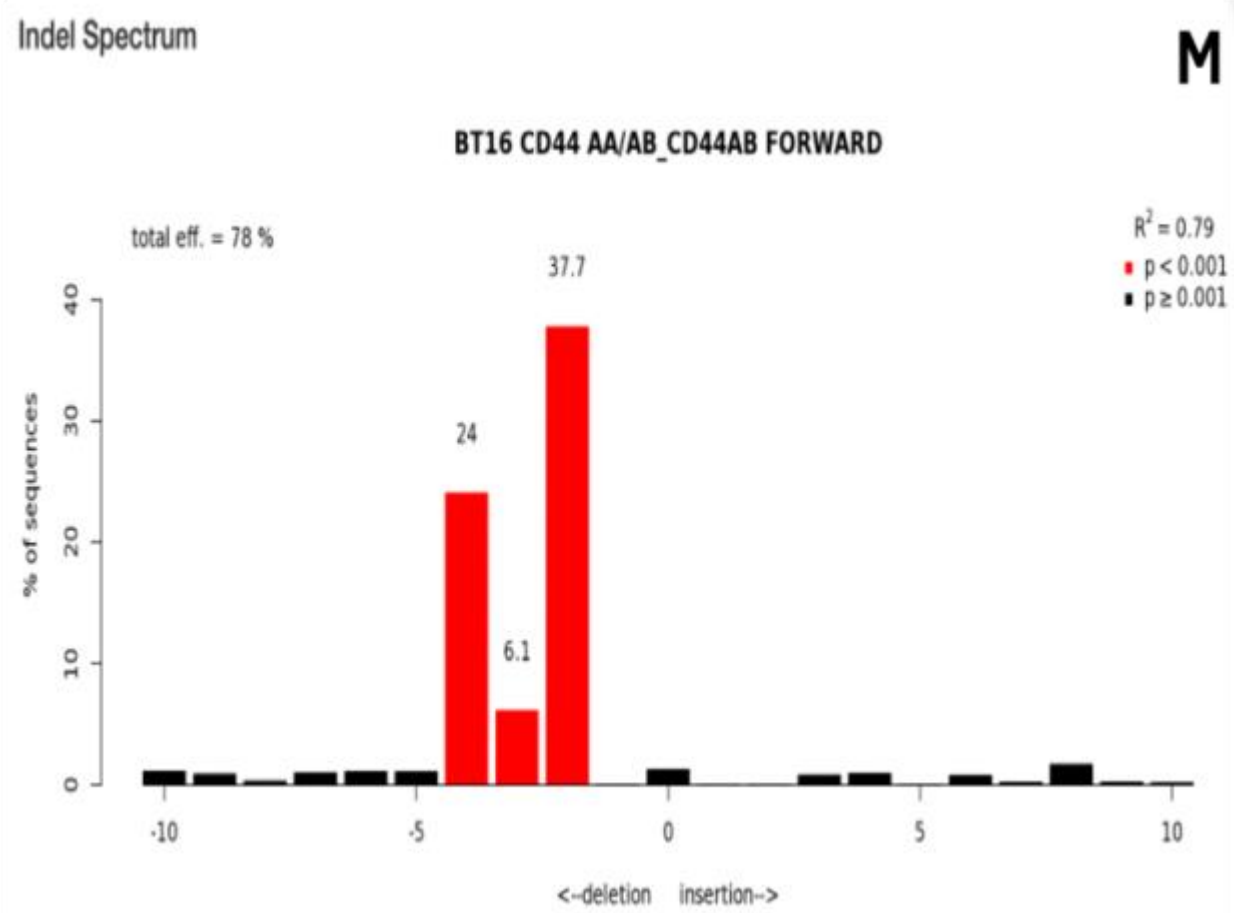
Indel Spectrum
BT16 CD44 AA/AB_CD44AB FORWARD
total eff. = 78 %
R^2 = 0.79
p < 0.001
p ≥ 0.001
37.7
24
6.1
% of sequences
<--deletion insertion-->

M

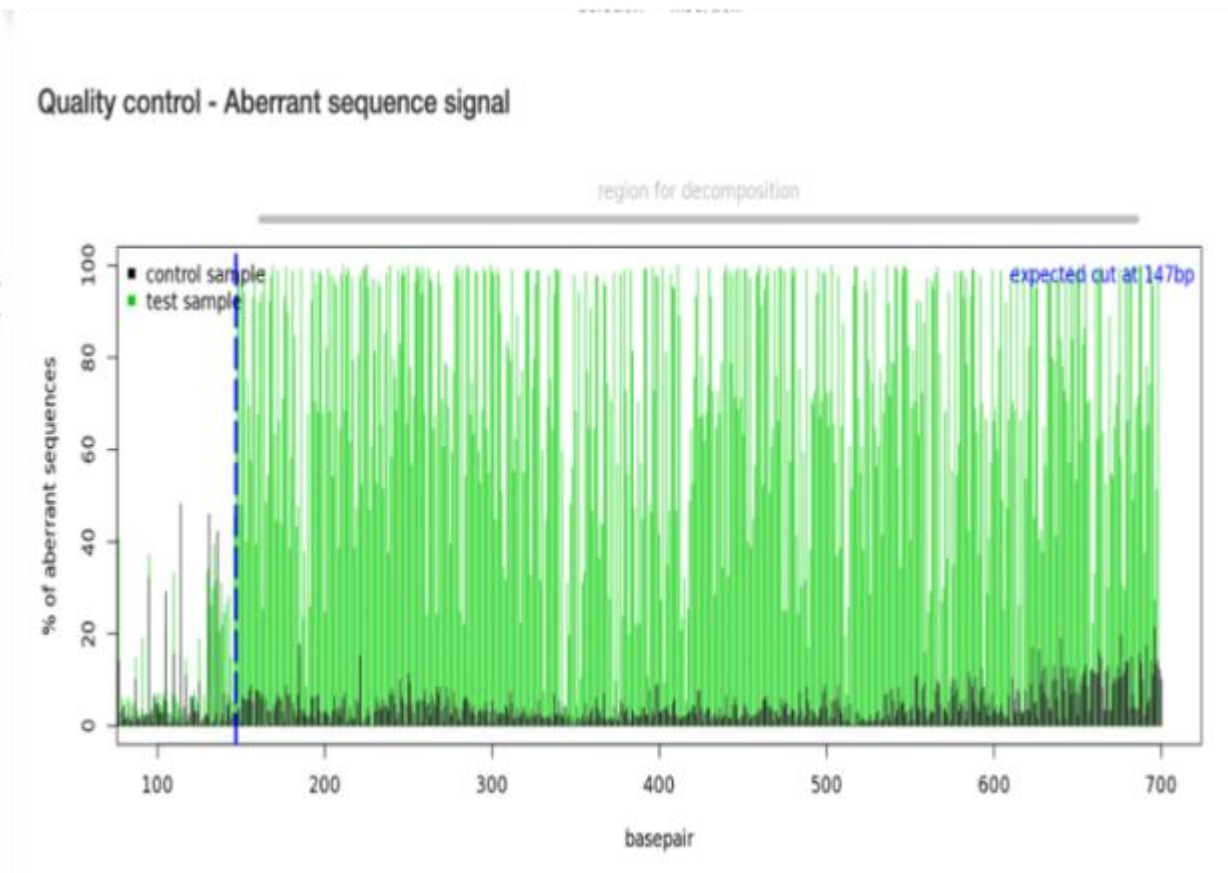
Quality control - Aberrant sequence signal
region for decomposition
control sample
test sample
expected cut at 147bp
% of aberrant sequences
basepair

N

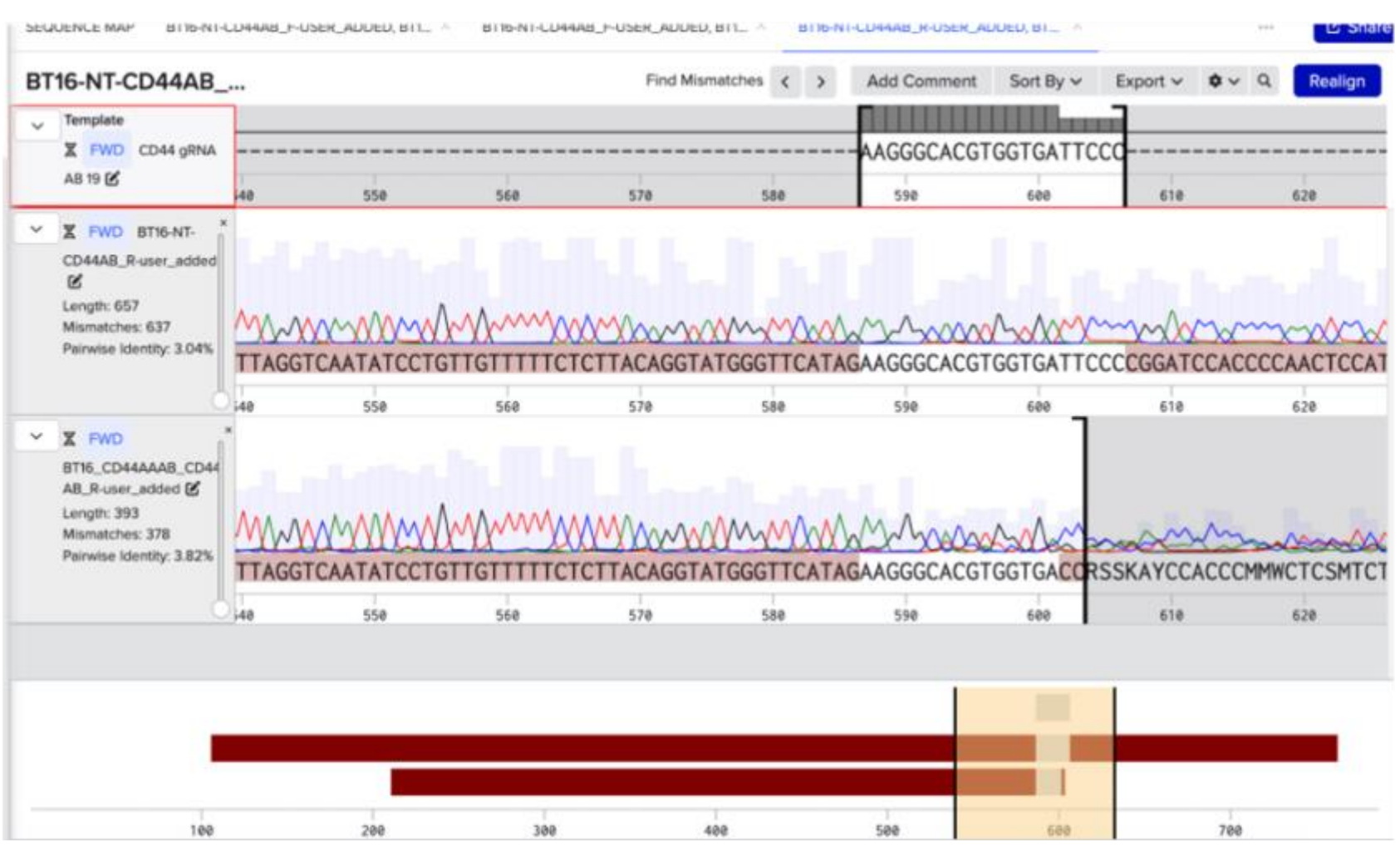
BT16-NT-CD44AB_...
Find Mismatches
Add Comment
Sort By
Export
Realign
Template
FWD CD44 gRNA AB 19
AAGGGCACGTGGTGATTCCC
FWD BT16-NT-CD44AB_R-user_added
Length: 657
Mismatches: 637
Pairwise Identity: 3.04%
TTAGGTCAATATCCTGTTGTTTTTCTCTTACAGGTATGGGTTCATAGAAGGGCACGTGGTGATTCCCCGGATCCACCCCAACTCCAT
FWD BT16_CD44AAAB_CD44AB_R-user_added
Length: 393
Mismatches: 378
Pairwise Identity: 3.82%
TTAGGTCAATATCCTGTTGTTTTTCTCTTACAGGTATGGGTTCATAGAAGGGCACGTGGTGACCRSSKAYCCACCCMMWCTCSMTCT

O

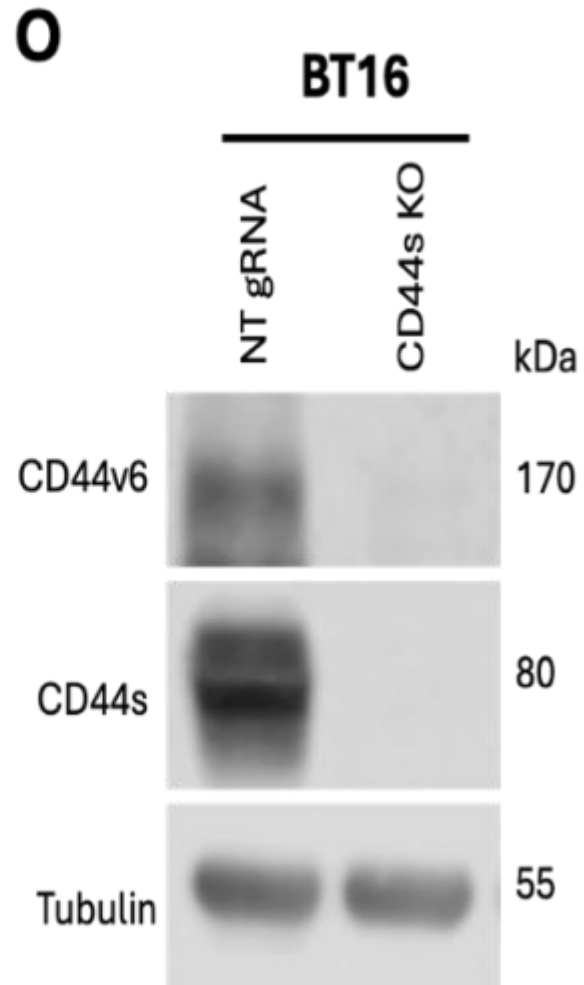
BT16
NT gRNA
CD44s KO
kDa
CD44v6
170
CD44s
80
Tubulin
55

P

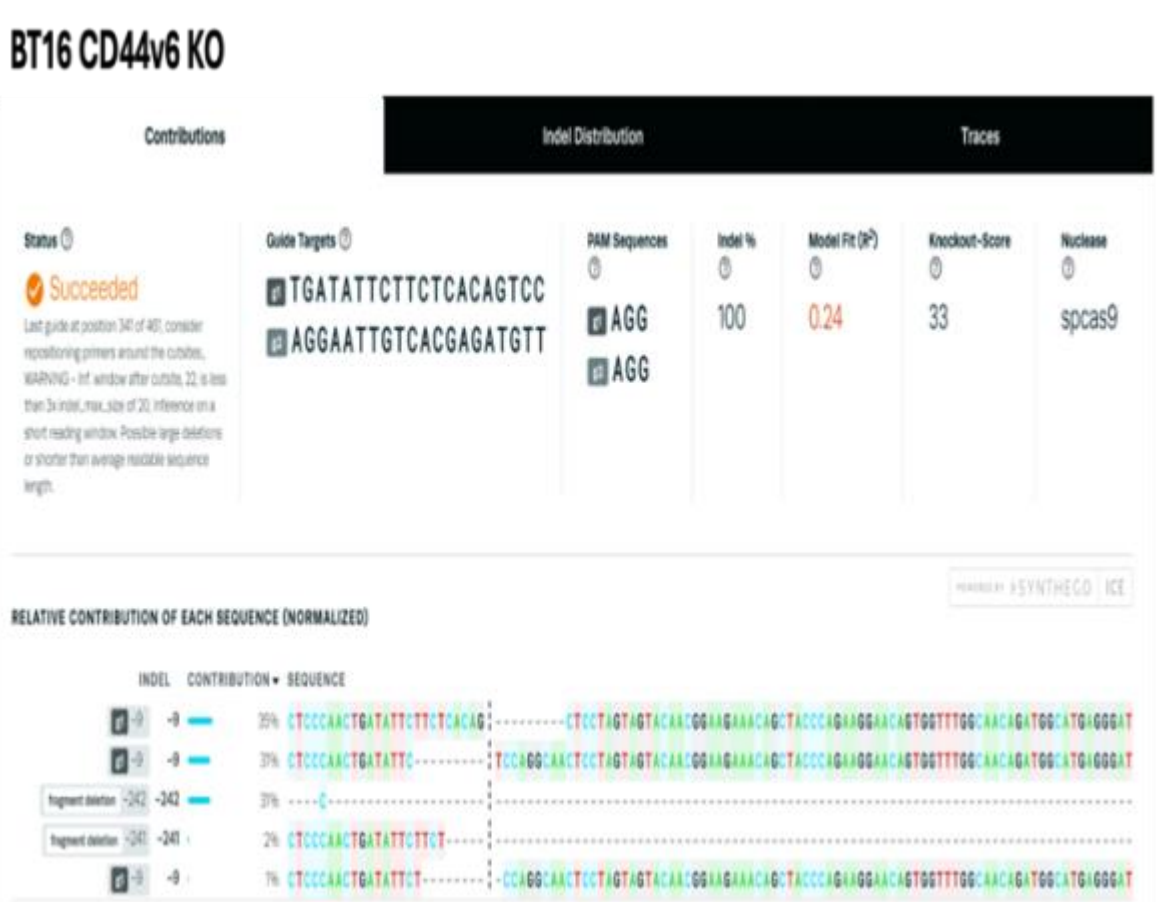
BT16 CD44v6 KO
Contributions
Indel Distribution
Traces
Succeeded
TGATATTCTTCTCACAGTCC
AGGAATTGTCACGAGATGTT
AGG
AGG
100
0.24
33
spcas9
RELATIVE CONTRIBUTION OF EACH SEQUENCE (NORMALIZED)

Q

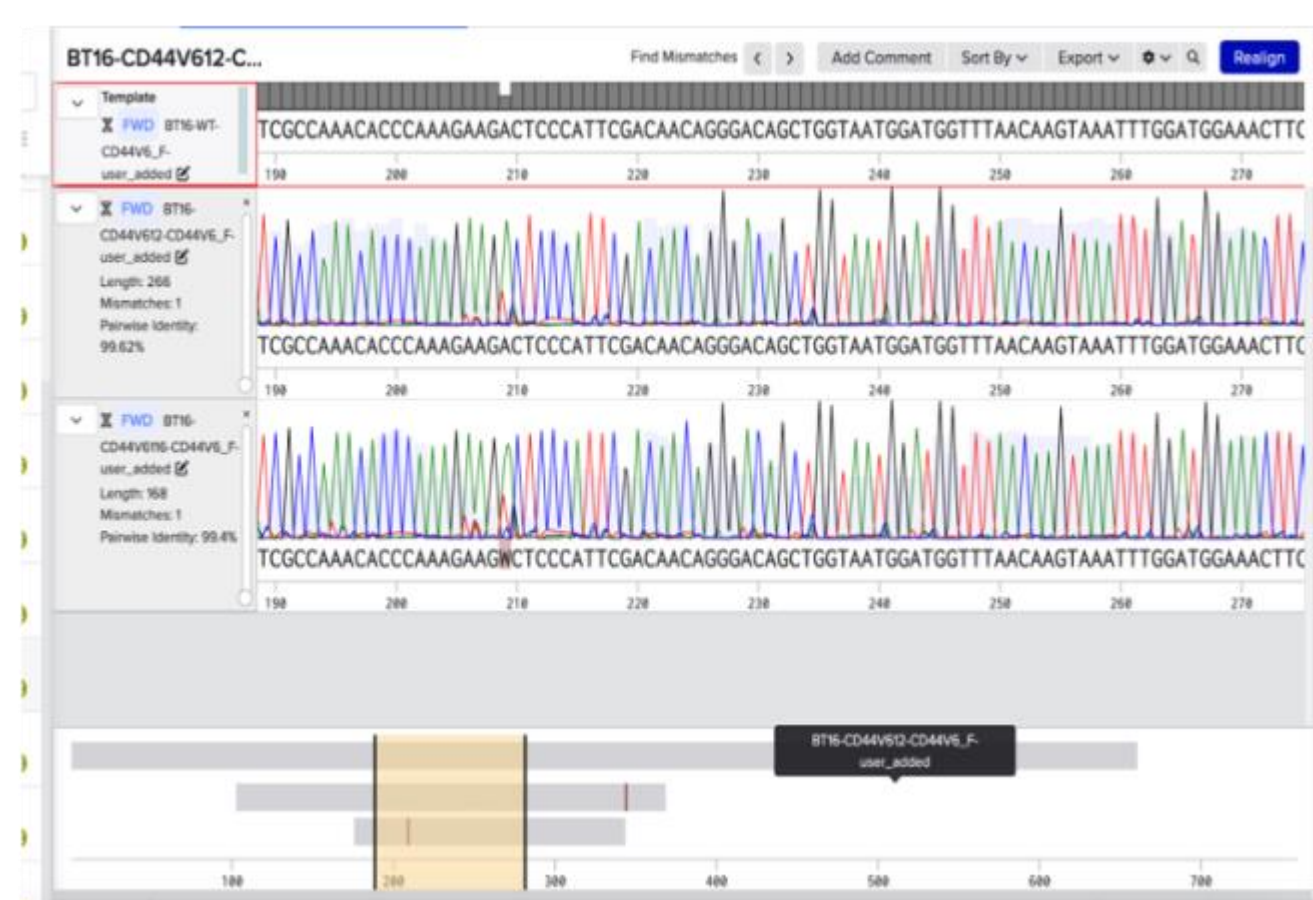
BT16-CD44V612-C...
Find Mismatches
Add Comment
Sort By
Export
Realign
Template
TCGCCAAACACCCAAAGAAGACTCCCATTCGACAACAGGGACAGCTGGTAATGGATGGTTTAACAAGTAAATTTGGATGGAAACTTC
TCGCCAAACACCCAAAGAAGACTCCCATTCGACAACAGGGACAGCTGGTAATGGATGGTTTAACAAGTAAATTTGGATGGAAACTTC
TCGCCAAACACCCAAAGAAGNCTCCCATTCGACAACAGGGACAGCTGGTAATGGATGGTTTAACAAGTAAATTTGGATGGAAACTTC

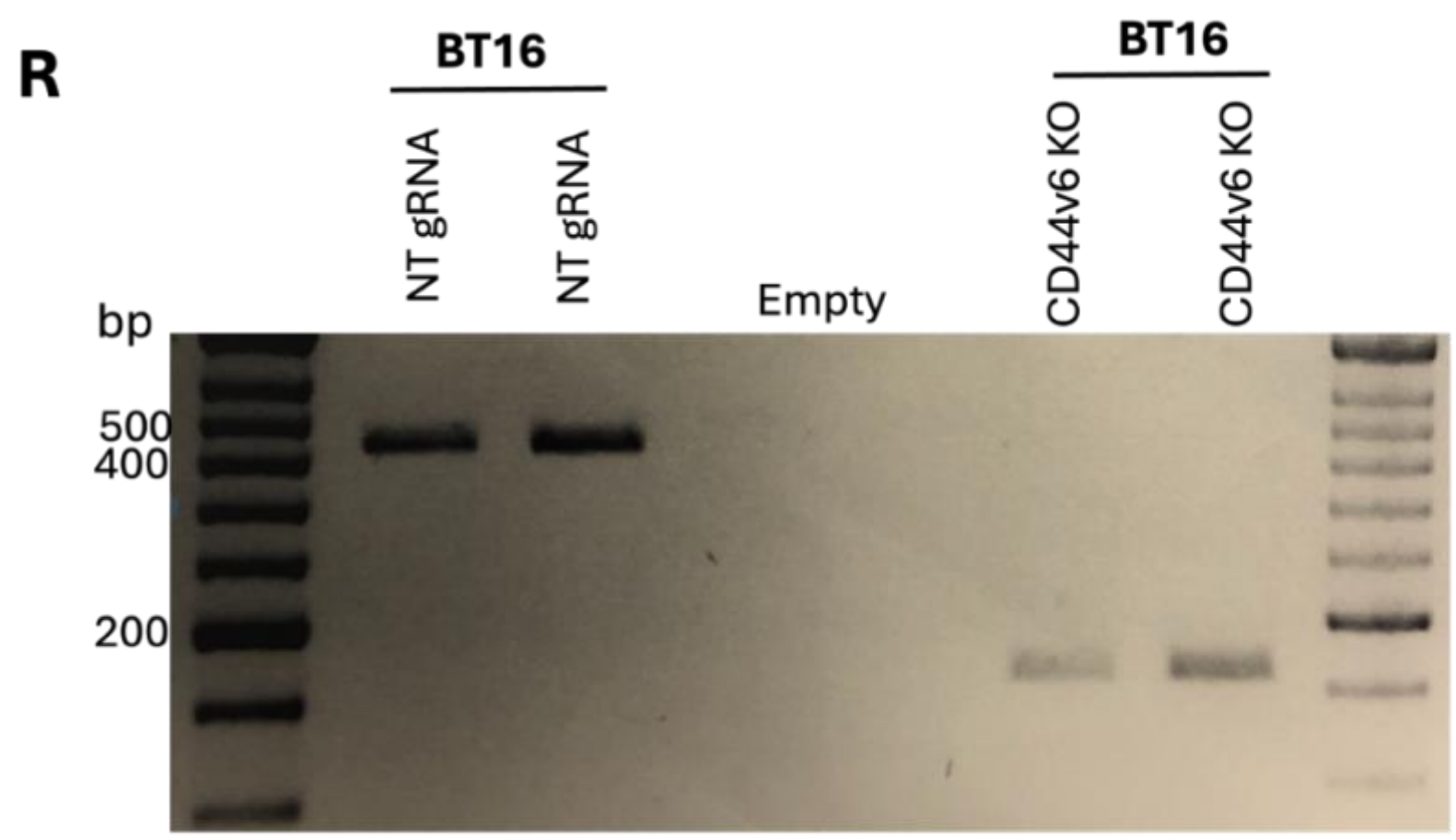

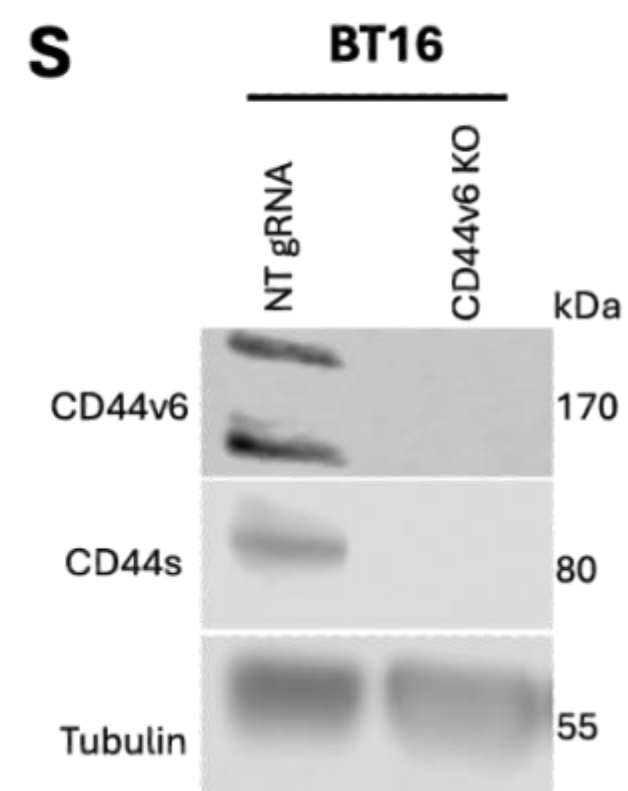

Supplementary Fig. 4. Validation of BT16 MIF KO, PDGFRB/MIF DKO, CD44s KO, and CD44v6 KO

- **A-C.** Genomic validation of BT16 MIF KO.
- **D-F.** Genomic validation of MIF KO in BT16 PDGFRB/MIF DKO.
- **G.** Western blot validation of MIF protein loss in BT16 MIF KO and BT16 PDGFRB/MIF DKO.
- **H-J.** Genomic validation of PDGFRB KO in BT16 PDGFRB/MIF DKO.
- **K.** Western blot validation of PDGFRB protein loss in BT16 PDGFRB/MIF DKO.
- **L-N.** Genomic validation of BT16 CD44s KO.
- **O.** Western blot validation of CD44s protein loss in BT16 CD44s KO.
- **P.** Interference of CRISPR edits (ICE) analysis of Sanger sequences of BT16 CD44v6 KO relative to NT gRNA, using Synthego ICE analysis tool (ice.synthego.com). The analysis shows ~241 bp fragment deletion, corresponding approximately to the region around CD44 exon 11 (or variable exon 6), as described by Lobo *et al.(3)*
- **Q.** Sanger sequences alignment of BT16 NT gRNA and two BT16 CD44v6 KO samples, showing about ~250 bp shorter sequences in BT16 CD44v6 KOs.
- **R.** PCR product analysis of BT16 NT gRNA and BT16 CD44v6 KO, using primers flanking the region including intron v5, exon v6, and intron v6, as described by Lobo *et al.*(3) Two biological replicates were used. BT16 CD44v6 product is smaller relative to that of BT16 NT gRNA, consistent with the fragment deletion.
- **S.** Western blot validation of CD44v6 protein loss in BT16 CD44v6 KO. Interestingly, BT16 CD44v6 KO showed a loss of CD44s protein, despite no change in CD44 mRNA levels (Fig. 3O, P)

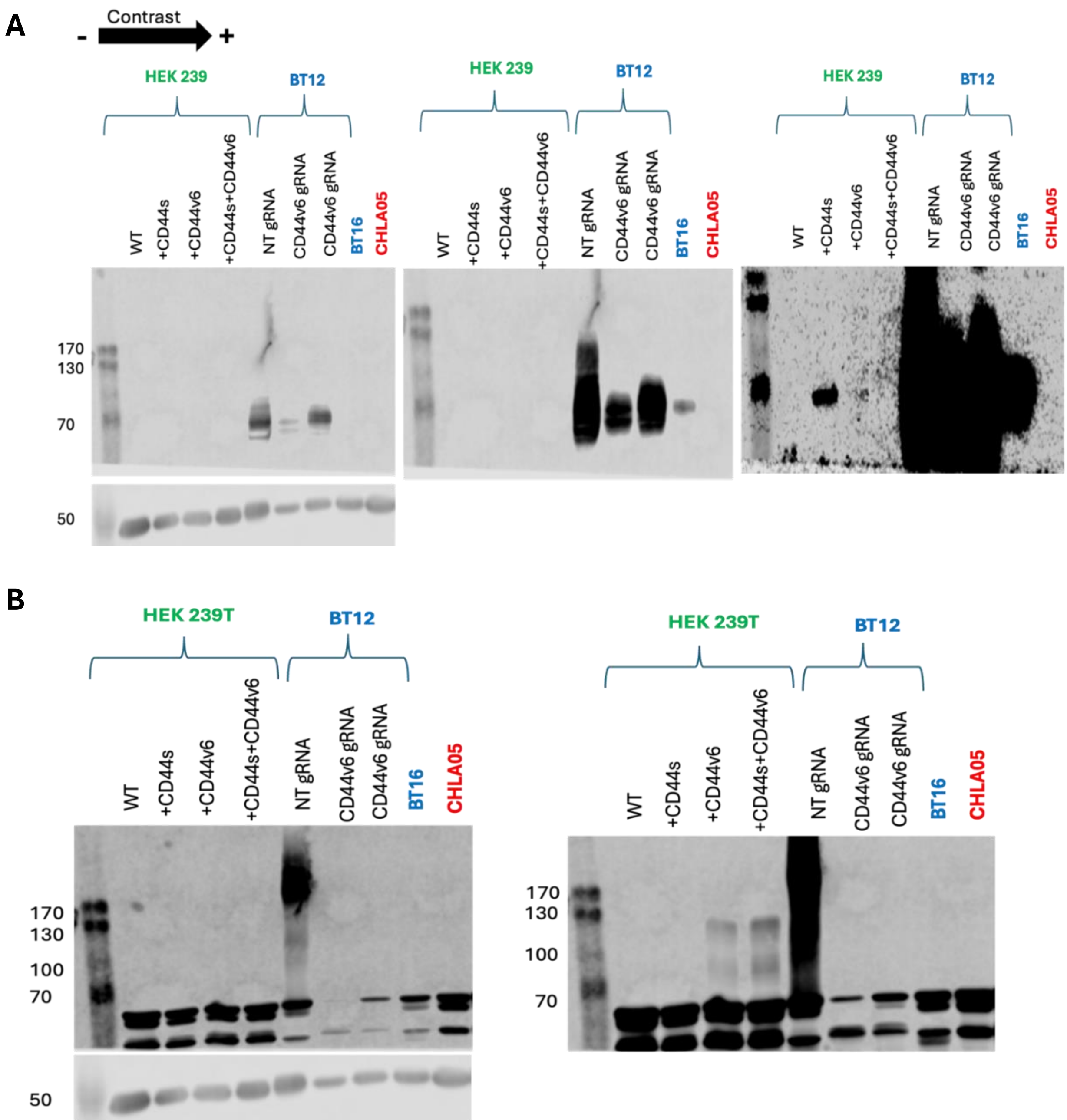

Supplementary Fig. 5. CD44 isoform-specific detection validation

CD44s **(A)** and CD44v6 **(B)** expression in HEK239T, BT12, BT16, and CHLA05 cells. The images from left to right represent an increasing contrast. HEK293T cells were transfected with CD44s expression plasmid (pPGK-T7/2-CD44s; Addgene Plasmid #137812), CD44v6 expression plasmid (pPGK-T7/2-CD44v6; Addgene Plasmid #137816), or a combination of both, as described by Mielgo *et al.*(4) BT12 NT gRNA was included as a positive control for CD44s and CD44v6 expression, BT12 CD44s KO was included as a negative control for CD44s expression, and BT12 CD44v6 KO was included as a negative control for CD44v6 expression. BT16 was included as a control for low CD44v6 expression. CHLA05 was included as a negative control for CD44s and CD44v6 expression. CD44 antibody (CST #37259) detects CD44s only in CD44s+ HEK293T cells, without cross-reacting with CD44v6 expressed by CD44v6+ HEK293T cells.

Conversely, CD44v6 antibody (Invitrogen #33-6700) detects CD44v6 without cross-reacting with CD44s. Interestingly, HEK293T transfected with both pPGK-T7/2-CD44s and pPGK-T7/2-CD44v6, expressed only CD44v6, which could be due to varying transfection efficiency. CD44v6 band size was lower in CDD4v6+ HEK293T cells relative to BT12 cells, which could be due to differences in CD44v6 glycosylation.

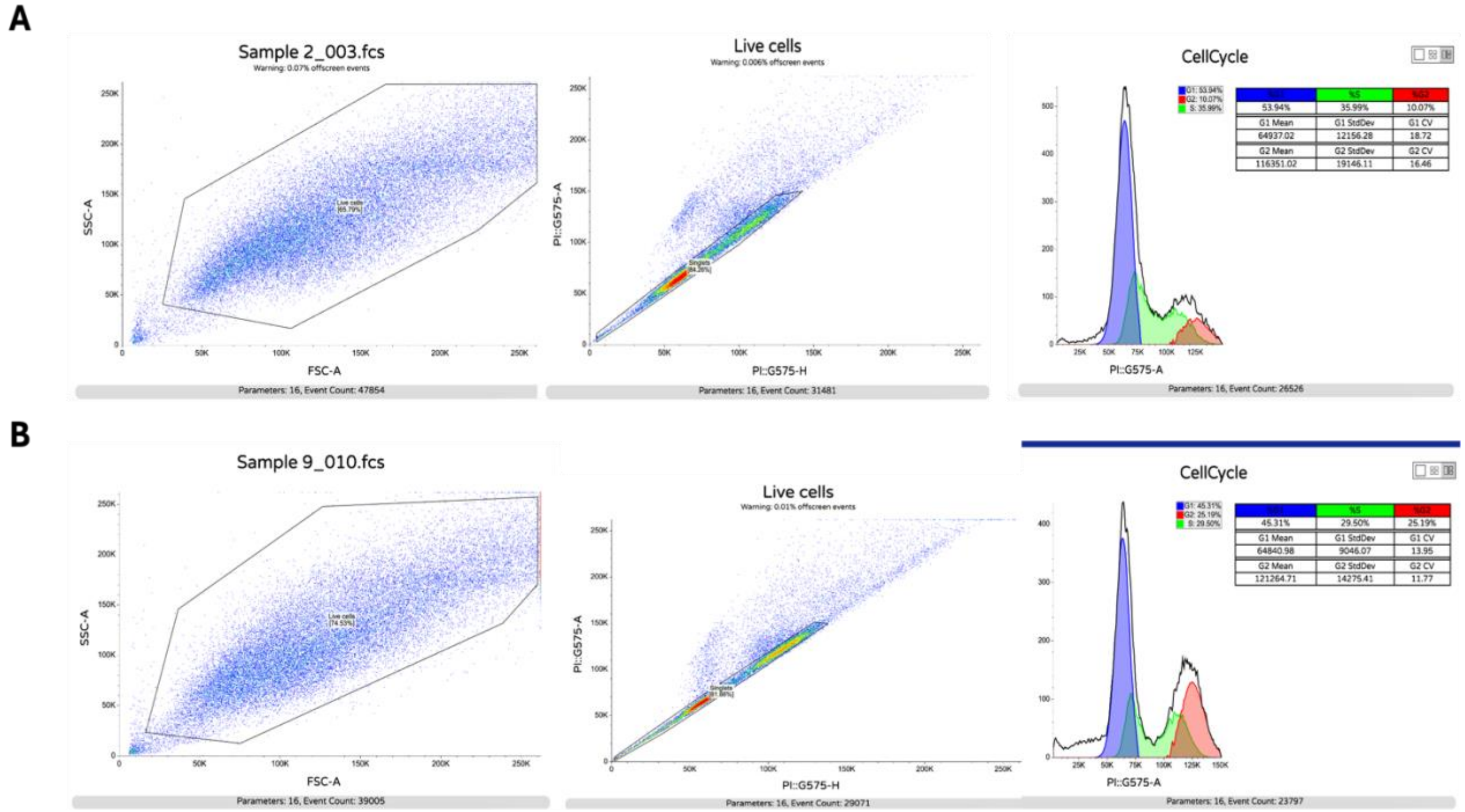

Supplementary Fig. 6. Representative gating strategy for cell cycle analysis

A. BT12 NT gRNA control cells and **B.** CD44v6 KO cells. Cells were gated to exclude debris based on forward scatter (FSC-A) and side scatter (SSC-A) (left panels). Singlets were discriminated from doublets using PI pulse height (PI-H) and pulse area (PI-A) (middle panels). Cell cycle distribution was determined from PI intensity histograms of singlet populations, with gates defining G1 (blue), S (green), and G2/M (red) phases (right panels).

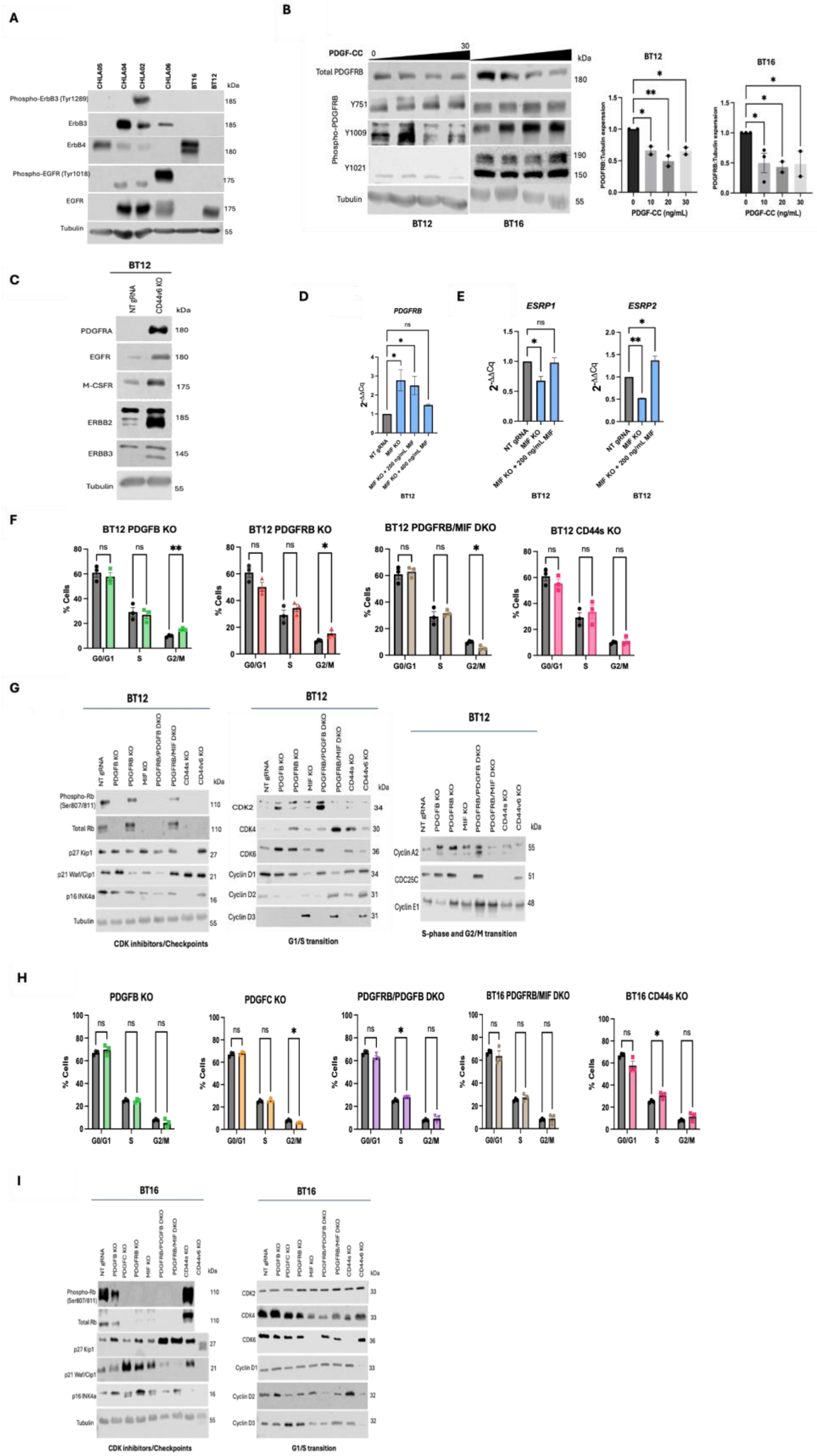
A
CHLA05
CHLA04
CHLA02
CHLA06
BT16
BT12
kDa
Phospho-ErbB3 (Tyr1289)
ErbB3
ErbB4
Phospho-EGFR (Tyr1018)
EGFR
Tubulin
B
PDGF-CC
Total PDGFRB
Phospho-PDGFRB
Y751
Y1009
Y1021
Tubulin
BT12
BT16
PDGFRB:Tubulin expression
PDGF-CC (ng/mL)
C
NT gRNA
CD44v6 KO
PDGFRA
EGFR
M-CSFR
ERBB2
ERBB3
Tubulin
D
PDGFRB
E
ESRP1
ESRP2
F
BT12 PDGFB KO
BT12 PDGFRB KO
BT12 PDGFRB/MIF DKO
BT12 CD44s KO
% Cells
G0/G1
S
G2/M
G
Phospho-Rb (Ser807/811)
Total Rb
p27 Kip1
p21 Waf/Cip1
p16 INK4a
CDK inhibitors/Checkpoints
CDK2
CDK4
CDK6
Cyclin D1
Cyclin D2
Cyclin D3
G1/S transition
Cyclin A2
CDC25C
Cyclin E1
S-phase and G2/M transition
H
PDGFB KO
PDGFC KO
PDGFRB/PDGFB DKO
BT16 PDGFRB/MIF DKO
BT16 CD44s KO
I
BT16

Supplementary Fig. 7.

- **A.** Expression of EGFR, phospho-EGFR, ErbB3, phospho-ErbB3, and ErbB4 (by western blot) in ATRT cell lines.
- **B.** The effects of PDGF-CC stimulation (0, 10, 20, and 30 ng/mL for 5 minutes) on PDGFRB and phospho-PDGFRB protein expression in BT12, and BT16 cells (left). Densitometry quantification of total PDGFRB (right). (n=2; Error bars represent ± SEM; One-way ANOVA; *$p< 0.05$, **$p < 0.01$, ***$p < 0.001$, ****$p < 0.0001$).
- **C.** Effects of BT12 CD44s KO and CD44v6 KO on PDGFRA, EGFR, M-CSFR, ERBB2, and ERBB3 by western blot.
- **D.** *PDGFRB* mRNA expression by qRT-PCR under MIF stimulation in BT12 cells (200 ng/mL and 400 ng/mL for 24 hours) (n=2; Error bars represent ± SEM; One-way ANOVA; *$p< 0.05$, **$p < 0.01$, ***$p < 0.001$, ****$p < 0.0001$).
- **E.** *ESPR1* and *ESRP2* mRNA expression by qRT-PCR in BT12 MIF KO without and with MIF stimulation. (n=2; Error bars represent ± SEM; One-way ANOVA; *$p< 0.05$, **$p < 0.01$, ***$p < 0.001$, ****$p < 0.0001$).
- **F, H.** Cell cycle distribution in PDGFB KO, PDGFC KO, PDGFRB KO, PDGFRB/PDGFB DKO, and CD44s KO relative to NT gRNA BT12 **(F)** and BT16 **(H)** measured by PI staining by flow cytometry. Cell cycle data represent mean ± SEM of 3 independent biological replicates performed in technical singlets (3 data points per condition) (One-way ANOVA with Dunnett's post-hoc test).
- **G, I.** Cell cycle markers protein expression in BT12 **(G)** and BT16 **(I)** NT gRNA, PDGFB KO, PDGFRB KO, MIF KO, PDGFRB/PDGFB DKO, PDGFRB/MIF DKO, CD44s KO, and CD44v6 KO by western blot. Markers were grouped by cell cycle phase/transition.

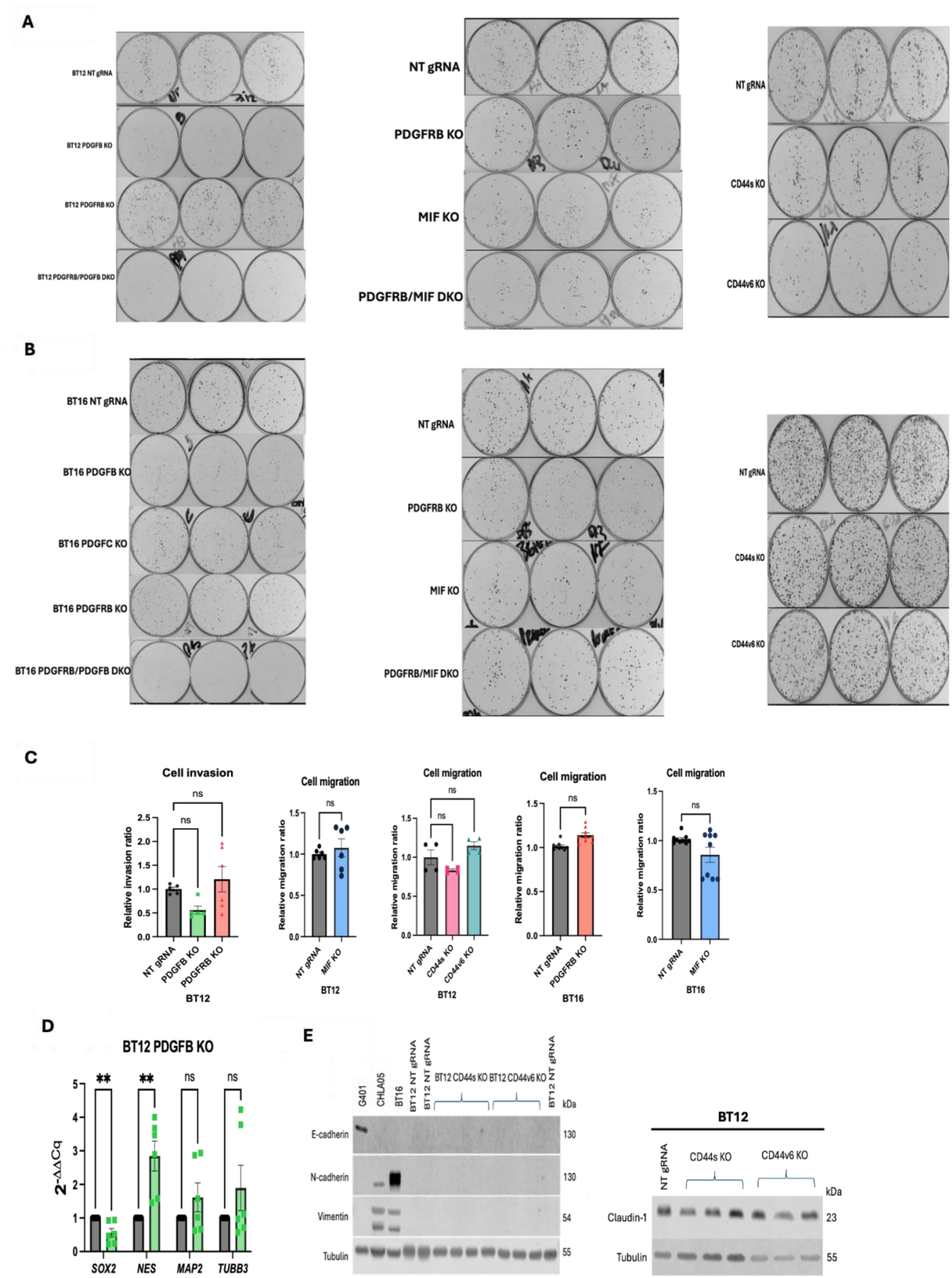
A
BT12 NT gRNA
BT12 PDGFB KO
BT12 PDGFRB KO
BT12 PDGFRB/PDGFB DKO
NT gRNA
PDGFRB KO
MIF KO
PDGFRB/MIF DKO
NT gRNA
CD44s KO
CD44v6 KO
B
BT16 NT gRNA
BT16 PDGFB KO
BT16 PDGFC KO
BT16 PDGFRB KO
BT16 PDGFRB/PDGFB DKO
NT gRNA
PDGFRB KO
MIF KO
PDGFRB/MIF DKO
NT gRNA
CD44s KO
CD44v6 KO
C
Cell invasion
Relative invasion ratio
NT gRNA
PDGFB KO
PDGFRB KO
BT12
Cell migration
Relative migration ratio
NT gRNA
MIF KO
BT12
Cell migration
Relative migration ratio
NT gRNA
CD44s KO
CD44v6 KO
BT12
Cell migration
Relative migration ratio
NT gRNA
PDGFRB KO
BT16
Cell migration
Relative migration ratio
NT gRNA
MIF KO
BT16
ns
D
BT12 PDGFB KO
2-ΔΔCq
**
ns
SOX2
NES
MAP2
TUBB3
E
G401
CHLA05
BT16
BT12 NT gRNA
BT12 NT gRNA
BT12 CD44s KO
BT12 CD44v6 KO
BT12 NT gRNA
kDa
E-cadherin
N-cadherin
Vimentin
Tubulin
130
130
54
55
BT12
NT gRNA
CD44s KO
CD44v6 KO
kDa
Claudin-1
Tubulin
23
55

Supplementary Figure 8.

- **A, B.** Colony count in PDGFB KO, PDGFC KO, PDGFRB KO, PDGFRB/PDGFB DKO, MIF KO, PDGFRB/MIF DKO, CD44s KO, and CD44v6 KO relative to NT gRNA. Colony formation data represent mean ± SEM of 3 independent biological replicates performed in technical triplicates (minimum 9 data points per condition). The images represent one of the replicates (One-way ANOVA with Dunnett's post-hoc test).
- **C.** Cell invasion/migration in PDGFB KO, PDGFRB KO, MIF KO, CD44s KO, and CD44v6 KO relative to NT gRNA measured by Matrigel/FluroBlok assay (Em/Ex 494/517 nm). Cell invasion and migration data represent mean ± SEM of 3 independent biological replicates performed in technical duplicates (6 data points per condition; except for BT12 CD44 KOs migration assay: 2 biological replicates and 4 data points) (One-way ANOVA with Dunnett's post-hoc test; Two-tailed Student's t-test).
- **D.** Neural stemness (*SOX2* and *NES*) and differentiation marker (*TUBB3* and *MAP2*) expression in BT12 PDGFB KO relative to NT gRNA BT12 by qRT-PCR. (n=3; Error bars represent ± SEM; Two-tailed Student's t-test; **p*< 0.05, ***p* < 0.01, ****p* < 0.001, *****p* < 0.0001).
- **E.** EMT marker expression in BT12 CD44s KO and CD44v6 KO relative to NT gRNA by western blot. BT16 was included as a positive control for N-cadherin expression (N-cadherin high); CHLA05 as a control for low N-cadherin expression; G401, a kidney MRT cell line, as a positive control for E-cadherin expression. Three independent biological replicates were used from BT12 CD44s KO and CD44v6 KO, corresponding to the three lanes grouped under the same brace.

Supplementary Figure 9.

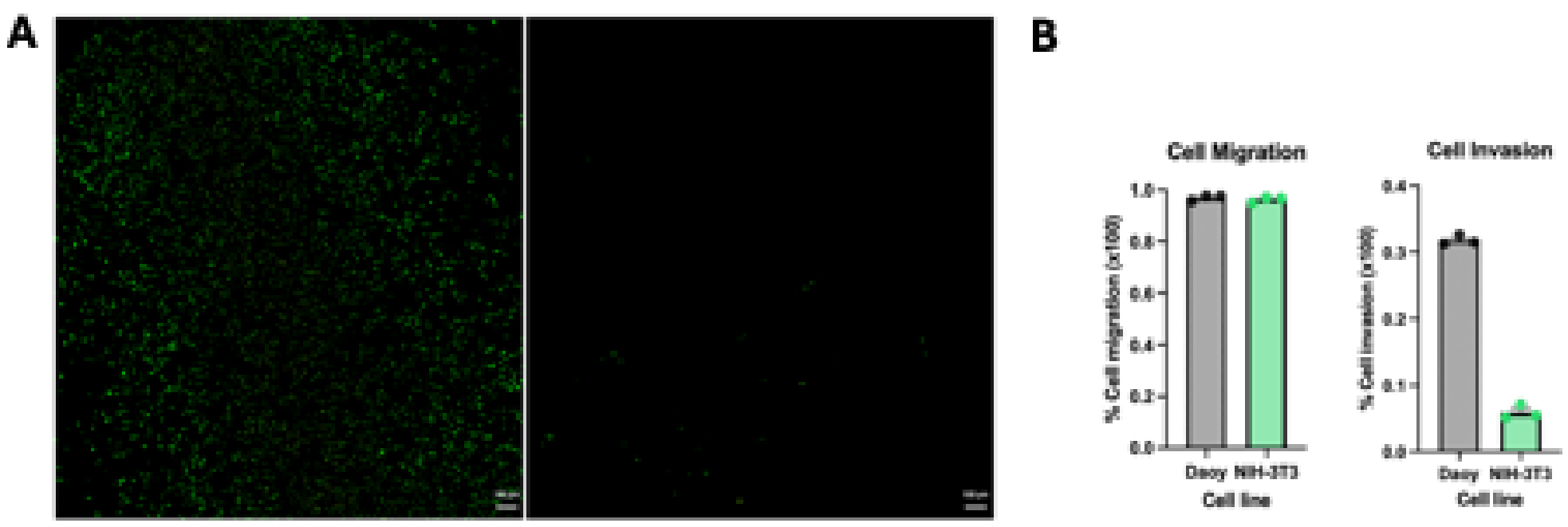

- **A.** Representative epifluorescence micrographs of NIH-3T3 cells cultured on FluroBlok inserts without Matrigel (left) or with Matrigel (right). NIH-3T3 cells migrated through uncoated Fluoroblok inserts after 24 hours; however, NIH-3T3 cells showed minimal invasion through Matrigel-coated inserts (Chemoattractant: DMEM + 30%FBS). Migrated (left) and invaded (right) cells were labelled with Calcein-AM and imaged using Nikon Epifluorescence Microscope at x20. Scale bar =100 µm. **B.** Cell invasion/migration in Daoy (migratory and invasive) and NIH-3T3 (migratory but not invasive) cells measured by FluroBlock assay (Em/Ex 494/517 nm) (n=3; Error bars represent ± SEM).

References

1. Torchia J, Golbourn B, Feng S, Ho KC, Sin-Chan P, Vasiljevic A, et al. Integrated (epi)-Genomic Analyses Identify Subgroup-Specific Therapeutic Targets in CNS Rhabdoid Tumors. Cancer Cell. 2016 Dec 12;30(6):891–908. doi:10.1016/j.ccell.2016.11.003 PubMed PMID: 27960086.
2. Yamao T, Matsumura Y, Shimada Y, Moriya Y, Sugihara KI, Akasu T, et al. Abnormal expression of CD44 variants in the exfoliated cells in the feces of patients with colorectal cancer. Gastroenterology. 1998;114(6):1196–205. doi:10.1016/S0016-5085(98)70425-1 PubMed PMID: 9609756.

3. Lobo S, Pereira C, Oliveira C, Almeida GM. Skipping Exon-v6 from CD44v6-Containing Isoforms Influences Chemotherapy Response and Self-Renewal Capacity of Gastric Cancer Cells. Cancers (Basel). 2020 Sep 1;12(9):2378. doi:10.3390/CANCERS12092378 PubMed PMID: 32842638.
4. Mielgo A, van Driel M, Bloem A, Landmann L, Günthert U. A novel antiapoptotic mechanism based on interference of Fas signaling by CD44 variant isoforms. Cell Death Differ. 2006 Mar;13(3):465–77. doi:10.1038/SJ.CDD.4401763 PubMed PMID: 16167069.
